# From Unregulated Networks to Designed Microstructures: Introducing Heterogeneity at Different Length Scales in Photopolymers for Additive Manufacturing

**DOI:** 10.1021/acs.chemrev.3c00570

**Published:** 2024-03-28

**Authors:** Mojtaba Ahmadi, Katharina Ehrmann, Thomas Koch, Robert Liska, Jürgen Stampfl

**Affiliations:** † Institute of Materials Science and Technology, Technische Universität Wien, Getreidemarkt 9BE, 1060 Vienna, Austria; ‡ Institute of Applied Synthetic Chemistry, Technische Universität Wien, Getreidemarkt 9/163, 1060 Vienna, Austria

## Abstract

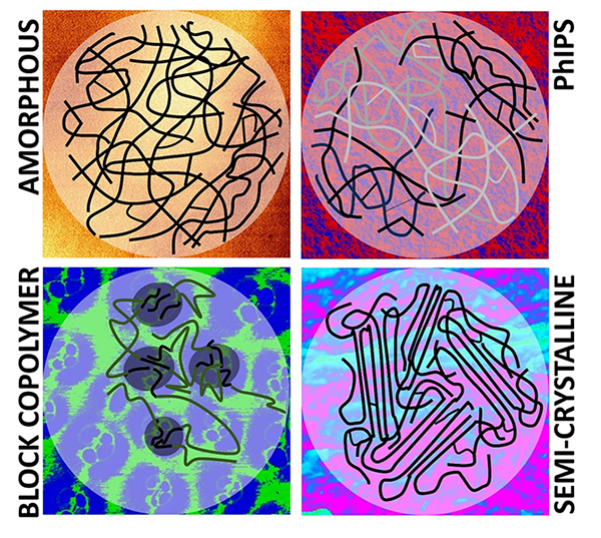

Photopolymers have been optimized as protective and decorative coating materials for decades. However, with the rise of additive manufacturing technologies, vat photopolymerization has unlocked the use of photopolymers for three-dimensional objects with new material requirements. Thus, the originally highly cross-linked, amorphous architecture of photopolymers cannot match the expectations for modern materials anymore, revealing the largely unanswered question of how diverse properties can be achieved in photopolymers. Herein, we review how microstructural features in soft matter materials should be designed and implemented to obtain high performance materials. We then translate these findings into chemical design suggestions for enhanced printable photopolymers. Based on this analysis, we have found microstructural heterogenization to be the most powerful tool to tune photopolymer performance. By combining the chemical toolbox for photopolymerization and the analytical toolbox for microstructural characterization, we examine current strategies for physical heterogenization (fillers, inkjet printing) and chemical heterogenization (semicrystalline polymers, block copolymers, interpenetrating networks, photopolymerization induced phase separation) of photopolymers and put them into a material scientific context to develop a roadmap for improving and diversifying photopolymers’ performance.

## 1 Introduction

Additive manufacturing technologies (AMTs) offer the possibility of shaping complex metallic, ceramic, and polymeric structures. They have been used in a large number of applications including digital dentistry, personalized implants, parts for aerospace systems, personalized consumer products, and 3D-printed buildings.^1−4^

**Figure 1:**
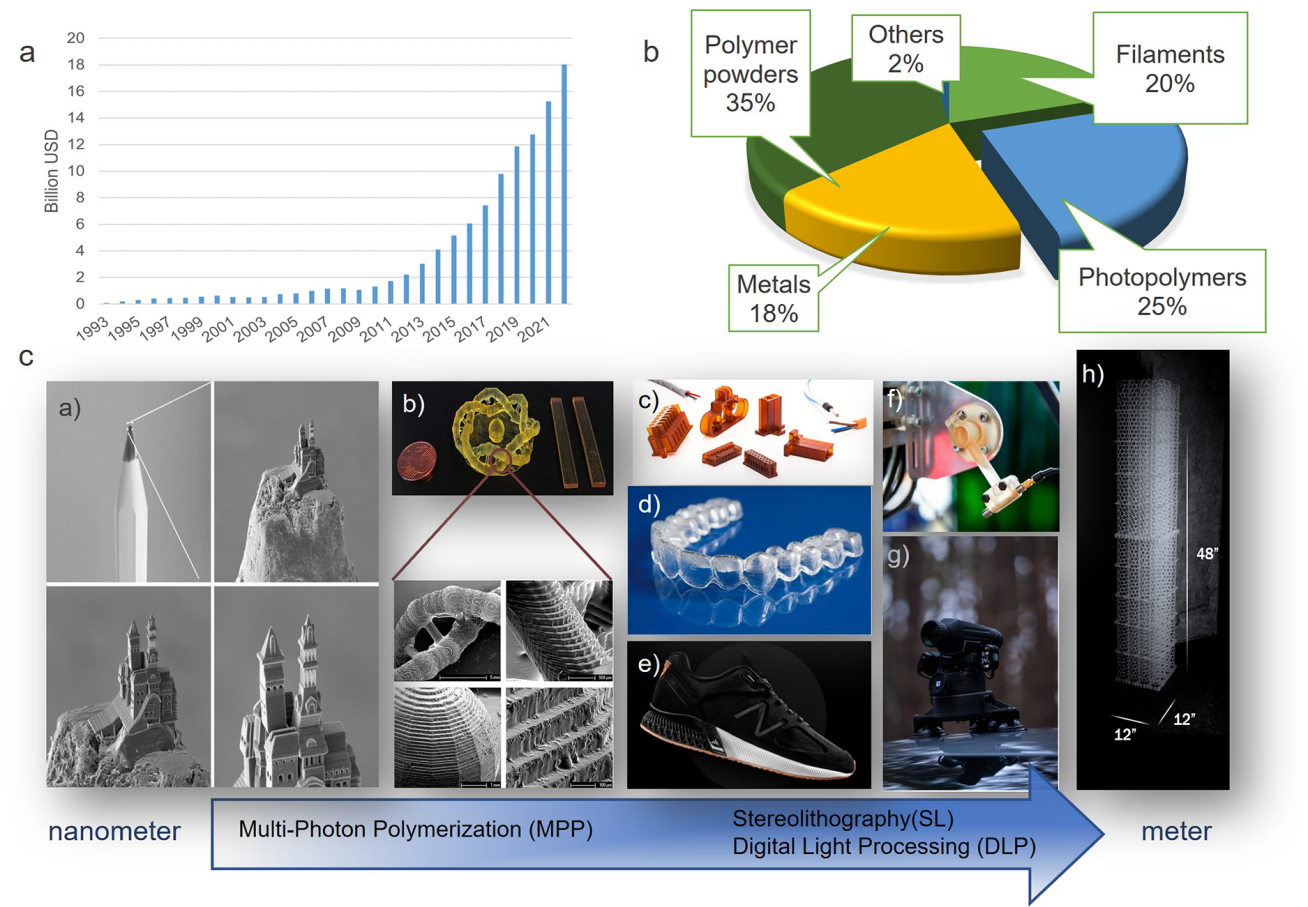
(a) Revenue of the additive manufacturing technology (AMT) industry including services and products since 1993, and b) relative revenue for different materials used in the AMT industry in 2022.^7^ c) Additively manufactured parts via photopolymerization on all length scales, enabled by several vat photopolymerization techniques (multiphoton polymerization, stereolithography, digital light processing): a) Acrylate-based castle, printed by two-photon polymerization, adapted with permission from ref (10). Copyright 2016 John Wiley and Sons. b) High-strength 3D printed parts produced by Hot Lithography technique. Adapted with permission from ref (11). Copyright 2018 American Chemical Society. c) Flame-resistant photopolymers for electrical applications, courtesy of Cubicure GmbH. Copyright Fotostudio Huger. d) SL printed aligners for teeth misalignment corrections. Reprinted with permission from ref (12) Copyright 2012 Springer Nature. e) 3D printed piece in the heel of New Balance 990 running shoe, f) mounts of pick-and-place robot sensors intended to work in high-temperature environments. g) Outdoor tripod camera Xspecter T-Crow XRII with SL printed gears and axes. Images by the courtesy of Formlabs. h) Massive complex 3D printed structure. Image by the courtesy of Chad Mirkin Lab.

Such ever-expanding applications of AMTs are the result of innovative hardware concepts, user-friendly computer-aided design (CAD) software, and printable materials with excellent thermomechanical properties.^5^ Additionally, AMTs strongly support the digitalization of the manufacturing industry.^6^ Accordingly, industrial perspectives proposed for AMTs led to considerable growth of up to US$ 18 billion in revenue by 2023 in the global AMT market (Figure 1a).^7^ Moreover, the compound annual growth rate for the AMT industry is estimated to be around 21% between 2022 and 2030.^9^ This perspective is driven by the fact that modern AMTs are capable of printing accurate parts with good surface finish at high throughputs and reasonable costs per part. Obtained material properties are equally important. Special thermomechanical properties (strength, stiffness, fracture toughness, heat deflection temperature) are in demand as well as appealing aesthetic and haptic properties and continuously satisfying long-term performance (e.g., no yellowing, no embrittlement).

A specific feature of most AMTs is that the material with its final properties is produced within the printer. Examples of this are the creation of specific microstructures during solidification in a selective laser melting process as well as the formation of a specific polymer morphology in a fused filament fabrication process. This strong correlation between the formation of a material’s morphology and processing is even more pronounced for AMTs based on photopolymerization reactions, commonly referred to as vat photopolymerization. In such techniques not only the shape but the material itself is made during the processing step. According to Wohler’s study, polymers in general account for the majority of the AMT market. Specifically, photopolymers have become a dominating proportion of 25% of all additively manufactured materials by the end of 2022 (Figure 1b).^7^

Vat photopolymerization techniques cover a variety of techniques with varying part-size ranges, printing speeds, and resolution (Figure 1c). Based on these versatile lithographic techniques, photopolymers and their use cases are continuously moving away from prototyping and small-scale series toward personalized products, e.g., personalized biosensors, protective gear for sports equipment, dental restorations, orthodontic retainers, as well as complex-shaped large-scale series parts, e.g., high-temperature resistant components of aircrafts or complex lattice structures.^13^ This leads to specific requirements in terms of structural and especially thermomechanical properties, as well as functional requirements such as recyclability, haptic, and optical properties.

Since the commercialization of stereolithography in the mid-1980s, 3D-printable photopolymer networks have mostly been based on densely cross-linked amorphous polymer network morphology, which limits attainable material properties such as fracture toughness and makes it difficult to achieve the performance of thermoplastic materials such as semicrystalline polyamides and polyolefins or heterogenized amorphous materials like the terpolymer acrylonitrile-butadiene-styrene (ABS). Along with the development of the underlying AM processes, photocurable resins are now starting to be redesigned to achieve additively manufactured photopolymers with mechanical properties competitive to conventional bulk polymers. Compared to thermoplastics such as ABS, thermoset photopolymers intended for AM exhibit notable desirable stiffnesses and heat deflection temperatures. However, historically, photopolymers have been developed for coating applications, which require sufficient hardness but tolerate brittleness comparably well. Therefore, their fracture toughness, defined as the resistance against fracture or crack propagation, requires much improvement for 3D parts as produced in AM. Appropriate toughening mechanisms are required to address this deficiency and expand the range of applications for such photopolymers in different industries. Purposeful heterogenization is one of the key toughening concepts in naturally evolved hierarchically structured materials such as bone, teeth, or wood, indicating the potency of this approach. Over time, the importance of heterogenization to alter the mechanical properties of materials has further been corroborated by its extensive use in the reinforcement of a variety of synthetic materials, of which polymerized nanocomposites and ABS are only two notable examples. A deeper understanding of these materials will aid the implementation of physically or chemically induced microstructure heterogeneity in photocurable systems, and it can be expected that future high-performance, 3D-printable photopolymers will be based on heterogeneous microstructures in analogy to thermoplastics.

Therefore, this Review presents the state of the art in the field of heterogeneously structured photopolymers by critically analyzing the success of various heterogenization approaches, from which a roadmap for improving photopolymer performance is then deduced. Since this area of research is still very young, photopolymer examples will be limited in some heterogenization approaches, and they will be supplemented with successful examples from the broader polymer context, after which photopolymers could be modeled.

## 2 Photopolymer-Based Additive Manufacturing

Currently, many different AMTs are available. The ASTM International Committee F42 on additive manufacturing technology defined a number of terms to distinguish additive manufacturing technologies and categorize the utilized approaches.^4,14^ In this Review, we follow this categorization and focus on the processes which have the largest relevance in terms of photopolymerization: vat photopolymerization and inkjet printing.

### 2.1 Vat Photopolymerization

Key elements of 3D-printers based on vat photopolymerization are a structured light source, a vat, containing the photopolymerizable formulation, and a build platform, which commonly moves perpendicular to the plane of the vat.

The prototype of such a stereolithography apparatus (SLA) was first developed by A. Herbert,^15^ although this setup was never commercialized. In 1984, patent applications for more advanced systems relying on UV-lasers to cure photopolymers layer by layer were filed by Charles Hull^16^ in the United States and by Jean-Claude André and co-workers^17^ in France. The activities in the United States led to the formation of 3D Systems, which has become one of the leading companies in the field of AMTs. The initial setup, which is still used nowadays by 3D Systems and competitors in a slightly modified form, uses a UV-laser in combination with a galvano-scanner to selectively expose the photocurable resin (laser SL). Exposure occurs from the top of the vat and onto a printing platform. After one layer has been cured, the emerging part is lowered by one layer height and a blade covers the top surface of the part with fresh resin. In an iterative way, layer after layer is exposed until the part is finished. The process has several advantages, particularly achievable feature resolution and surface quality. There are also some challenges associated with this method. Since the final part is completely immersed in the resin, the initial filling of the vat requires substantial amounts of photopolymer, especially when larger parts are printed. The recoating process with the blade limits the minimum layer height, and layer heights below 50 μm are difficult to achieve when using high-viscosity resins. The sequential nature of layer exposure also results in long printing times for large parts with high feature resolution.

To solve some of these challenges, alternative approaches have been proposed, with the majority of them being commercially available. Many of these approaches rely on light exposure from below, where the light source is positioned underneath a transparent vat, allowing the light to pass through the bottom of this vat before curing the resin. The build platform is lowered into the vat, so that the distance between the build platform (or the last printed layer) and the vat surface defines the layer height. This enables the creation of very thin and uniform layers, down to around 10 μm. As a drawback of this method, substantial peel-off forces can occur when the part sticking to the build platform detaches from the vat surface. Coating the glass vat with silicone and/or fluorinated polymers reduces these forces. In particular, low molecular weight components in the resin have the potential to diffuse into the vat surface, further increasing the detachment forces.

A solution to this problem was proposed by using permeable vat surfaces, which let oxygen diffuse through the vat into the resin, thus forming an inhibition layer when (meth)acrylate resins are used.^18^ The inhibited layer remains liquid, preventing the attachment of the part to the vat. In addition to reducing attachment forces, this approach allows significant increases in print speeds up to around 100 vertical mm per hour.

In the context of printing photopolymers with improved thermomechanical properties, high-viscosity resins play a crucial role. Toughening mechanisms frequently rely on high molecular weight oligomers and polymers, and on strong intermolecular interactions between the chains. This requires modified 3D-printers capable of processing such high-viscosity resins.^19^ A commonly adopted approach, implemented in several commercially available printers, is to increase the temperature in the processing zone to reduce the resin’s viscosity.^19^ Increasing the temperature can also enhance the reactivity of the resin and in further consequence improve the printing speed of the system. For example, less reactive ionic polymerization systems become sufficiently reactive at higher temperatures and offer new materials for light-based AMT.^21,22^ Overall, such approaches are instrumental in advancing the industrial applicability of photopolymer-based 3D-printing. They allow the use of previously unattainable photopolymers with improved thermomechanical properties and contribute to higher achievable throughput.

In terms of light exposure strategies, alternative concepts to laser SL have been proposed to compensate for some of their drawbacks. Using dynamic masks in combination with light-emitting diodes or high-pressure mercury lamps allows for exposure of a complete layer at once. Originally, such systems were developed for ceramic 3D-printing and microfabrication.^23^ Nowadays, dynamic masks based on micromirror devices (digital light projection, DLP) in combination with light-emitting diodes (LEDs) as the light source^24^ are the preferred printing approach for several reasons. The use of LEDs as the light source allows switching the light on and off between exposures, which improves energy efficiency and prevents unwanted illumination between exposures (e.g., during moving the build platform and replenishing resin in the exposure zone). DLP chips are used frequently in consumer electronics, making them readily available at reasonable cost. Compared to liquid crystal display (LCD) masks, DLP chips offer a better contrast ratio, leading to more defined contours in the final part due to the reduced dark-field intensity. DLP SL exposes each layer in one shot, increasing printing speed compared to laser SL.

### 2.2 Inkjet Printing

In the context of heterogeneous photopolymers for AM, inkjet printing is a highly interesting technique.^25,26^ The availability of multimaterial print heads with thousands of nozzles can substantially simplify the manufacturing of multiphase materials, sometimes referred to as “digital materials”.

Currently, two basic principles are used for depositing droplets onto the substrate, namely continuous inkjet printing and drop-on-demand (DOD) techniques. For applications in AM, DOD techniques are preferred, where the individual droplets are created by generating an actuation pressure within an ink channel, either by piezo acoustic actuators or by thermally generating small gas bubbles. Considering the achievable frequency of droplet formation in the range of 10–100 kHz and a typical droplet volume in the range of 0.5 to 100 pL, substantial throughputs can be achieved when inkjet heads with hundreds or even thousands of nozzles are employed.

#### 2.2.1 Rheological Parameters

A helpful tool in fluid dynamics is the use of dimensionless numbers, which express the ratio of the various forces (or time- or length scales) acting within the fluid. In the realm of inkjet printing, the Reynolds number *Re*, Weber Number *We* and Ohnesorge number *Oh* are most relevant. *Re* characterizes the balance between inertial forces (i.e., fluid density ρ, velocity *v*, and characteristic length (or diameter) *d*) and the viscosity η:
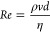
1

In a similar way, *We* is related to the inertial forces versus surface tension γ. In the case of inkjet printing, γ is related to the capillary forces:
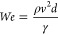
2

These two factors can be combined to form a dimensionless variable, called the Ohnesorge number *Oh*, the ratio between the viscous and capillary time scales:
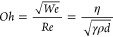
3

#### 2.2.2 Processing Window for Inks

**Figure 2:**
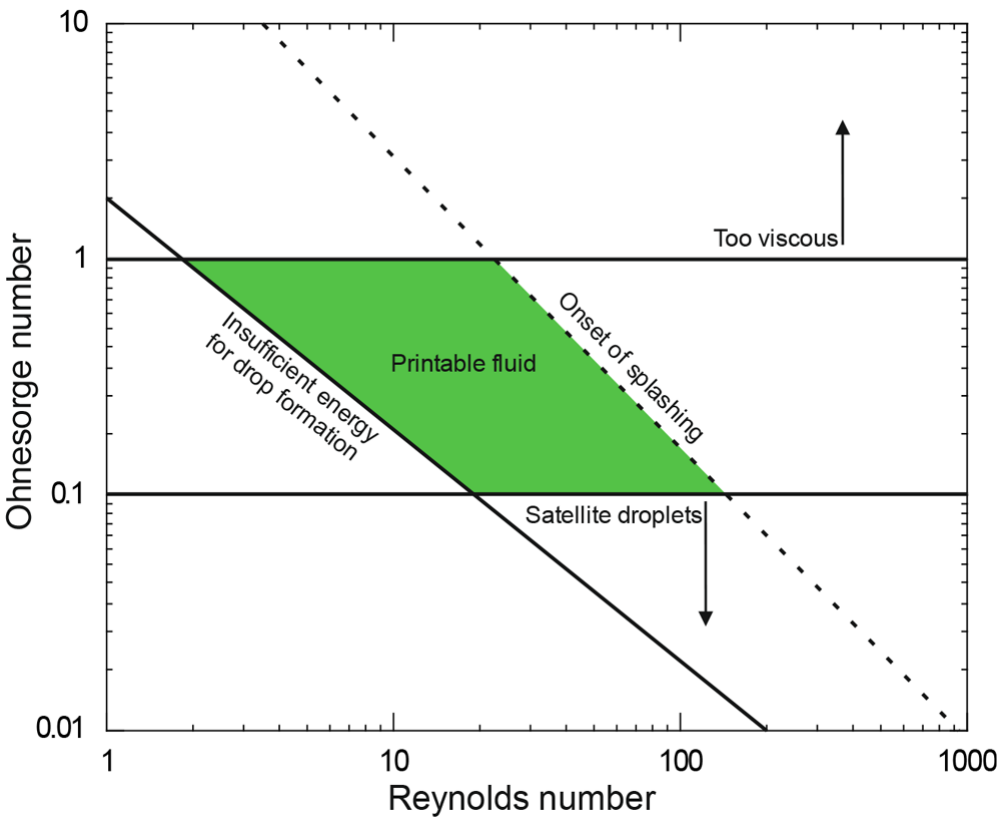
Schematic diagram of processing window (green area) for inkjet printing. Regions outside this area are not accessible to inkjet printing due to reasons such as high ink viscosity, and thus inability to jet through nozzles, or the formation of satellite droplets during jetting due to insufficient surface energy. Alternatively, splashing or drop formation may occur due to insufficient kinetic energy needed for ejecting droplets from the nozzle.^26^

The processing window accessible for ink development is given by the dimensionless numbers above (Figure 2). In AMT applications using photopolymerizable ink formulations and industrial inkjet heads, typical material and nozzle properties can be estimated: A realistic nozzle diameter is around 45 μm, the density of the fluid is 1000 kg m^–^^3^ and the surface tension is 35 mN m^–1^. The allowed upper limit, *Oh* = 1, leads to a maximum viscosity of 40 mPa s^–1^, which is one of the key limiting factors for developing inks. As described previously, large intermolecular interactions between polymer chains due to strong secondary bonds will yield better thermomechanical properties. These strong secondary bonds, on the other hand, will lead to increased viscosity in the ink to be jetted. This is also valid when inks are filled with organic or inorganic particles to promote heterogenization. One approach to overcome these limitations and at the same time use the potential of inkjet technology for digitally modifying the microstructure of 3D-printed materials is the use of hybrid processes.^26^

## 3 Chemical Toolbox

### 3.1 Resin Components

**Figure 3:**
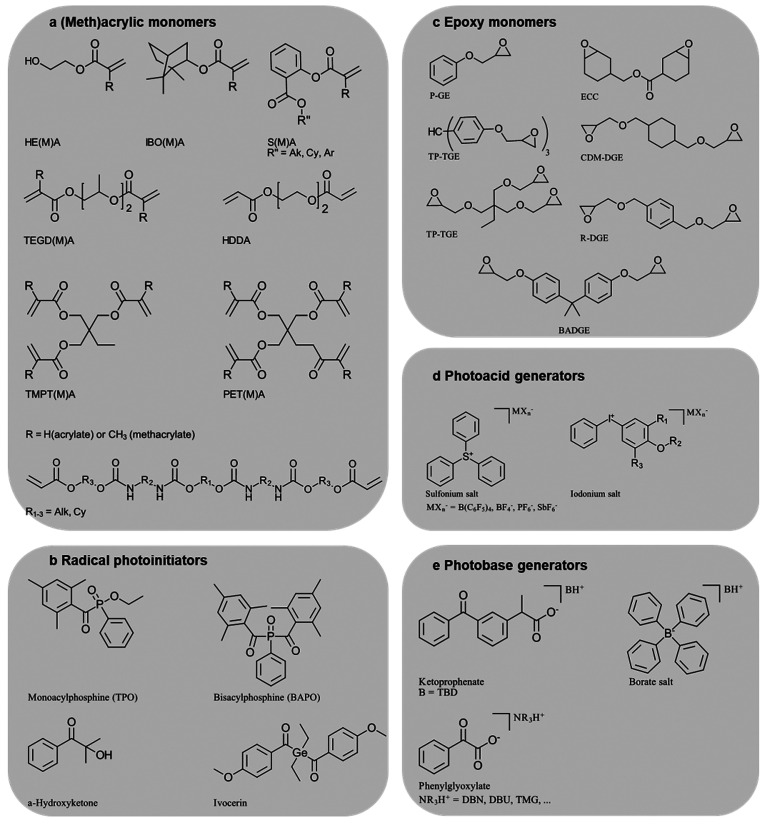
(a) Common (commercial) acrylate (R = H) and methacrylate (R = CH_3_) monomers^44^ and (b) corresponding radical photoinitiators as well as (c) common epoxy monomers, (d) corresponding photoacid generators, and (e) photobase generators for vat photopolymerization.

In their most fundamental form, photopolymers consist of mono- and multifunctional monomers. While small molecular weight mono- and multifunctional building blocks allow the resin to be liquid under ambient conditions, high molecular weight multifunctional monomers are necessary to obtain form stable specimens and the desired mechanical properties. In radical photopolymerization, most commonly, acrylates and methacrylates are used, of which standard vat photopolymerization monomers are depicted in Figure 3a.^28^ Another commonly used monomer type is epoxides, which are polymerized via a cationic mechanism (see examples for commercial monomers in Figure 3c).^28^ Traditionally, such cationic polymerizations were conducted in parallel with radical photopolymerizations during 3D-printing to obtain sufficient reactivity for AM. Most recently, however, ionic photopolymerization at elevated temperatures has been explored by our group, which exhibits sufficient reactivity for vat photopolymerization.^29−31^

Depending on the polymerization mechanism, radical initiators or photoacid/photobase generators, which release super acids or bases upon irradiation, can be used (Figure 3b, d, e).^32,33^ To adapt the initiator’s responsivity to the light source, in particular for cationic systems, sensitizers like isopropyl thioxanthone or dibutyl anthracene may be utilized. Furthermore, the addition of light absorbers may be necessary to maintain high spatial resolution during the printing process and avoid so-called overpolymerization due to the migration of the reactive components out of the illuminated area. In the case of photoacids, amines are used to avoid overpolymerization due to the migration of the photoacid upon its activation. Additionally, inert fillers may be added to the formulation to improve the thermomechanical performance. Depending on their nature, they may intervene with the curing depth and the resin’s photosensitivity.^34^

The cross-linking density is directly dependent on the ratio of mono- to multifunctional monomers and vastly influences the mechanical performance. This also applies to the chemical structure of the monomer. (Multi)functional polymerizable oligomers, as intermediate molecular weight molecules, have been developed as monomers for radical photopolymerization to deliver specific qualities such as improved hardness, abrasion, flexibility, pigment wetting, chemical resistance, and toughness to the resulting photopolymers. For example, acrylate-terminated urethane oligomers are relevant toughness-modifying oligomers (Figure 3a, bottom structure).^20,35^ The lengthy aliphatic backbone improves the material’s fracture resistance and makes the network more flexible at service temperature. In another example, the effect of the molecular weight of a poly(caprolactone)-based dimethacrylate has been studied in detail for potential application in the biomedical field.^36^ High molecular weight polyurethane-based reactive oligomers have been claimed to significantly increase the toughness of classical photopolymers.^35,37−40^ Such systems could have molecular weights up to 10 kDa.^41^ Hyperbranched polymers are beneficial as well, as they give materials with outstanding HDTs.^41^ Poly(aryl ether sulfones) with at least one polymerizable group and a molecular weight of larger than 12 kDa have also been claimed to give materials with superior mechanical properties.^42^ However, their commonly associated high viscosity renders processing via AM challenging, which needs to be addressed through the use of reactive diluents and higher temperatures.

Reactive diluents are usually small molecular (meth)acrylic compounds, which lower the resin viscosity (Figure 3a).^43,44^ Monofunctional reactive diluents, such as hydroxyethyl methacrylate (HEMA) and isobornyl (meth)acrylate (IBO(M)A), contribute to reducing the overall cross-linking density, whereas multifunctional and sometimes hyperbranched reactive diluents such as 1,6-hexanediol diacrylate (HDDA), trimethylolpropane tri(meth)acrylate (TMPT(M)A) and pentaerythritol tetra(meth)acrylate (PET(M)A) show the opposite effect. Formulation composition changes to improve processability, however, need to be approached cautiously, given that the high content of reactive diluents significantly impacts the thermodynamics and kinetics of the polymerization as well as the material properties. Furthermore, these compounds cannot always lower the viscosity sufficiently for AMTs (11–13 cP at 70–80 °C for material jetting and around 10 Pa s at printing temperature for SL-based techniques). Hence, new monomers that contribute to dilution without sacrificing ultimate material properties, in particular shrinkage, are necessary to overcome the issue.^45^ For example, triethylene glycol dimethacrylate (TEGDMA) may increase the filler capacity of the compound significantly,^83^ and cyclopolymerizable monomers (CPMs) create bulky units in the backbone and thus effectively reduce volume shrinkage.^11,46,47^ Furthermore, CPMs are, along with salicylate-derived monofunctional (meth)acrylates,^48^ a viable option to address the problem of volatility for photopolymerizations at elevated temperatures.^4,45,49,50^ Ionically, induced ring-opening photopolymerizations are another highly efficient tool in this regard. While epoxides have been utilized to reduce shrinkage for a while already, cyclic oxazolines,^100^ esters^29,1000^ and carbonates^30,1001^ have only recently been introduced as versatile monomers for additive manufacturing. To accommodate the call for more sustainable monomers, renewable raw materials such as vegetable oil and lignin derivatives have been utilized for monomer synthesis, mainly by utilizing unsaturated backbones, (meth)acrylation, or epoxidation to incorporate photopolymerizable functional groups into the molecule.^51,52^

### 3.2 Curing Conditions

The formulations must be liquid and maintain low viscosities at the processing temperature during vat photopolymerization. Therefore, in addition to reactive diluents, variation of the processing temperature is an excellent tool to make highly viscous formulations applicable for vat photopolymerization. Elevated temperatures, particularly in cationic polymerization, accelerate the curing reactions, enabling the formulation to reach sufficient conversion of the reactive monomers for swift gelation. Form-stable solid specimens can thus be obtained faster, shortening the printing process. This makes reactions available for vat photopolymerization, which have previously been too slow to obtain green parts with feasible printing speeds.^53^

Additionally, the light source, either mono- (laser) or polychromatic (LED), needs to be compatible with the initiator in the formulation. Alternatively, sensitizers could be introduced to the formulation to create an overlap between the light source emission and initiator absorption.^33^ When utilizing two types of initiators (e.g., radical and cationic) with distinct initiation wavelengths, it gives rise to orthogonal curing of monomers in the resist.^54,55^ There are also emerging noncatalyzed light-triggered reactions for multicolor reactivity purposes.^56,57^

Traditional vat systems (SL, DLP-printing) rely on single-photon absorption to initiate photopolymerization. More recently, two-photon absorption has been discovered as a means for lowering resolution thresholds and producing micrometer-scale parts (multiphoton polymerization). This method allows for writing structures freely within the formulation, at the point where the laser focus reaches sufficient intensities to deliver photons quickly enough to one molecule.^58,59^ Thus, femtosecond lasers are employed as the light source in this process.^60^

On the whole, photopolymerization processes are associated with a reduction in overall free volume known as shrinkage because the van der Waals forces and/or hydrogen bonds between monomer/oligomer building blocks (0.3–0.4 nm) are replaced by shorter covalent connections (∼0.154 nm).^61^ Compared to the polymerization of monofunctional monomers, much higher shrinkage can occur during cross-linking of multifunctional monomers (up to 23 cm^3^ mol^–1^ for multifunctional methacrylates).^62^ High degrees of shrinkage may cause internal stress within the network as well as between the shrinking layers and therefore may lead to geometry deviation from CAD data, anisotropy, and deformation of parts,^63−65^ or enhance the probability of failure by fracture.^61,66^

**Figure 4:**
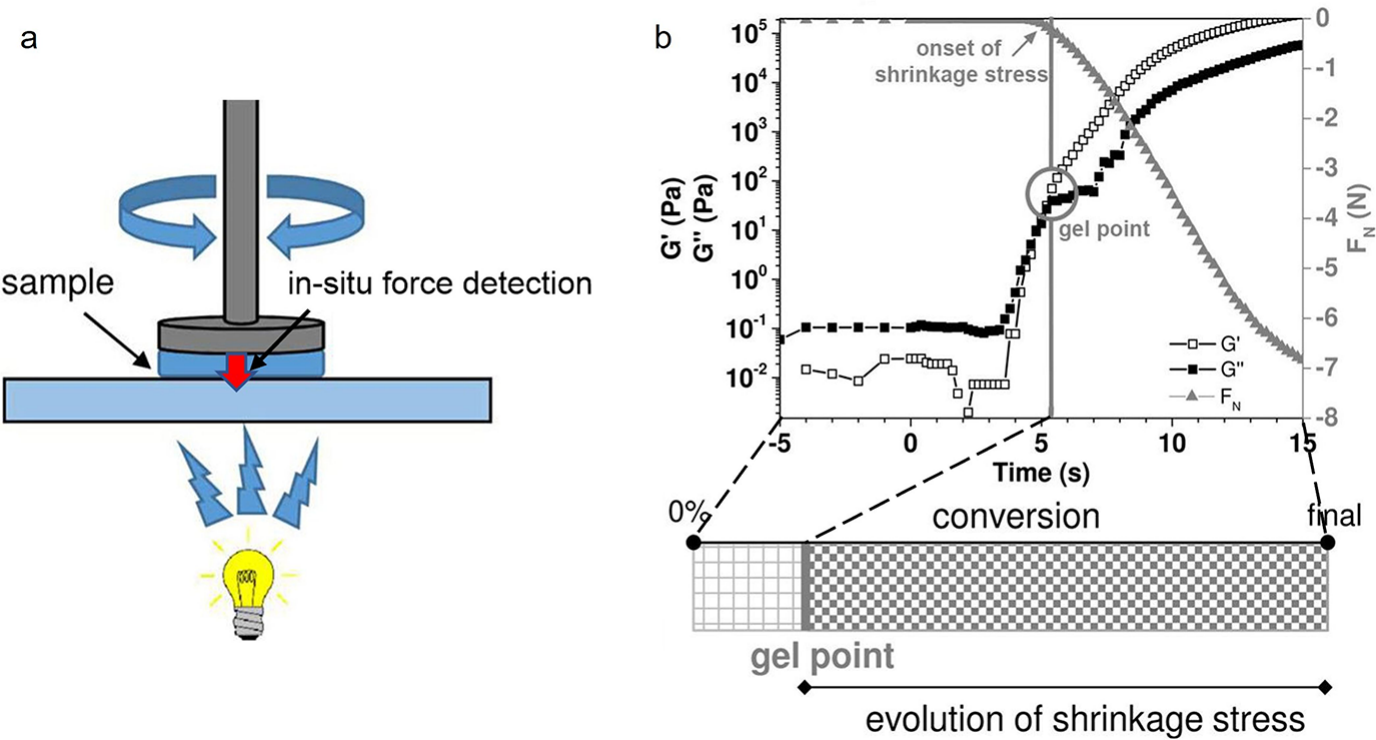
(a) Schematic visualization of in situ force detection during photorheology and (b) photorheologically determined storage and loss modulus curves indicating the onset of shrinkage stress, which correlates with the in situ determination of forces developing between the measurement plates. Adapted with permission from ref (68). Copyright 2017 American Chemical Society.

The most suitable parameters to determine shrinkage behavior and associated stress evolution are the rate of reactive group consumption as well as the onset of the gel point. As pointed out previously, fast chain growth, particularly in radical photopolymerization, leads to vitrification at low conversions. This prevents the shrinkage stresses from being released effectively, and therefore, the potential of such polymers to eventually break by fracture is high. Many shrinkage measurement systems have been proposed over the years, e.g., dilatometry, linometry, optical measurements, and rheology.^67^ In this context, RT-NIR photorheometry is particularly noteworthy as it can monitor several parameters simultaneously and in situ during the photocuring process: the double bond conversion, the viscoelastic behavior (storage and loss modulus, with the gel point at their intersection), and the evolution of the normal force (Figure 4).^68^ The point during photopolymerization when shrinkage stress occurs corresponds well with the times until the gel point is reached, underlining the importance of gel point determination and its delay toward higher double bond conversions, which allows for the rearrangement and relaxation of the forming network to reduce structural irregularities and shrinkage stress.^68^ The shrinkage level in such cross-linked polymers can be mitigated by properly adjusting the polymerization kinetics. Slowing down the network formation and thus delaying the gel point leads to more homogeneous networks. Delayed gelation can be achieved by adjusting processing conditions, such as irradiation intensity and temperature.^69^ Moreover, various formulation refinements, such as reducing the photoinitiator content, employing less reactive monomers, incorporating inhibitors and retarders, and particularly chain regulation are challenging but effective strategies.^70^ Another example is the use of recoverable bonds. Photopolymer systems including reversible (recoverable) covalent bonds at the matrix–silica interface have been investigated for this purpose.^71^ Furthermore, polymerizations in which the volume change between the monomer unit and the chain unit is counteracted by steric effects can be employed. Good examples are cyclopolymerizable monomers that form cyclic units in the polymer backbone^11,29,46,47,72^ or ring-opening monomers reduce volumetric shrinkage.^29,73−77^

### 3.3 Chain Regulation

The fast chain propagation in free radical polymerization, for example in (meth)acrylate resins, followed by gelation at low conversions (about 20% for methacrylates) renders network formation into a diffusion-controlled mechanism and results in a broad distribution of cross-linking densities and therefore a brittle and inhomogeneous network architecture. This can be moderated through chain transfer reactions where a propagating radical reacts with a nonradical chain.^78^ While such reactions may occur between all present reaction partners in a photopolymerizable formulation, they can also be provoked purposefully through the addition of chain transfer agents (CTAs), for which the reinitiation step after the chain transfer is faster than the propagation step. Since this Review will only introduce this area of research briefly as a tool that can be utilized in photopolymer heterogenization, interested readers should additionally refer to an excellent in-depth review of those concepts by Fang and Guymon.^50^

**Figure 5:**
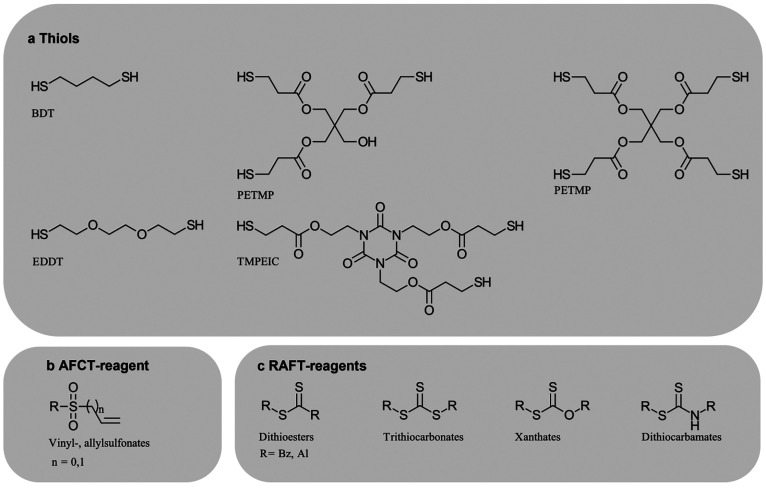
(a) Common thiol molecules, (b) addition–fragmentation transfer (AFCT) reagents, and (c) reversible addition–fragmentation transfer (RAFT) reagents.

More homogeneous polymer networks can be achieved by switching from radical chain growth to step growth polymerization via thiol-mediated chain transfers (Figure 5a). Pioneering work in that field has been performed by Charles Hoyle and Christopher Bowman in the early 2000s with thiol-ene polymerization.^79^ Such more controlled radical photopolymerization techniques have recently gained significance for vat photopolymerization. Chain transfer constants in thiol-ene polymerization are between 0.3 and 0.5 for (meth)acrylates.^80^ Therefore, a significant amount of chain growth is still present.

Currently, thiol-ene systems offer some of the highest toughness properties among photocurable systems. This improvement comes at the expense of stress resistance and modulus, in particular at high temperatures. This is usually due to the high flexibility of thio-ether bridges at high temperatures. Moreover, storage stability and potential homopolymerization, especially in the case of acrylates, is problematic.^81^ The bad odor of thiol monomers limits practical application because nearly all commercially available thiols are based on thioacetic or thiopropionic acid, which can cleave hydrolytically over time. Thiol-ene polymerization is arguably the most explored controlled photopolymerization technique applied in vat photopolymerization.^82−85^

Thiols are seen as the earliest example of the chain transfer concept, which has been diversified since. Depending on the chemical structure of the reagent, chain transfer reactions are irreversible (addition–fragmentation chain transfer, AFCT, Figure 5b) or reversible (reversible addition–fragmentation transfer photopolymerization, RAFT, Figure 5c).

The use of AFCT agents to homogenize the photopolymerized networks has been shown to increase toughness.^86,87^ AFCTs such as allylsulfonates^88^ and vinyl sulfonates^89^ have been proven to produce more homogeneous networks without the loss of mechanical strength.^86,90^ In the case of vinyl sulfonates, a chain transfer constant close to 1 and minimal delay in polymerization has been observed, which is important for reasonable printing speeds.^91^ Indeed, the vinyl sulfonate CTA could be applied as a nonmigrating CTA in hot lithography.^87^

RAFT has been utilized in AMT as well. The polymerization kinetics and the thermomechanical properties of a urethane methacrylate system in the presence of RAFT reagents have been studied in detail.^92^ The photopolymerization rate and Young’s modulus decrease but the elongation at break is significantly improved. In other examples, macroRAFT reagents have been utilized for similar purposes.^93−96^ The mechanical properties can be modified within a broad range, the printing resolution was reported to be increased compared to free radical vat photopolymerization of comparable rates, and phase separation can be achieved through a clever choice of reagents. Furthermore, photo-iniferters have been explored in the field of radical/cationic hybrid systems, giving block copolymers with improved mechanical properties.^97^ While CTA-regulated networks generally report improved mechanical behavior in terms of toughness (at room temperature), this is often accompanied by a drop in their glass transition temperature due to the reduced cross-linking density.

## 4 Characterization Toolbox

**Figure 6:**
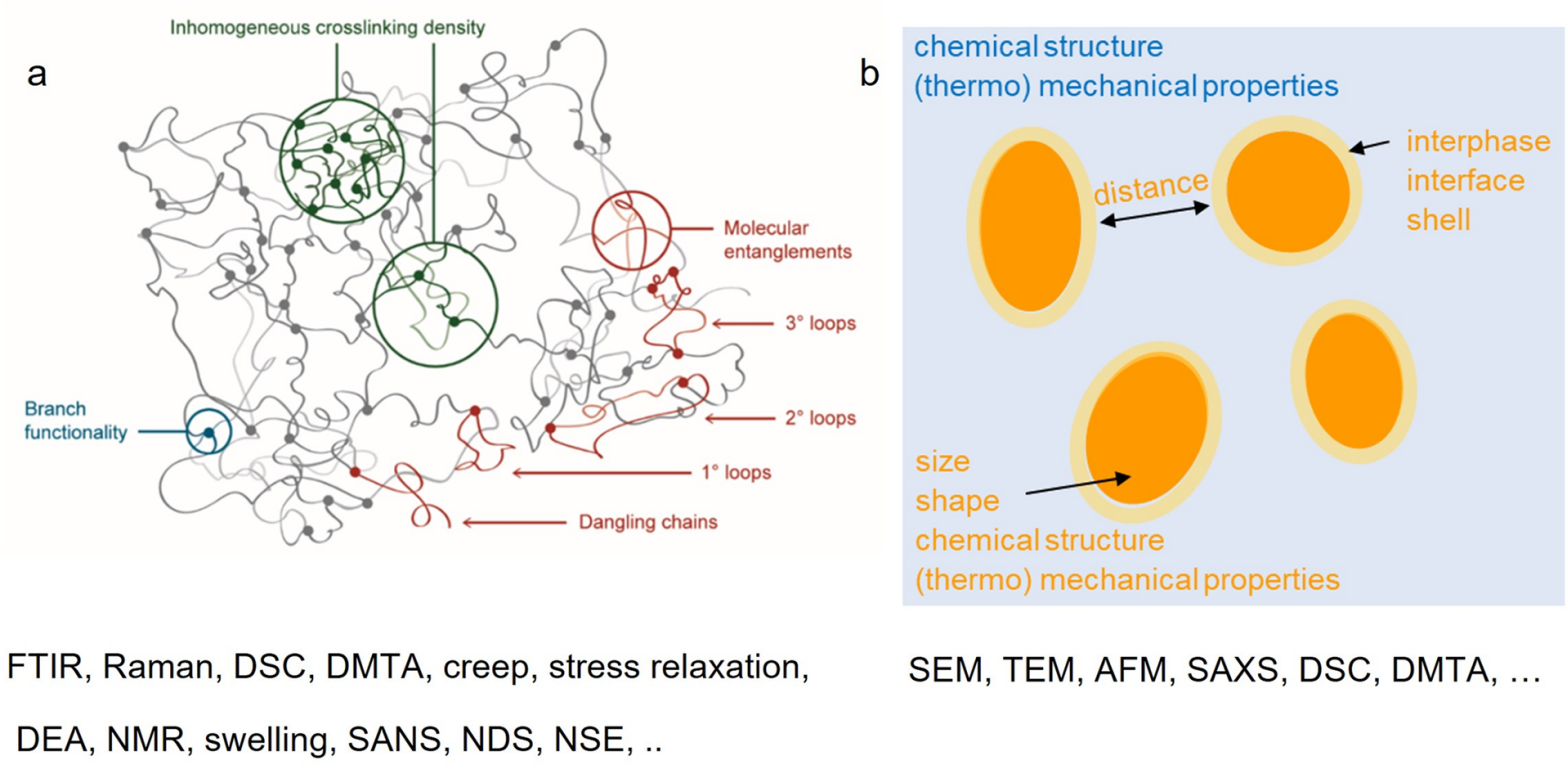
(a) Complex topology of a network structure. Reprinted with permission from ref (98). Copyright 2019 Elsevier. (b) Factors affecting the toughness behavior of a phase-separated system. Additionally, Fourier transform infrared spectroscopy (FTIR), Raman spectroscopy, differential scanning calorimetry (DSC), dynamic mechanical thermal analysis (DMTA), creep, stress relaxation, data envelopment analysis (DEA), nuclear magnetic resonance (NMR), swelling, small-angle neutron scattering (SANS), network disassembly spectrometry (NDS), neutron spin echo (NSE), scanning electron microscopy (SEM), transmission electron microscopy (TEM), atomic force microscopy (AFM), small-angle X-ray scattering (SAXS), and differential scanning calorimetry (DSC) as the relevant characterization methods are exemplified.

The characterization of photopolymers is generally challenging because the structure and morphology of the materials are complex and span over different length scales. Looking at the topology of network structures, three relevant length scales exist: 10–100 nm, topology covers inhomogeneity in the local distribution of cross-linking junctions; 1–10 nm, topology covers structures on the macromolecular level and can include parts of one or multiple polymer chains; <1 nm, molecular level structures with less than ten chemical bonds.^98^ Detailed descriptions of applicable characterization methods for network structures are readily available in the literature.^98−100^ Focusing on heterogeneities of larger dimensions, especially on systems undergoing phase separation, it is important to consider the parameters defining such morphologies (Figure 6). In the following, common characterization methods are summarized, specifically focusing on the characterization of heterogenization and heterogenous microstructures.

### 4.1 Bulk Characterization

#### 4.1.1 Turbidity

Similar to the blend of immiscible transparent thermoplastic polymers, which becomes opaque upon mixing, thermodynamic phase immiscibility in thermosets may deliver different optical properties compared to glassy thermosets or their initially transparent multifunctional monomer formulations that undergo in situ cross-linking.^101,102^ The resulting photopolymer may be transparent, translucent, or opaque depending on (i) the contrast in the refractive index of compositionally different phases and (ii) the dimension of the chemical or physical light scattering phases. According to the Rayleigh scattering theory, heterogeneities (structures of particles) larger than the wavelength of light can scatter light. In addition to light scattering techniques (e.g., haze measurement) that provide data on optical properties, spectroscopy techniques can be employed to estimate the heterogeneity size and distribution. Such techniques can also help monitor the onset and progress of phase separation in photosensitive materials.^103−105^ Combining spectroscopic techniques with controlled photocuring systems, measuring, and studying the conversion of monomers during the phase-separation process also becomes possible.^101,106,107^

**Figure 7:**
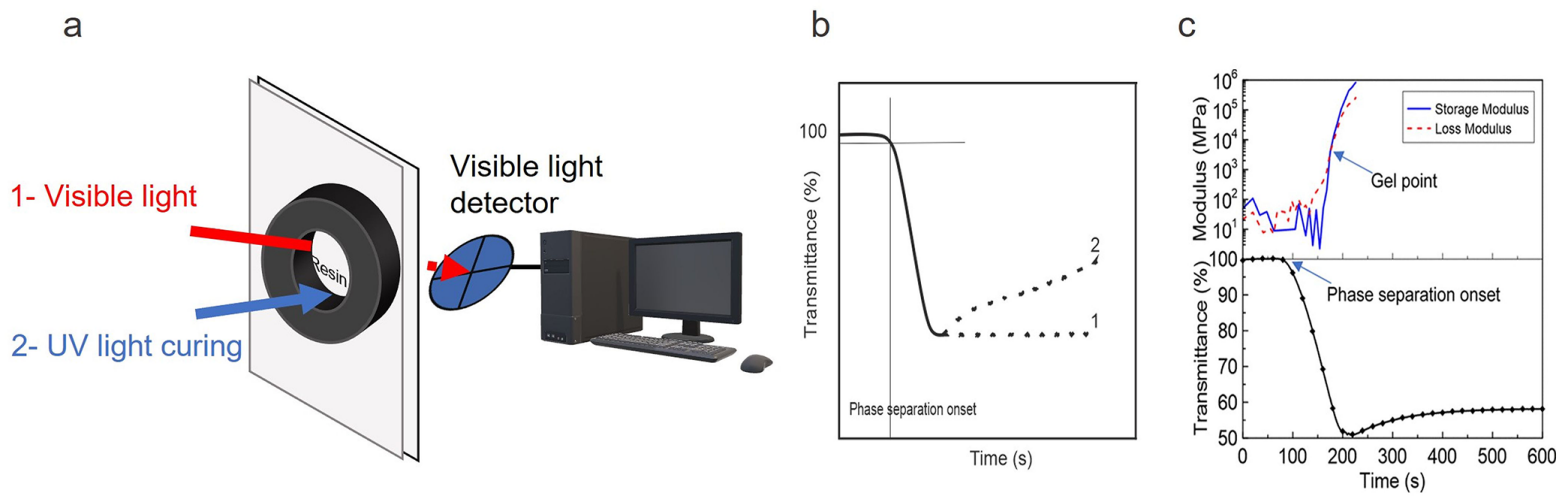
(a) Simple illustration of measuring the onset of PhIPS. (b) Typical pathway of transmittance over irradiation time for phase-separable formulations. (c) Example of concurrent measurement of the onsets of PhIPS and gel point. Reprinted with permission from ref (111). Copyright 2019 American Chemical Society.

To measure the phase-separation onset, changes in the optical density of the curing sample need to be tracked. The spectrometer thus records the quantity of noninteracting visible light that passes the sample continuously and reaches the detector (Figure 7a). The initial receiving voltage corresponding to the sample’s full relative transparency declines over time as the formed heterogeneities grow large enough to scatter incoming visible light. The general pattern of subsequent turbidity evolution (transmittance drop) over time for phase-separable mixtures is shown in Figure 7b. Different monomer reactivities, polymerization conditions (irradiation intensity, temperature, oxygen exposure, etc.), and varying local monomer conversion may affect this pattern.^104,108,109^ The final deviation from 100% transmittance is perceived as the upper limit of onset since phase separation may emerge at size scales that are not detectable by the spectrometer, especially when the phase separation follows the nucleation and growth mechanism.^110^ The study of Hasa et al. on an IPN system that consists of butyl acrylate and difunctional oxetane suggests a close overlap between the maximum transmittance drop time and the gel point (Figure 7c).^111^ Following gelation, the maximum turbidity may exhibit two distinct patterns including “modest remaining” (1) or “partial recovery” (2), as illustrated in Figure 7b. Such recoveries are typically caused by the subsequent formation of a less reactive phase, which reduces the number or size of light-scattering heterogeneities and compensates for the difference in refractive index. For highly viscous compositions, such turbidity recoveries are thus less expected as the high viscosity directly affects this kinetic-related phenomenon.^112^ Notably, the homopolymerization of a multifunctional monomer may also result in a minor drop in transparency as the highly cross-linked microgels have a slightly different refractive index than the nearby unreacted monomers.^112^ The same might happen during the copolymerization of multifunctional monomers. Therefore, additional heterogeneity characterization techniques will be useful to fully characterize the phase separation.

#### 4.1.2 Dynamic Mechanical Analysis

**Figure 8:**
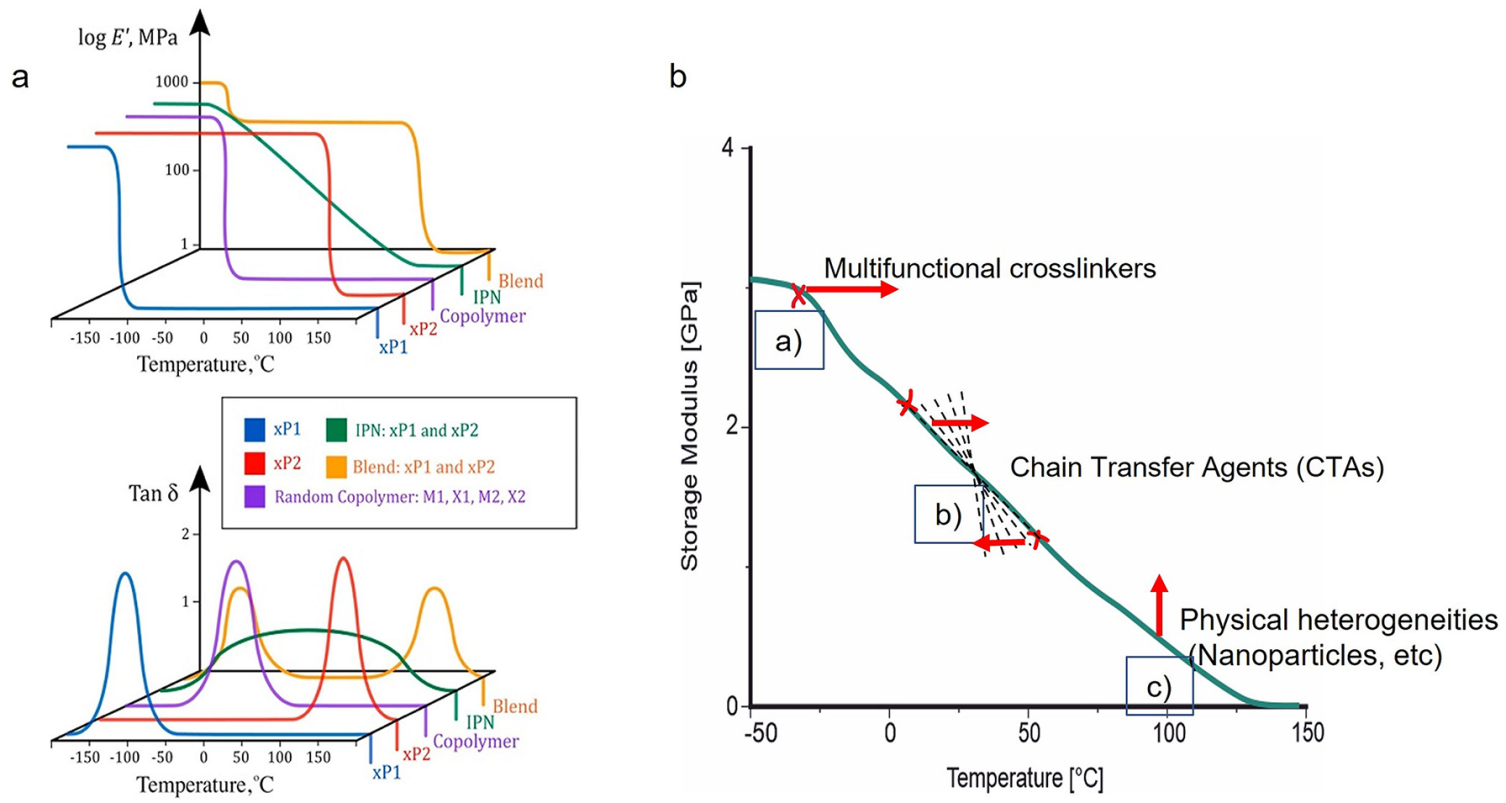
(a) Different composites derived from two different cross-linked (x) polymers (P), xP1 and xP2, characterized by DMA. M and x represent monomers and cross-linkers, respectively. Adapted with permission from ref (113). Copyright 2020 Elsevier. (b) Possible effects of the addition of cross-linkers (a), CTAs (b), and physical heterogeneities (c) on the presumed DMA analysis of a photopolymer.

Dynamic mechanical analysis (DMA) is a very powerful tool for the characterization of photopolymers in general and for their heterogeneous microstructure specifically. Various multicomponent polymers, including copolymers, polymer blends, and IPNs, exhibit distinct thermomechanical properties (Figure 8a).

The temperature-dependence of the storage modulus E′ (or G′) gives a good indication of the stiffness behavior upon application of the materials and the tan delta curve obtained by DMA reflects the likelihood of polymeric segments reacting uniformly to stresses and thus represents the structural architecture of polymers. Distinct peaks or shoulders of tan delta reflect motional transitions of chain segments (for example, the glass transition as the most important one). In common photopolymers, particularly the radically initiated ones, different cross-linking densities result in distinct domains that behave differently when relaxing the applied stresses. This diversity causes a broad transition and therefore makes it challenging to determine the glass transition temperature (*T*_g_). The full width at half-maximum (FWHM) has become widely agreed upon as a measure for the glass transition, as well as a representative of structural irregularities in materials showing single tan delta peaks.

**Figure 9:**
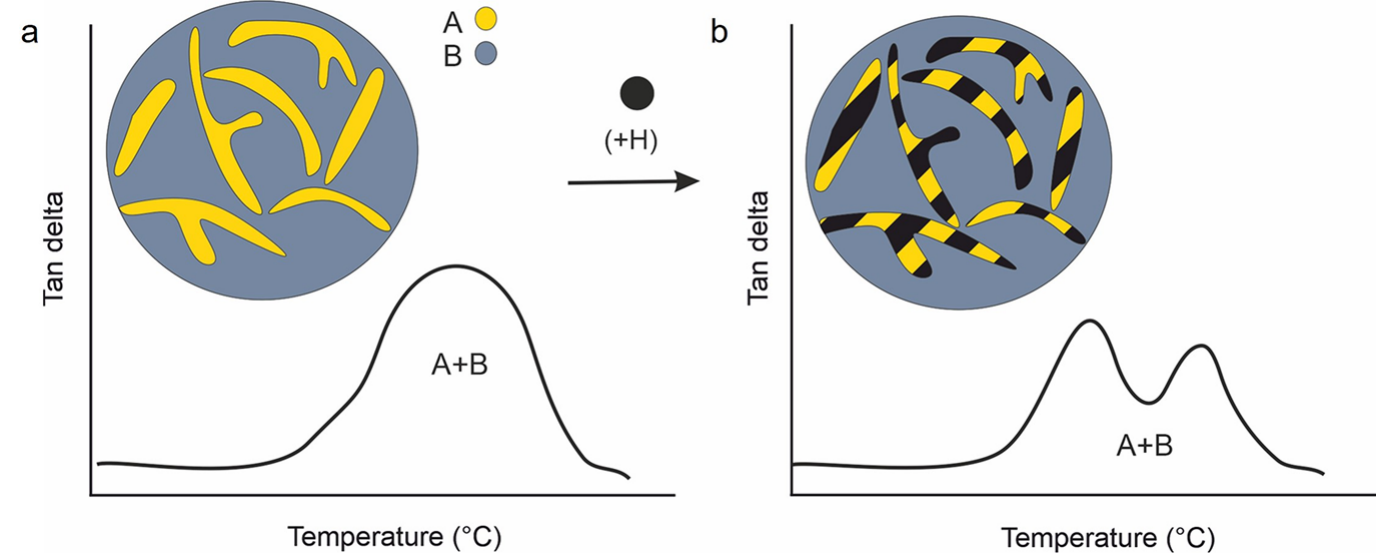
(a) Schematic diagram of a tan delta curve in the glass transition region of a phase-separated AB photopolymer if both phases have similar glass transitions and (b) distinct phase separation as the result of increased interaction of a physical heterogeneity (H) with one of the separated phases A or B (here: A).

A broadened FWHM can stem from various factors such as varying cross-linking densities within the material, partial phase separation, or even highly phase-separated components if their respective glass transition temperatures are very close to each other (Figure 9a). The peak’s position and magnitude predictably change with the composition ratio. Increasing the incompatibility between components, particularly those with different glass transition temperatures), however, tends to separate the peaks further and result in detached apexes for each phase. Such a clear separation can be promoted when a physical or chemical heterogeneity (nanoparticle, thermoplastic prepolymer, comonomer, etc.) has a higher propensity to one of the phases and gives rise to a higher contrast in the phase morphologies (Figure 9b). Higher contrasts also facilitate the characterization by other techniques such as microscopy.^109,114^

#### 4.1.3 Differential Scanning Calorimetry

**Figure 10:**
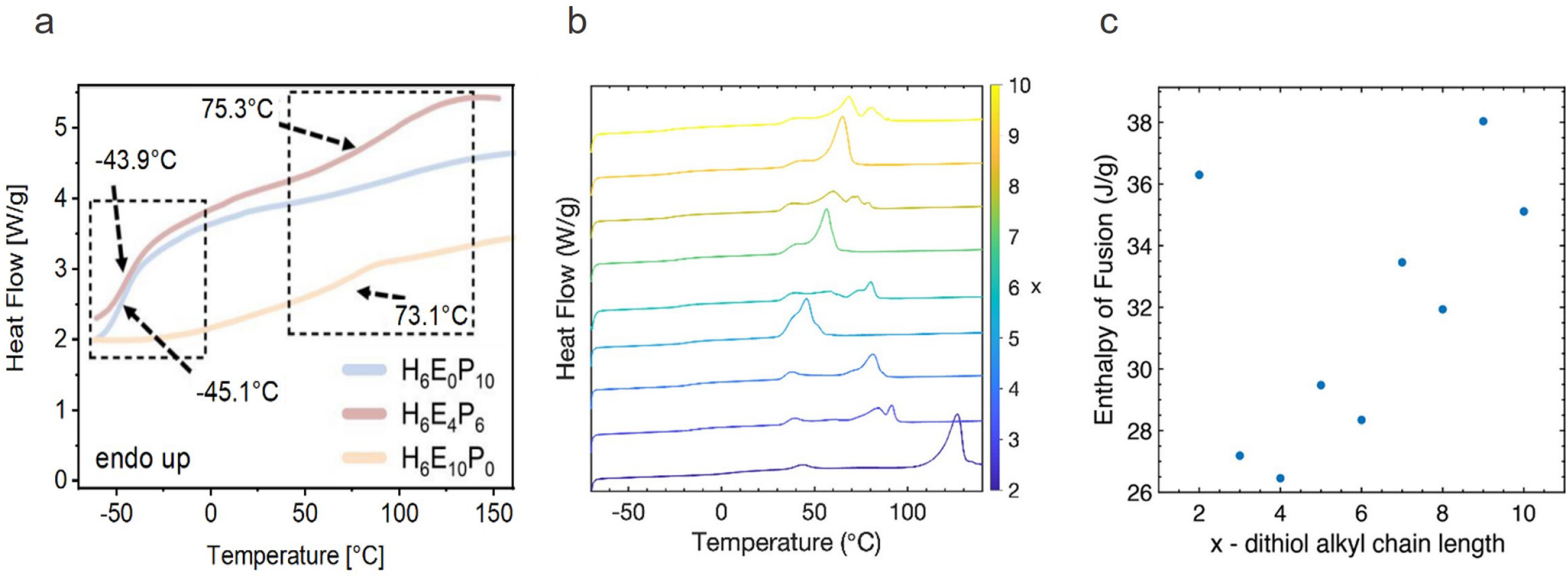
(a) DSC heating runs of 3D-printable gels consisting of different ratios of hydroxyethyl methacrylate (H), poly(ethylene glycol) (E), and poly(propylene glycol) (P) showing two distinct glass transitions in the case of the good separation of the H_6_E_4_P_6_ phase adapted from ref (115). (b) Melting behavior of semicrystalline dithiol–diallyl terephthalate (xDT-DAT) photopolymers obtained from thiol-ene polymerization and (c) dependence of enthalpy of fusion on dithiol alkyl chain length in the xDT-DAT system. Reprinted with permission from ref (116). Copyright 2021 American Chemical Society.

Differential scanning calorimetry (DSC) can be used to characterize the material after polymerization. Typical material parameters or states like glass transition temperature, degree of conversion, heterogeneity, phase separation, liquid-crystalline transitions or melting temperature, and crystallinity can be detected. Examplary cases are shown for a phase-separated amorphous system (Figure 10a) and semicrystalline photopolymers are shown in Figure 10b,c.

#### 4.1.4 Fourier Transform Infrared Spectroscopy

Fourier transform infrared spectroscopy (FTIR) is one of the most widely used analytical methods in polymer science in general and photopolymer analysis specifically. It can be used to determine the general molecular structure, especially in the midwavelength infrared region (MIR, 2.5–25 μm, 4000–400 cm^–1^), the so-called fingerprint region. Other useful type of information obtainable by FTIR are the reaction kinetics and the degree of polymerization, i.e., conversion by considering the change of material-specific characteristic absorption bands (A) and utilizing the changes in amplitudes or areas. Although FTIR is currently mainly used for bulk characterization of photopolymers and photopolymerization processes, newer highly spatially resolved nanospectroscopic methods like infrared scattering-type scanning near-field optical microscopy (IR s-SNOM),^117^ atomic force microscopy infrared (AFM-IR),^118,119^ and photoinduced force microscopy (PiFM)^120,121^ should become increasingly important in the future. With a spatial resolution down to 10 nm, they are predestined to investigate heterogeneous morphologies in photopolymers.

#### 4.1.5 Small-Angle X-ray Scattering

If the spatial resolution of heterogeneities is in the range of several angstroms to several tens of nanometers with varying electron densities between the regions (phases), small-angle X-ray scattering is suitable for phase-separation analysis. Typically, the scattered intensity or corrected intensity scattered at the angle 2θ and the wavelength λ is plotted as a function of the scattering vector *q*:
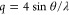
4

**Figure 11:**
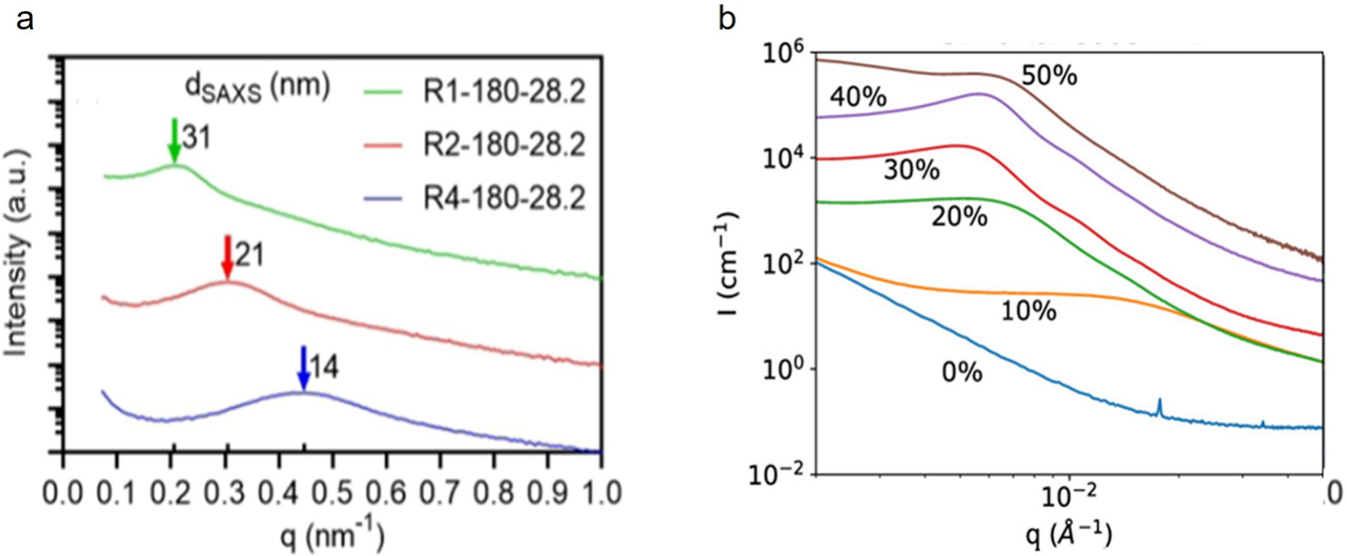
Examples of SAXS measurements of phase-separated photopolymers. (a) Polymerization-induced microphase separation in a PBA/P(AA-*stat*-PEGDA) system controlled by different PBA macroCTAs (1-arm (R1), 2-arm (R2), 4-arm (R4)), 28.2 wt% macroCTA each. Resulting morphology types: bicontinuous (R1), phase-inverted (R2), and bicontinuous/phase-inverted (R4). The annotations at the arrows give the calculated domain sizes in nm.^123^ (b) UV cured PDMS/PMMA–graft-IPNs with different PMMA contents (vol%, shifted curves for clarity). Adapted with permission from ref (124). Copyright 2020 American Chemical Society.

In general, the scattering intensity, *I* (*q*), cannot be used to deduce the morphology of a phase-separated material, but as a rule of thumb, lower values of *q* represent larger domain sizes and vice versa. In-depth interpretation requires a plausible model of the shape and distribution of phases within the heterogeneous material, which requires additional information from other measurements or knowledge about the material.^122^ Most models belong to the following systems: dilute particulate systems, nonparticulate two-phase systems, periodic systems, and soluble blend systems.^122^ Using an appropriate model, it is possible to get information about size, shape, distribution, interface area, and eventually interphase thickness. Examples of the results of SAXS measurements, showing the change of domain size with changing chemical structure through macroCTAs in a PhIPS system^123^ or composition ratio in an IPN system,^124^ are presented in Figure 11.

### 4.2 Spatially Resolved Characterization

#### 4.2.1 Optical Microscopy

**Figure 12:**
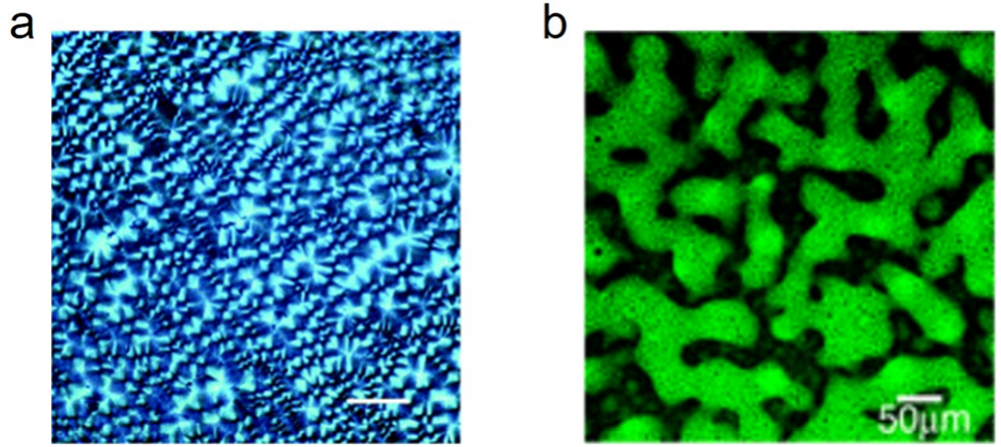
Optical microscopy of photopolymers. (a) Polarized optical microscopy of a lightly cross-linked semicrystalline thiol-ene. Scale bar 50 μm. Used with permission of Royal Society of Chemistry from ref (82). Copyright 2020; permission conveyed through Copyright Clearance Center, Inc. (b) Confocal laser scanning microscopy of a poly(cross-styrene)-interpoly(cross-methyl methacrylate) IPN. Fluorescent image (scale bar 50 μm). Reprinted with permission from ref (129). Copyright 2004 American Chemical Society.

In the realm of photopolymers, the significance of optical microscopy has high relevance for fractography, where the fractured surface of a tested sample is analyzed to determine the cause of failure. Especially if there are molecular orientations, semicrystalline or liquid-crystalline morphologies in the photopolymerized materials, polarized optical microscopy can be the instrument of choice (Figure 12a).^82^

However, the basic resolution of common optical microscopies challenges their utility in resolving heterogeneities at scales below the wavelength of their probe beams. Thus, advanced optical microscopy techniques, such as confocal laser scanning (CLSM)^36,65,125,126^ and near-field optical microscopy, (NSOM),^127^ which utilize the low wavelength of lasers to overcome these limitations, become of interest. Typically, a lateral resolution down to 120 nm and 20 nm can be achieved with CLSM and NSOM, respectively.

Moreover, optical microscopy can be used for imaging fluorescent dye distributions, particularly when these photopolymers are heterogenized by inks (Figure 12b).^128^ By visualizing the fluorescent dyes within the photopolymer matrix, optical microscopy plays a crucial role in elucidating the interfacial interactions, ink dispersion patterns, and distributions, which are essential in optimizing fabrication processes and analyzing the material’s performance.^100^

#### 4.2.2 Electron Microscopy

Electron-based microscopic techniques can provide images at length scales unachievable by optical microscopes. Scanning electron microscopy (SEM) is a well-known technique to check the spatial resolution of a printed part and detect surface features such as microcracks, elevated and steep locations, voids and cavities, and other characteristics that may serve as potential sites for crack formation and propagation.^130,131^

SEM visualizes the interaction of high-energy electrons with the surface. This function aids in distinguishing certain matrix-filled heterogeneities (fillers, fibers, etc.), enabling investigation of the distribution, specific geometries, and aspect ratios of fillers in photopolymers and the quality of their interaction. Such details are necessary to determine how the microstructure and mechanical properties relate.^132,133^

Moreover, SEM is ideal for detailed fracture surface analysis (microductility, roughness, cavities, etc.) to reveal types of material failure and the underlying toughening mechanisms. For example, SEM imaging showed that the core–shell particle debonding from the polymer matrix, which is related to the void growth mechanism of toughening, is the cause of a rougher fracture surface.^134^ Further toughening signs detectable by SEM are local microductility, hackle zones, and stream-like patterns.^135^ Notably, SEM imaging at low accelerating voltages is generally favored because a possible effect of the electron beam on the photopolymer is minimized. Moreover, since the electron–matter interaction volume beneath the surface is significantly larger than the beam spot, the generated pictures at low and high voltages can differ considerably.

**Figure 13:**
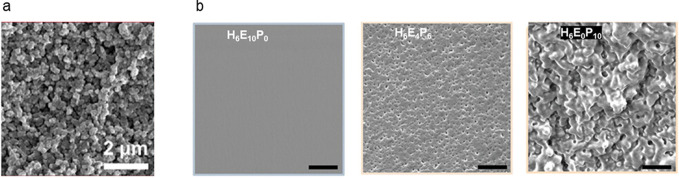
SEM to observe phase-separated morphologies. (a) Cryo-fractured cross-section of aphotopolymer based on pentaerythritol tetraacrylate (PETA), 2-ethylhexyl methacrylate (2-EHMA), and poly(propylene glycol) (PPG). The phase-separated PPG was removed by washing with methanol (scale bar 2 μm). Reprinted with permission from ref (136). Copyright 2023 Elsevier. (b) 3D-printable gels consisting of different ratios of hydroxyethyl methacrylate (H), poly(ethylene glycol) (E) and poly(propylene glycol) (P). Although in H_6_E_10_P_0_ no phase separation is evident, H_6_E_0_P_10_ and H_6_E_4_P_4_ clearly show phase-separated morphologies (scale bars 5 μm).^115^

Furthermore, to extend the morphological information to the third dimension, serial sectioning methods like focused ion beam (FIB) SEM or serial block face (SBF) SEM can be applied. However, visualizing the contrast of chemical heterogeneities, such as phase-separated domains with SEM is challenging due to the inherently low contrast of their interaction with the incident electrons. Selective contrasting or etching are beneficial techniques for overcoming this to some extent (Figure 13a). Alternatively, the evolution of phase separation can be followed for varying material composition, as its effect on fracture behavior appears in different morphologies compared to the homogeneous structures (Figure 13b).^115^

**Figure 14:**
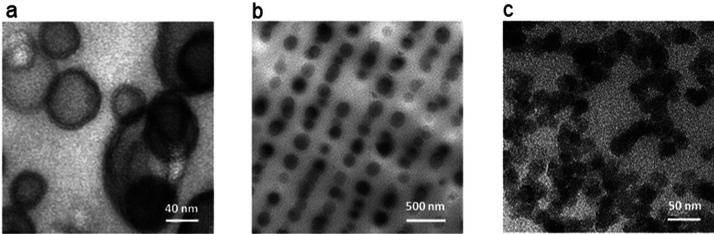
TEM observations of phase-separated morphologies. (a) Epoxy with 10 wt % PEB-*b*-PEO (sample RuO_4_ stained , scale bar 20 nm). Used with permission of Royal Society of Chemistry from ref (137). Copyright 2017; permission conveyed through Copyright Clearance Center, Inc. (b) Holographic polymer dispersed liquid crystal reflection gratings. Nematic droplets in a thiol-ene matrix (sample RuO_4_ stained, scale bar 500 nm). Reprinted with permission from ref (138). Copyright 2006 Elsevier. (c) SiO_2_ nanoparticles in a stereolithography resin (scale bar 50 nm). Reprinted with permission from ref (139). Copyright 2016 Elsevier.

The other classical electron microscopy method, transmission electron microscopy (TEM), offers higher resolution compared to SEM and the possibility to characterize ordered structures by electron diffraction and elemental analysis down to the atomic scale. Due to the requirement of transmittance, the sample thickness needs to be in the range of 50 to 200 nm. Such thin samples can be obtained by (cryo-)ultramicrotomy or FIB cutting. Typically, the natural mass–thickness contrast between the polymeric phases is very low because in most cases the polymer entirely consists of light elements. Therefore, the different phases should be stained selectively (Figure 14a and b). In the case of photopolymers filled with inorganic nanoparticles or fibers, no special contrast is needed to visualize the inorganic components (Figure 14c).^172^ As in SEM, serial sectioning methods can be applied to get three-dimensional information about the morphology in the nano range. Another opportunity to get this information is so-called electron tomography inside the transmission electron microscope.

#### 4.2.3 Atomic Force Microscopy

Atomic force microscopy (AFM) enables the characterization of surface features through the interaction of the sample surface with a very sharp cantilever tip and the measurement of that cantilever’s deformation. Along with the higher resolution of AFM compared to that of SEM, it measures the topography as well as mechanical properties, both of which are essential in analyzing the microstructure of heterogeneous photopolymers.

The topography of freshly fractured surfaces may determine the characteristics of the fractured surface and help to identify the failure mechanism. Moreover, the roughness of the fracture surface at the nanoscale can reveal the morphology of the photopolymer to some extent as the higher surface roughness is directly related to points of weakness preferred by propagating cracks to pass through.^140^

To measure the local mechanical properties, force–distance spectroscopy is another fundamental feature of AFM. In this mode, deflection of the cantilever during a gradual approach to the surface, applying a controlled force, followed by detachment to the starting position, is recorded. The shape of resulting force–distance curves delivers valuable information about the mechanical, adhesive, and elastic properties of the measured surface point.^141^ Recent powerful AFM tools can run this test over a larger sample area in the range of μm^2^ to generate images corresponding to the mentioned parameters.^142^ The tapping mode AFM, however, has been the most frequently used method in literature to evaluate the heterogenization of photopolymers.^108,109,143−145^ In this mode, the cantilever oscillates across the surface at its free frequency. Oscillating toward the surface finally results in the cantilever’s tip physically tapping the surface. While the oscillation amplitude is preset to reach a specific value (set point), it encounters delays (phase lags) as it engages with the surface. The energy damping during tapping typically varies in the nN range depending on the oscillation amplitude.^146,147^ This damping, identified as phase lag contrast, often correlates the lower and higher phase degrees to hard and soft areas, respectively.^111^ However, the opposite has also been documented, indicating that surface rigidity alone does not exclusively govern the damping.^125,148,149^ Other parameters, such as surface fluctuations (roughness), can affect the tip–surface interactions immensely. Successful AFM imaging is very sensitive to the sample’s surface quality, and a specific initial setting cannot consistently be applied to different samples.^150^ As a result, distinguishing rigidity and identifying the hard and soft domains is ideally achieved by tapping mode phase imaging combined with force distance spectroscopy.^151,152^ Alternatively, fully quantitative imaging modes are efficient.^153,154^

## 5 Microstructure and Fracture Mechanics

### 5.1 Failure Mechanisms in Traditional Photopolymers

**Figure 15:**
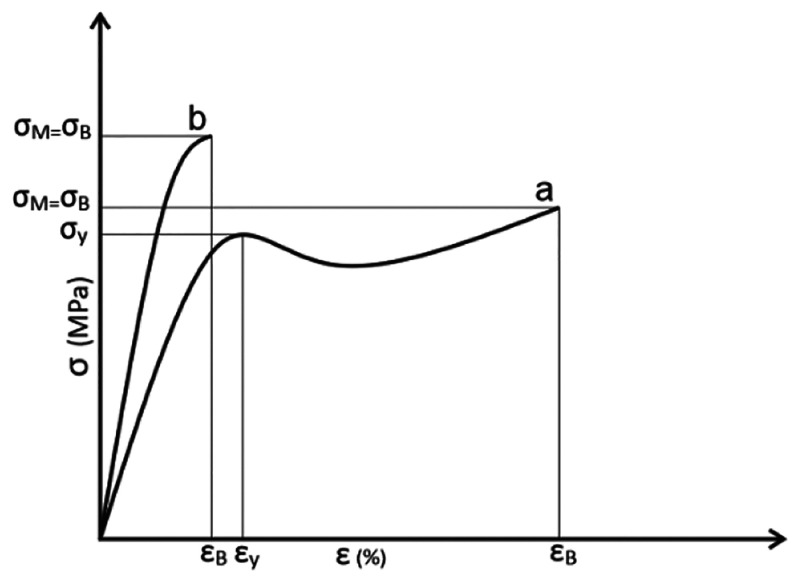
Schematic sketch of stress–strain curves of polymers with tough (a) and brittle (b) deformation behavior. In this example, the maximum stress (σ_M_) and strain (ε_M_) for both material types are equal to the stress (σ_B_) and strain (ε_B_) at break. However, the tough material may experience yielding at a certain strain (ε_*y*_), and its corresponding stress (σ_*y*_) is relatively high, sometimes even higher than the breakage stress (σ_B_). Adapted from ref (155) with permission from Hanser.

Understanding the material failure mechanisms in traditional photopolymers is essential for evaluating the impact of microstructural heterogenization on material performance. The fundamental mechanisms of failure in materials, in general, are characterized by brittle and ductile deformation behavior. Figure 15 presents typical stress–strain curves of ductile as well as brittle materials that starts with a linear regime where the slope represents the elastic (Young’s) modulus. For ductile materials, increasing stress triggers plastic deformation, which is characterized by considerable elongations and a decreased slope in the stress–strain curve. Pronounced yielding may also occur (yield strength/elastic limit), followed by a brief decrease in stress before strain hardening kicks in and increases the stress level again (Figure 15a). This type of deformation behavior can be observed in many thermoplastic materials, which behave elastically at room temperature but deform plastically or even rubber-like at elevated temperatures. On the contrary, brittle materials fracture without or upon only very little plastic deformation, which leads to a limitation of the stress–strain curve mostly to the elastic regime (Figure 15b). Many amorphous materials, including organic and inorganic glasses, typically show such behavior. This limits their use for structural engineering applications, where abrupt catastrophic failure without plastic deformation is unwanted. Photopolymers will in many cases also fail with very little plastic deformation due to their amorphous and highly cross-linked network structure, especially when the operating temperature is below the glass transition temperature of the material.

Low cross-linking densities, constituents with high molecular weight, and strong physical bonds are the key elements in achieving high energy dissipation (strength) and large elongation at break. Amorphous thermoplastic polyurethanes as well as semicrystalline thermoplastics such as polyamides and polyolefins are among the polymers that largely fulfill the mentioned requirements. However, in photopolymers used in AM, such as poly(meth)acrylates, meeting those requirements is challenged due to the formation of dense covalent cross-links. Additionally, the resulting networks are rather unregulated, leading to a wide range of bonding energies between the individual atoms of neighboring polymer chains. This leads to a wide glass transition range, preventing the formation of a pronounced yield point in the tensile diagram. Pronounced yield stress will occur if all physical bonds (e.g., hydrogen or van der Waals bonds) exhibit similar bond strengths. In this case, all bonds inside a uniformly loaded sample (e.g., a tensile sample) will break at a similar stress level, yielding a pronounced transition from the linear elastic regime to plastic deformation.

Additionally, many polymers in electronics, automotive, aerospace, and diverse industries must maintain their dimensional integrity even at temperatures exceeding 80 °C. Furthermore, 80 °C is a classical reference temperature for hot water applications. In this regard, the glass transition delivers estimates of the material’s suitability. Likewise, the heat deflection (or distortion) temperature (HDT) is relevant as it directly determines the temperature at which the material starts deforming under constant load. For amorphous structures, strong intermolecular forces between the chains significantly contribute to preventing softening and progressive distortion. Those forces may also assist in preventing chain slippage over time to avoid creep. Therefore, the combination of high elastic modulus, strength, and elongation at break, along with a high HDT, proves to be a significant challenge for the enhancement of photopolymers for AM.

Dynamic mechanical analysis (DMA) along with the tensile behavior of typical 3D-printable photopolymers at various temperatures can be used as a tool to predict their behavior during application. A typical 3D-printable photopolymer contains several key components:

- A mono- or multifunctional low molecular weight reactive diluent, which regulates the formulation viscosity,
- A bifunctional high molecular weight cross-linker, which counteracts brittleness, and
- A bifunctional high-*T*_g_ cross-linker that gives the printed part form stability.

**Figure 16:**
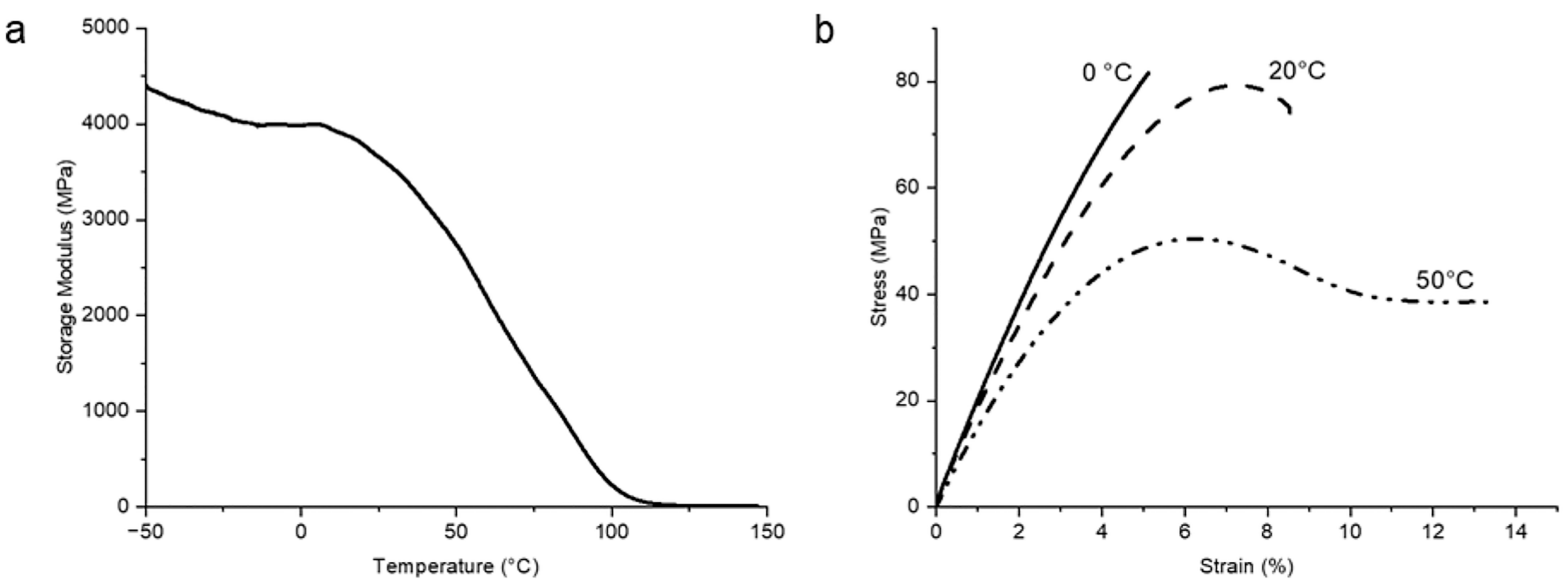
(a) Dynamic mechanical analysis (DMA) and (b) stress–strain curves of a 3D-printed photopolymer at different temperatures below and within the glass transition temperature range.

The reactive groups of all constituents are usually (meth)acrylates. The thermomechanical properties of such structures, however, are highly temperature dependent. As the DMA result of such a photopolymer shows, the glass transition range is rather broad, starting at around 10 °C and phasing out at 100 °C (Figure 16a). This differs significantly from engineering thermoplastics, where the glass transition usually starts above the targeted service temperature. The basis for developing the commercial 3D-printable photopolymers is to adapt the cross-linking density in such a way that the sweet spot of its mechanical properties lies at around 20 °C, leading to decent values in the corresponding datasheets. However, at lower temperatures (e.g., 0 °C) the material will become too brittle, and at elevated temperatures (e.g., above 50 °C for the photopolymer in Figure 16b) the material will soften rather quickly, losing creep resistance and elastic modulus.

The photopolymer shown in Figure 16a represents the state-of-the-art commercial AM photopolymers, demonstrating that the mechanical performance of currently available photopolymers relies on application temperatures where commonly a large proportion of the cross-linking network is still in the rubbery state. Hence, the overall performance of AM photopolymers remains severely limited in their operating temperature range, which needs to be improved compared to good engineering thermoplastics.

**Figure 17:**
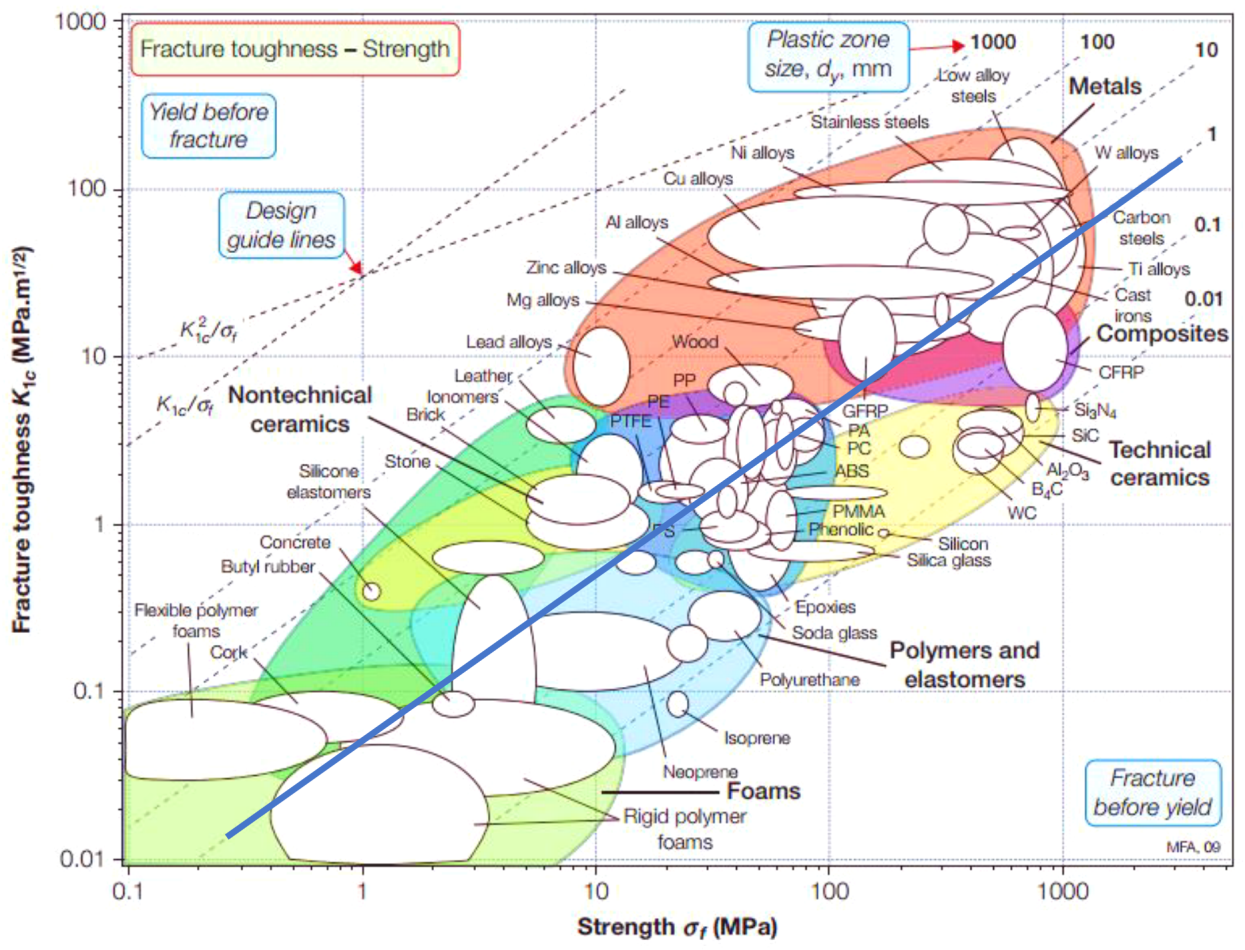
Ashby chart of fracture toughness *K*_Ic_ versus strength σ_f_. The 1 mm plastic zone line is highlighted to guide between brittle and ductile fractures of thermoset polymers. Adapted with permission from ref (156). Copyright 2017 Elsevier.

As mentioned, in most engineering applications, it is generally preferred for materials to undergo yielding before brittle failure. For ductile materials, plastic deformation can serve as a warning sign for impending failure by showing that the material is under significant stress and approaching its limits. In addition, ductile materials make it easier to design structurally more challenging parts. The Ashby diagram, as shown in Figure 17, illustrates the trade-off between a material’s fracture toughness (on the *y*-axis), representing its resistance to fracture, and its strength (on the *x*-axis).^156^ Materials closer to the top left of the diagram typically undergo yielding before fracture, whereas those closer to the bottom right are expected to fail before yielding. The placement of polymers in this diagram suggests that many homogeneous and high-strength polymers tend to fracture before yielding (closer to the bottom right). This positioning implies that further strengthening of polymers may increase their tendency toward brittle fracture. Moreover, the plastic zones (represented by diagonal lines in the diagram) for materials located in the bottom right such as thermosets (e.g., epoxies and phenolic resins) and certain thermoplastics (e.g., polystyrene, PMMA) are notably small (below 1 mm). This suggests their high tendency to fracture before yield, although they may show comparable (and even higher) elastic limits compared to high-performance polymers (e.g., polyamides and PTFE).

For 3D-printable photopolymers, fracture toughness values between 1 and 2 MPa m^0.5^ can be expected, in accordance with values measured for dental composites.^157^ Strength values of most commercially available photopolymers lie between 50 and 100 MPa, respectively. Taking into account the guidelines of Figure 17, it is obvious that photopolymers, which are currently used in AM fall into the brittle regime, severely limiting their usability as engineering polymers. Innovative toughening strategies, e.g., by employing heterogenization, will therefore be necessary to further develop the field.

In conclusion, Figure 17 suggests it is essential to enhance the plastic zone dimensions within high-strength polymers, including thermosets, for increased fracture toughness. Material scientific concepts for this purpose are discussed in the following section though for the sake of simplicity limited to linear elastic fracture mechanics. We then translate these material scientific concepts into suggestions for the molecular design of photopolymers. This translation enables chemists to utilize diverse chemical strategies to obtain rationally designed photopolymer structures, which are predicted to deliver optimal performance from the material scientific perspective. We then go on to identify these strategies in natural materials and engineering polymers, and determine which have the potential to be transferred to photopolymers. For each of the strategies, we will particularly highlight examples where the strategies have already been applied to photopolymers.

### 5.2 Linear Elastic Fracture Mechanics

Fracture toughness is defined as the material’s resistance against crack propagation at the crack tip. Assuming the material initially shows linear elastic behavior and brittle fracture without yielding, analytical solutions can be obtained by considering the balance of stored elastic energy versus the required energy to create a new fracture surface. The stress ratios around the crack tip can therefore best be described by the stress intensity factor *K*:
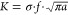
5where σ represents the macroscopic stress acting on the sample, *f* is a form factor describing the shape of the crack tip and *a* is the initial crack length. When stress and/or crack length are large enough to reach a critical stress intensity factor (*K*_c_), the part will fail. Thus, it is a measure of fracture toughness and varies from material to material. The mechanical load on the crack can be applied in three different loading modes: opening (mode I), sliding (mode II), and tearing (mode III). The opening mode, where stress acts perpendicular to the fracture surfaces, is the most common situation in engineering applications. This emphasis leads to the notation of *K*_c_ as *K*_Ic_ to denote the fracture toughness of materials, where the index I highlights the respective mode.^43^

**Figure 18:**
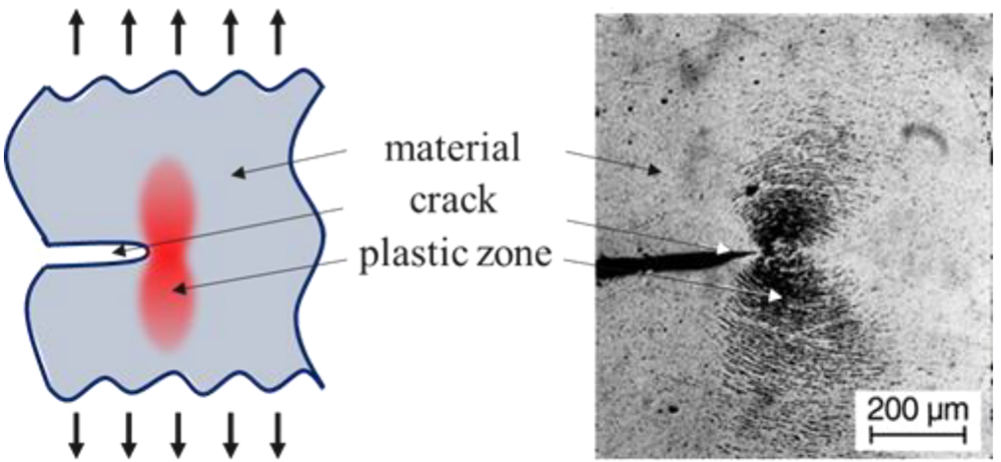
Schematic drawing of the plastic zone in front of the crack tip and an example of a plastic zone in PVC-C (chlorinated poly(vinyl chloride)). Reprinted from ref (154) with permission from Hanser.

In practice, the plastic zone at the crack tip can be considered a corresponding benchmark for the degree of fracture toughness. The size and shape of the plastic zone vary depending on the shape of the crack tip and the stress–strain characteristics of the material in use. Figure 18 illustrates the typical shape of the plastic zone for plane strain conditions and a plastic zone observed in the fracture mechanical test of polyvinyl chloride.

The following equation gives an estimate of the amount of dissipated energy *W*_*p*_ in the plastic zone surrounding the crack tip:
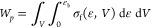
6

In this equation, *V* is the zone where plastic deformation takes place, σ_f_ is the strain and location-dependent flow stress and ε represents the elongation, with ε_b_ being the elongation at break. In this simple consideration, higher *W*_*p*_ can be achieved if one or more of the following conditions are optimized:

(1) The location-dependent yield stress (σ_f_) must be as high as possible.
(2) The deformation volume (*V*) must be as large as possible.
(3) The elongation at break (ε_b_) must be as high as possible.

It is noteworthy that in the case of additional ductility in materials characterized by a large deformation volume (plastic zone in front of the crack tip), a clear blunted crack tip, and significantly stable crack growth, alternative fracture mechanical concepts need to be applied. Among them are the crack tip opening displacement,^158^ the energy-based J-integral,^159,160^ and the essential work of fracture (for thin specimens with post-yield fracture).^161^ Within the field of photopolymers, however, the mentioned concepts are not so common for material development purposes. It should also be noted that the general approaches for determining “toughness” differ. In this Review, we refer to the following definitions of toughness-related terms:

(1) **Toughness** is a qualitative term, and its use in literature varies. Most commonly, toughness relates to the combination of strength and ductility.^162^ Materials exhibiting large strength and pronounced ductility are referred to as being tough.
(2) **Tensile toughness** is a quantitative measure that can be calculated from the area under the stress–strain curve obtained by a tensile test. This quantitative value can be easily calculated by numerical integration and is, therefore, commonly used. Since samples in tensile tests are not notched, tensile toughness is not a fracture mechanical value, although a large tensile toughness in most cases correlates with large fracture toughness. In the case of tensile toughness, it is highly recommended to consider not only the absolute values but also the shape of the stress–strain curve.
(3) **Fracture toughness** refers to the resistance against crack growth. As mentioned above, fracture toughness can be measured by standardized experiments, and depending on the ductility of the material, *K*_Ic_, *J*_c_, essential work of fracture, or crack tip opening displacement (CTOD) can be measured.
(4) **Impact energy** refers to the energy necessary to fracture a standard test piece under impact load.^162,163^ The test pieces can be notched or unnotched, respectively. Impact tests are highly useful for evaluating polymers: Due to the time–temperature correlation in the viscoelastic behavior of materials, polymers with a pronounced temperature dependence of storage and loss modulus might break ductile at a given temperature when strained slowly (as measured by tensile toughness) but become very brittle when the strain rate increases (as measured by impact toughness).

### 5.3 Toughening Concepts

Theoretically, a defect-free material exhibits superior fracture toughness because a large amount of energy is required to separate its atomic layers from one another and form the initial microcrack. The elimination of all stress concentrators (microcracks, voids, or surficial features), however, is extremely challenging and costly. In reality, adopting proper toughening strategies is material dependent. For thermoplastic polymers, those possibilities are well documented. Besides the modification of the molecular structure, i.e., molecular mass and its distribution, branching, (partial) cross-linking, and heterogenization of homopolymers and random copolymers are particularly powerful strategies. In multiphase materials such as blends, heterophasic block copolymers, and particulate composites, several energy dissipation mechanisms are present. Such mechanisms include crack stop mechanisms at the matrix–heterogeneity interface (Figure S1) as well as bridging, multiple crazing, multiple shear band initiation, multiple microcrack/void formation and debonding with void formation followed by matrix ligament yielding, or a combination of them (Figure S2).

**Figure 19:**
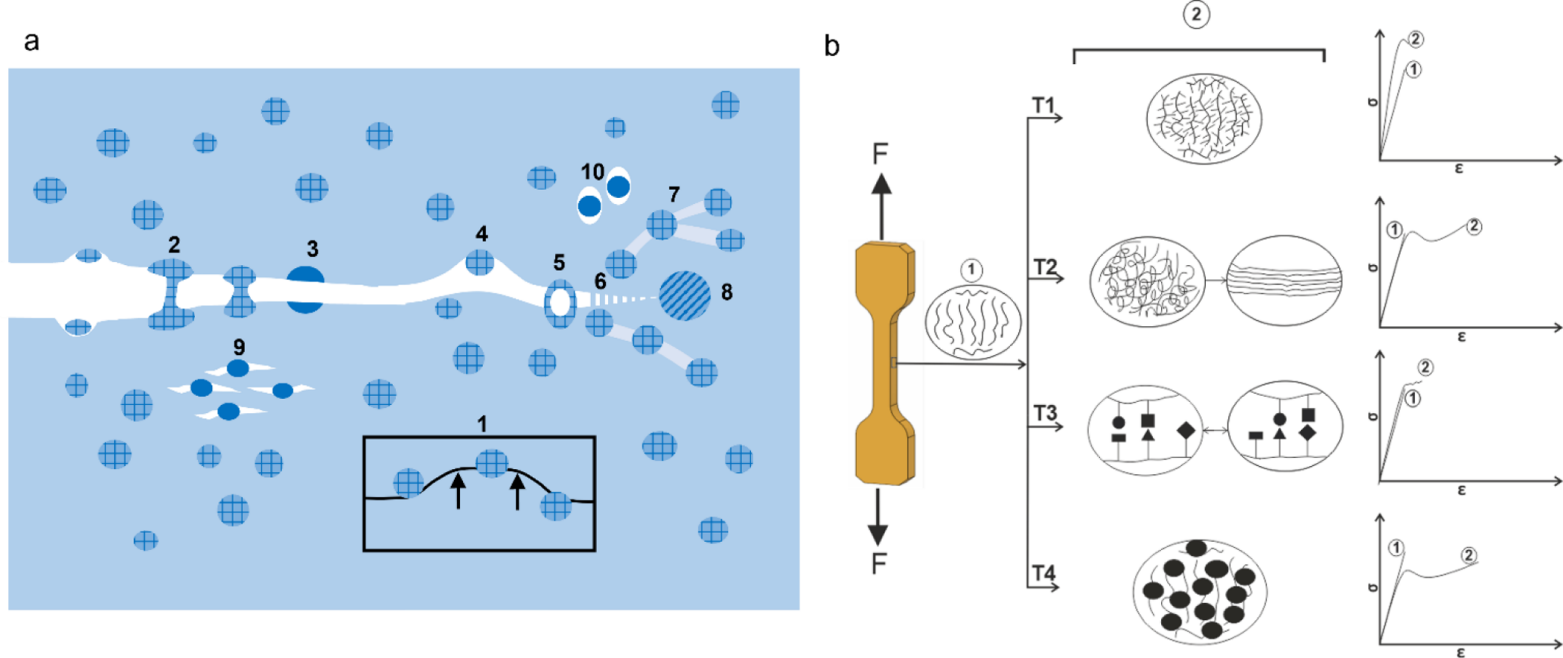
(a) Possible toughening mechanisms in modified epoxies according to refs (164−166). Epoxies are considered a typical example of highly cross-linked systems. The following energy dissipation mechanisms have been identified in such a system: 1, crack pinning; 2, bridging; 3, trans-particle fracture; 4, crack path deflection by particles; 5, cavitation of rubber particles; 6, crazing; 7, shear band formation; 8, plastic deformation of the crack tip; 9, microcracking, and 10, debonding of particles. (b) Four toughening concepts in high-strength materials: intermolecular interactions (T1), strain hardening (T2), reversible bonds (T3), and heterogenization (T4).

In principle, these mechanisms should also work in photopolymers provided they are not cross-linked, and a sufficiently high molecular weight is achieved. However, the literature lacks a systematic investigation of general toughening mechanisms in photopolymers. In highly cross-linked systems, which is the main fraction of photopolymers in industrial AM, all mentioned toughening concepts are not easily applicable due to limited chain movements and hindered polymer chain slipping. Nevertheless, there is evidence that some fundamental concepts can still apply to such systems as shown for epoxy systems (Figure 19a).^164−166^ Enhancing toughness in highly cross-linked systems is achievable by adding reactive rubbers, thermoplastics, rigid particles, or dendritic polymers.^167^ The mechanisms that contribute to toughening are crack pinning, particle bridging, crack path deflection, debonding, stretching and tearing of particles, microcracking, shear band formation, particle cavitation, trans-particle fracture, and crazing.^166^ Additionally, crack-stop mechanisms can occur at hard and soft particles or voids.

Looking at materials that effectively meet these criteria of maximum energy dissipation, we can identify four underlying chemical material design concepts (Figure 19b):

(1) **Intermolecular interactions (T1)**: Yield strength is high in all materials characterized by strong intermolecular interactions. Individual atoms or molecules can slip through these bonds and dissipate energy in the process. The higher the level of stress at which this slipping occurs, the more energy is dissipated. In crystalline systems such as metals, many sliding possibilities are advantageous in this respect. In amorphous systems, the molecular architecture will influence whether molecule sliding is prevented (low elongation at break) or possible (high elongation at break). The elongation at break of a polymer chain is reduced when side groups act as a steric barrier to mitigate the polymer chains from slipping (e.g., the phenyl group in polystyrene). However, this steric hindrance results in high resistance against hot forming and creep. Therefore, higher creep resistance typically accompanies increased elongation at break. High cross-linking densities have the same and an even more pronounced effect than sterically demanding side groups.

(2) **Strain hardening (T2)**: Another efficient strategy to enhance the deformation volume is using materials with pronounced potential for strain hardening. High molecular weight polymers may undergo strengthening mechanisms when the applied load accomplishes chain reorientation and alignment along the loading direction. Notably, the anisotropy induced in these polymers sets them apart from ceramics, inorganic glasses, and short-chain polymers, which do not exhibit significant hardening under load. In thermosets, comparable strengthening mechanisms are not present, providing an additional explanation for their relatively low fracture toughness.

(3) **Recoverable bonds (T3)**: The presence of reversibly opening bonds that allow atoms or molecules to slide is favorable for obtaining high elongation at break. The topological arrangement, on the other hand, dictates how the atomic components can slip, related to T1.

(4) **Heterogenization (T4)**: Heterogeneities are essential and arguably the most effective tool to shift the failure-controlling mechanism from fracture formation to crack propagation. The deformation volume and fracture toughness remain very low in particularly homogeneous materials (e.g., inorganic, or organic glass), where a crack always chooses the path of least resistance and spreads rapidly along the path of highest stress concentration. Amorphous materials (e.g., inorganic glasses, (meth)acrylate photopolymers, polystyrene) are therefore significantly more brittle than heterogeneous ones. In polymers, heterogeneities may be caused by intermolecular interactions, e.g., in the case of semicrystalline polymers. They can also be introduced through the addition of other materials such as nanoparticles, glass fibers, and core–shell elastomer-like particles to stop propagating cracks at the polymer matrix–heterogeneity interface before the crack critical size (*a*_c_) is reached.

The implementation of T1–T3 into 3D-printable photopolymers involves monomers with increased viscosity, setting a certain limit to these approaches in terms of processability during printing and postprocessing. T4 (heterogenization), in contrast, offers improvements in toughening without necessarily increasing the viscosity of the utilized resins. Concepts like photopolymerization induced phase separation are routes that enable the realization of heterogenization and will play an increasing role in the development of future resins for additive manufacturing.

### 5.4 Heterogeneous Microstructures at Different Length Scales

**Figure 20:**
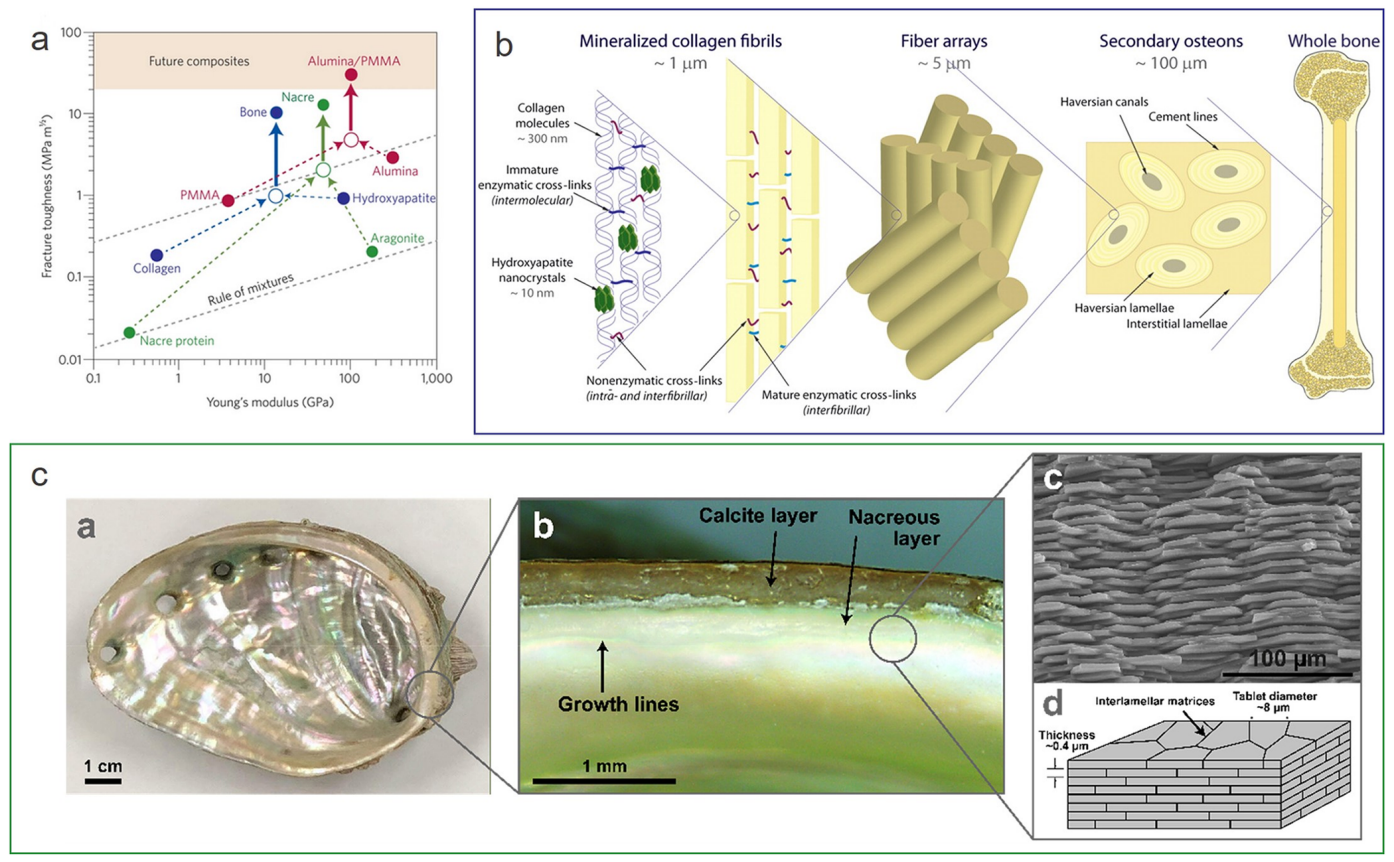
(a) Fracture toughness and Young’s modulus of bone and nacre compared to their pure components and mimicry of the concept in alumina-reinforced poly(methyl methacrylate) (PMMA). Reprinted from ref (175). Copyright with permission from 2015 Springer Nature. (b) Hierarchical organization of human cortical bone, representing the interaction of soft protein chains and hard minerals starting at the molecular scale. Reprinted with permission from ref (176). Copyright 2015 John Wiley and Sons. (c) Hierarchical architecture of nacre, where soft and hard domains form brick-and-mortar-like structures. Reprinted with permission from ref (189). Copyright 2019 Elsevier.

The success stories of common natural and synthetic materials suggest that their underlying principles of microstructure heterogenization should be studied to inspire the design of photopolymers in AM. It should be emphasized that herein are reviewed only works wherein heterogenization is used as a purposefully introduced instrument for enhanced material properties, and works that investigate heterogeneities as impurities or side effects are disregarded. High-strength biological materials in vertebrates and invertebrates exhibit hierarchical organization that evolved for a multitude of purposes including growth, protection, and movement.^168,169^ Natural (bio)materials are often superior compared to polymers in terms of fracture toughness to Young’s modulus compromise and compared to engineering composites and metals (Figure 20). It is noteworthy that this compromise adheres to the expected functions of the tissues. For example, the antler of the North American elk is among the toughest tissues reported with more than 25% elongation at break and serves elks to establish their power and dominance over one another through striking.^170,171^ While the impact function of antlers necessitates high fracture toughness for this tissue, their femur bones prioritize high modulus and static bending over fracture toughness to carry out their support function.

The proper balance of desired but conflicting mechanical properties in natural materials, such as high strength and fracture toughness, indicates the value of drawing inspiration from them in material design and arrangement for engineering applications.^172^ Notably, the pallet of available base substances to develop such diverse biomaterials is mainly confined to proteins, polysaccharides, and minerals.^173^ The organisms compensate for this shortage in resources through heterogenization, combining the available base substances in hierarchical architectures from the nanoscale upward. Below that level, the fracture strength becomes insensitive to heterogeneities and stress concentration disorders.^174^ In addition to size, other factors such as the quantity and orientation of heterogeneities govern the type and performance of biomaterials.^175^

The toughening concepts described in section 5.3 can be studied separately at each level of the hierarchy. For example, in cortical bone, which is foreseen to offer high strength and proper brittle fracture resistance, strong ionic interactions at the collagen–mineral interface and the intra- and interfibrillar cross-links restrict the chain sliding (T1) and enhance the elastic dissipative regime under deformation (Figure 20b).^176,177^ 30–70 vol% hydroxyapatite content of cortical bone demonstrates the impact of heterogenization (T4) on the mechanical properties and physiological performance of bone tissue.^178^ The model proposed by Buehler shows that mineralization via architectural embedding of hydroxyapatite starting at the molecular level is crucial for the bone to offer its impressive mechanical multifunctionality,^179^ while other resemblances of T1–T3 toughening mechanisms emerge on higher hierarchical levels.^180^ Cortical bone also employs sacrificial bonding at different hierarchical organization levels starting at the tropocollagen level (T3).^181,182^ The role of the cross-linking density of such sacrificial bonds is of great importance in the overall fracture behavior of bone as it ages, with more cross-linked regions in older bone leading to fracture behavior changes from tough to rather brittle.^183^ Likewise, the nacre’s remarkable fracture toughness is attributed to its brick-and-mortar-like microstructure (Figure 20c). In analogy, nacreous multilayer structures can be compared to a book as a pile of pages. Due to this structure, a specific crack cannot easily spread, and crack propagation requires crack nucleation in every layer. This showcases how the drastic fluctuations of modulus in multilayer biomaterial combined with the proper interaction of layers enhance the overall fracture toughness (*K*_c_).^184^ Similar mechanisms were suggested for other natural materials such as teeth and turtle carapaces, emphasizing the deterministic role of the mentioned toughening concepts on their ultimate functionality.^178,185−188^

**Figure 21:**
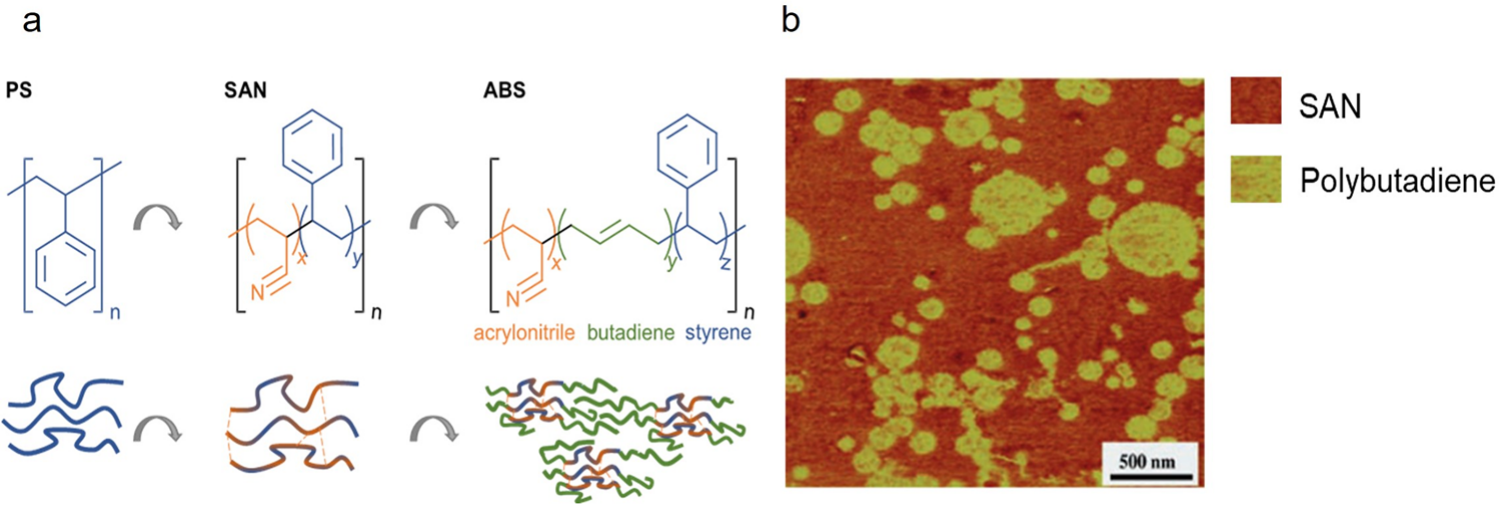
(a) Schematic representation of the microstructural changes from polystyrene (PS, left) to acrylonitrile-butadiene-styrene (ABS, right) through copolymerization and (b) microscopic image of the SAN matrix with soft PB embedded after heterogenization by phase separation. Adapted with permission from ref (190). Copyright 2014 John Wiley and Sons.

Figure 20a also vividly demonstrates for the case of alumina/PMMA that the learnings from natural materials can be transferred to synthetic materials. Another engineering example is acrylonitrile-butadiene-styrene (ABS). Atactic polystyrene (aPS) is an amorphous thermoplastic material with phenyl side groups attached to a simple aliphatic backbone (Figure 21a, left). The steric barrier between the polymer chains caused by the phenyl side groups prevents the polymer chains from sliding at low mechanical loads, resulting in a high stiffness and heat resistance. For demanding applications with high mechanical loads, the fracture toughness and elongation at break are, however, inadequate (2–3%, Table 1). Increasing the intermolecular interaction between the polymer chains (T1) should increase mechanical strength, which can be achieved by copolymerizing styrene with acrylonitrile. The enhanced intermolecular interaction in styrene-acrylonitrile (SAN, Figure 21a, middle) is achieved through interactions between the polar nitrile side groups. The differing electron affinities of carbon and nitrogen form strong dipoles that significantly increase the attraction between the polymer chains.

**Table 1:** Thermomechanical Properties of Polystyrene (PS), Styrene-Acrylonitrile (SAN), and Acrylonitrile-Butadiene-Styrene (ABS)^191^

Property	Unit	PS	SAN	ABS
Young’s modulus	MPa	3000–3500	3500–3900	2200–3000
Strength	MPa	40–66	61–79	25–65
Elongation at break	%	2–3	2.5–4	8–20
HDT/A	°C	65–85	100	100

The considerable increase in strength due to this attraction is shown in Table 1. Based on this data, the elongation at break in SAN has improved but not significantly, as SAN’s structure is still amorphous. SAN and PS also have high molecular weights, providing the conditions for strain hardening characteristics (T2). However, this mechanism cannot become sufficiently active in amorphous, homogeneous materials. In fact, a growing crack can propagate unhinderedly as the plastic zone is still rather small despite material hardening. Thus, the fracture toughness will only increase slightly, and a large potential for dissipating energy will remain unutilized.

To effectively utilize the strengthening potential, heterogenization of the material (T4) is essential. The addition of butadiene to the monomer mix achieves exactly this in acrylonitrile-butadiene-styrene (ABS, Figure 21a, right). The butadiene group’s inherent apolarity makes it incompatible with the polar nitrile and aromatic phenyl group. Phase separation therefore occurs upon melt solidification, enabling the formation of soft phases of poly(butadiene) phases within an amorphous hard SAN matrix (Figure 21b).

As a result, a high-strength material is obtained with high elongation at break and characterized by strong intermolecular interactions between aromatic groups (T1), strain hardening characteristics due to high molecular weight chains (T2), and most importantly pronounced heterogeneity provided by the induction of soft butadiene groups (T4). ABS offers a massively increased elongation at break at comparable strength values and hence increased fracture toughness compared to homopolymers of the ABS monomers (Table 1).

**Figure 22:**
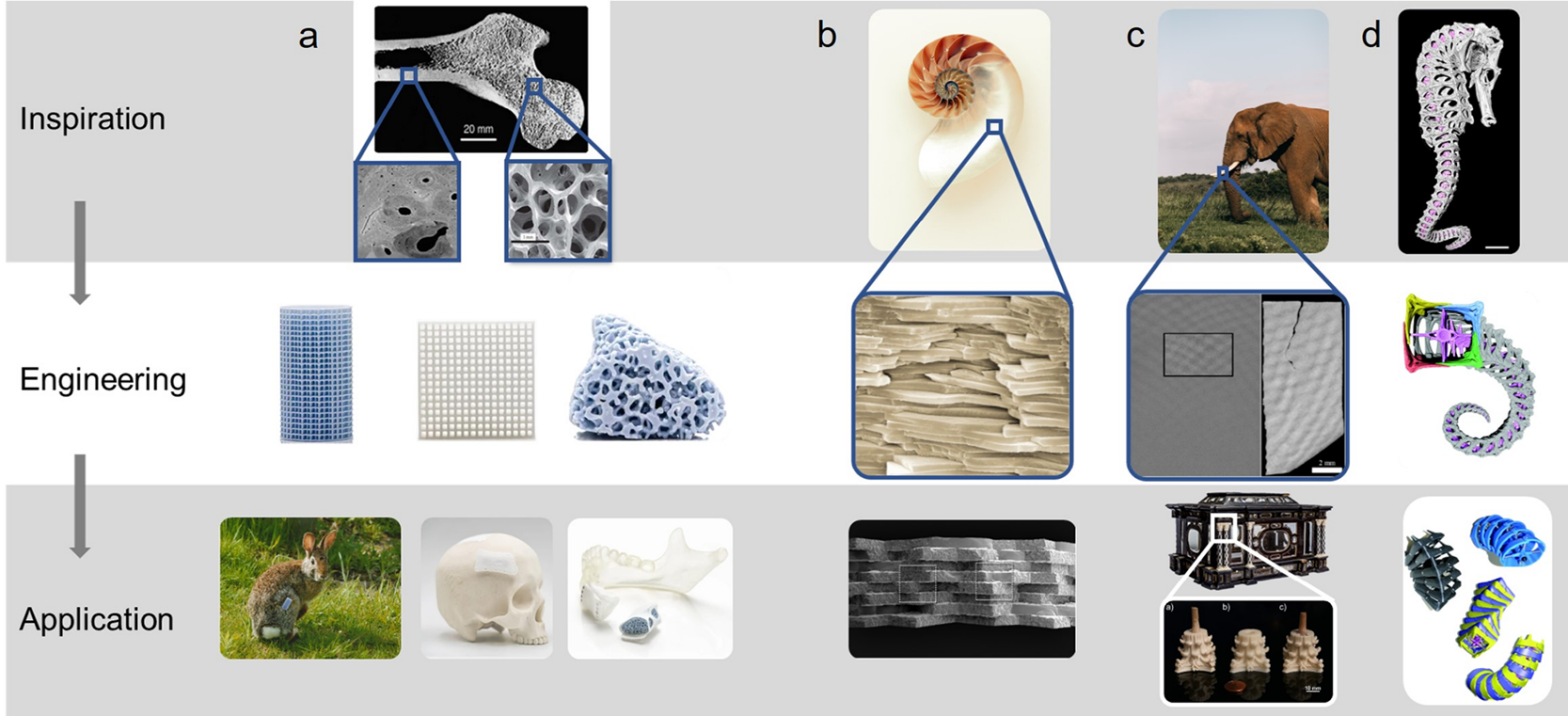
Examples of biomimicking through AMTs for different applications. (a) Femur bone (top) including concentric osteons (left). Adapted with permission of Royal Society of Chemistry from ref (204). Copyright 2004; permission conveyed through Copyright Clearance Center, Inc.; and spongy bone (right). Adapted with permission from ref (168). Copyright 2007 Elsevier. The printed structures mimic natural bone (middle), and AM fabricated bones find application as grafts (bottom), courtesy of Lithoz GmbH. (b) Nacre (top) with brick and mortar-like structures (middle). Adapted with permission from ref (175) Copyright 2015 Springer Nature. Mortar-bone structure inspired AM structure (bottom). Reprinted with permission from ref (205). Copyright 2017 John Wiley and Sons. (c) Hierarchical structure of ivory. Adapted with permission from ref (206). Copyright 1999 Elsevier. Inspired capitals (bottom), 3D-printed for the shrine for Friedrich III of Austria.^197^ (d) Cross-sectional architecture of seahorse (top), 3D-printed (bottom) to investigate its geometrical advantage over the cylindrical geometry (middle). Adapted with permission from ref (207). Copyright 2015 AAAS.

The development of multimaterial AMTs based on photopolymers in recent years is a result of the high effectivity of proper heterogenization in delivering multifunctional materials.^192,193^ The underlaying microstructures of high-strength biomaterials such as femur bone (with spongy and concentric features^194^), nacre (brick and mortar-like structure^195−197^), ivory,^198^ antlers (fibrillar),^199^ etc. have been inspiring sources for developing man-made engineering structures by AMT (Figure 22).^25,200−202^ Photopolymer-based AMTs enable processing on the same length scales as living organisms in nature.^203^ Moreover, biocompatibility, processability under ambient conditions, and high chemical diversity of photocurable systems have made them an excellent choice for mimicking high-strength natural materials.

From the process–structure relationship point of view, the associated rapid cross-linking with AM-based photopolymerization gives rise to inhomogeneous network architectures with various stress-concentrating centers. Such challenging defects can lead to the brittle deformation behavior of photopolymers. The microstructural imperfections also explain why thermoset polymers (e.g., phenolics, epoxies, and cross-linked acrylates) are orders of magnitude lower in fracture toughness compared to polymers with homogeneous architecture (e.g., polypropylene) or engineering plastics (e.g., ABS, polycarbonate, or polyamide). Therefore, the clever design of chemistry and processes is required to harvest the potential of photopolymer-based additive manufacturing.

## 6 Heterogenization Strategies

Although polymer heterogenization is a highly active field, the examples of photopolymer heterogenization are still relatively rare. Due to the tremendous success of this field for polymers in general and the new challenges awaiting photopolymers on the stepping stone from 2D coating applications to 3D functional parts specifically, however, we expect a fast and steady increase of research activities on heterogeneous photopolymers and AMTs enabling such microstructural control of photopolymers.

Heterogenization strategies can rely on either physical or chemical heterogenization. Traditional heterogenization methods such as the addition of fillers to polymers to obtain so-called composites are well-known examples of physical heterogenization. They rely on physical interactions such as van der Waals forces and/or on hierarchical microstructuring, e.g., via AMTs. While these strategies have been identified as toughening mechanisms for a long time, heterogenization relying purely on polymeric materials has been much less exploited so far. Chemical heterogenization is a result of the chemical structure of a macromolecule (e.g., semicrystalline polymers, block copolymers), which induces (self)assembly in the form of crystallization or phase separation and/or further structural changes during the polymerization process (e.g., interpenetrating networks, polymerization-induced phase separation). Physical heterogenization of photopolymers may cover applications unattainable for chemical heterogenization and vice versa. For example, polymerization-induced phase separation can be employed to a microstructural level that does not make the material opaque, as is typically the case for physical heterogenization, e.g., with fillers. This could be useful for applications that require transparent products. However, the products achievable by chemical heterogenization may still not fulfill all desired properties of engineering polymers. Furthermore, chemical approaches, which shift the polymerization mechanism from radical-based polymerization to step-growth-like mechanisms, may not always be attractive for industrial-scale manufacturing yet. Therefore, research in both areas promises materials with previously unattainable features.

In this section, the reviewed literature focuses on advances in heterogenization techniques for photopolymers. Examples of additively manufactured heterogeneous photopolymers will be emphasized where available. Additionally, successful heterogenization concepts for polymers in general will be introduced, which have already served or will serve as incentives to translate the strategies to (vat) photopolymerization in the future.

### 6.1 Physical Heterogenization

#### 6.1.1 Fillers

The use of additives is a state-of-the-art technique to improve the overall performance of polymeric materials. The range of fillers for photocurable systems intended for vat photopolymerization is as diverse as that of thermoplastic systems (e.g., ceramic powders,^208,209^ carbon particles,^210,211^ fibers,^212^ metallic materials,^213^ organic microspheres,^214^ hydroxy-apatite,^215^ cellulose nanocrystals,^208^ carbon nanotubes^177^ and graphene oxide,^178^ fibers,^212^ clay,^139,216^ and silsesquioxanes^217^). These fillers affect thermomechanical behavior, creep, optical properties, electrical conductivity, and magnetism.^218−221^ Compared to thermoplastic systems, the key point in selecting the type, size, geometry, color, and amounts of fillers is that they may jeopardize the curing depth and precision due to light scattering and absorption of the fillers. It is essential for printing speed and precision to minimize such effects. Furthermore, homogeneous dispersion of the filler throughout the printing process must be ensured for uniform parts and reproducible results.

Recent reviews on physically heterogenized photopolymers indicate that most resins include fillers on the nanoscale.^222,223^ This is mainly due to the exceptional dispersibility of nanofillers, which improves interfacial contact with the host matrix and allows for the emergence of photopolymers with unique characteristics (e.g., conductivity, electromagnetism, and reinforcement). Furthermore, compared to conventional fillers, nanofillers reduce the adverse effects of filler incorporation on the shape accuracy of the final printed part. To increase the matrix–filler interaction, chemically embedded fillers have also been utilized.^224^

Notably, most of the reinforcing nanofillers increase stiffness and tensile strength.^225−228^ Furthermore, plastic deformation and impact resistance can be facilitated by introducing liquid rubbers, e.g., carboxyl- (CTBN), amino- (ATB/ATBN), epoxy- (ETB/ETBN) or vinyl- (VTB/VTBN) terminated butadiene-acrylonitrile, to the polymer.^44^ Alternatively, polymer particles based on polyesters with a molecular weight of 100–2000 kg mol^–1^ have been described for the 3D-printing of photocurable colloids.^229^

The toughness improvement in such blends highly depends on their critical volume fraction and effective geometry.^230^ Liquid rubbers are highly prone to coalescence and agglomeration, which can lead to uncontrollable phase separation during the course of polymerization. A decent way to mitigate these effects is to cover them beforehand with rigid polymers in emulsion polymerization. By adjusting polymerization parameters, the size of the resulting so-called core–shell particles (CSPs) as well as their surface chemistry are adjustable. The consequentially achieved adhesion to the resin on the one side, combined with the contrasting properties of the filler on the other side, contributes significantly to the fine-tuning of the overall mechanical properties of the photopolymer material. For example, the synthesis of CSPs based on polysiloxane rubber particles with coreactive groups on the surface was claimed in 2003.^231^ Following the patent, extensive research and development efforts were devoted to this subject.^134^ In a recent case, including 7% CSPs introducing the epoxide groups to the outer shell in an epoxy-based SL resin resulted in a more than 194% increase in fracture toughness.^232^ Notably, the impact of core–shell particles on the fracture toughness of photopolymers has mostly been studied in epoxy-based resins, a subject that has been reviewed recently.^167^

Furthermore, fillers represent one of the main concepts to mitigate shrinkage stress in photopolymers via heterogenization (T3). Most commonly, the functional group density per volume unit was reduced by adding fillers such as prepolymer powders,^233^ silica,^234^ clay nanoparticles,^235^ and short glass fibers^236^ into the photopolymerizable matrix. Soft nanogels are another example of fillers that reduce the shrinkage-prone material fraction and can also relax the chain conformation at the matrix–nanogel interface.^49^ Fillers can reduce the shrinkage in photopolymers by increasing the overall volume of the curing formulation. Moreover, they may impede chain slippage and restrict the network’s ability to undergo shrinkage (T1). Furthermore, the reduction in the polymerization rate of filler-containing formulations can significantly reduce the shrinkage in photopolymers.^107,237,238^

When vat photopolymerization is paired with fillers, three major challenges occur: scattering and absorption of light by the filler, increase in viscosity, and agglomeration of the filler.^4,223^ While AMTs generally enhance the processability of photosensitive suspensions by mitigating the low curing depths due to scattering and absorption through the layer-based curing mode, attainable layer thicknesses and resolution may still be impaired for such formulations compared to homogeneous formulations. These aspects limit the amount of filler that can be added to a formulation. Thus, the optical properties of fillers need to be addressed further. Different particles show unique light interactions, mostly influenced by their size and absorptivity. For example, a higher energy dose is necessary for efficient curing when the resin contains light-absorbing fillers such as carbon-based or conductive metals.^239^ In contrast, the incorporation of small UV-transparent additives significantly increases photopolymerization efficiency and 3D-printing resolution.^240^

As conventional vat photopolymerization devices operate best with lower formulation viscosities, the higher viscosity of filler-containing formulations may adversely affect the printing process. Here, there is a need for new AMTs to process high-viscosity resins. The recent developments of light-based AMTs enabling temporary viscosity-lowering parameters (i.e., heat in hot lithography and shear rate in viscous lithography manufacturing) present significant progress.^53^

Moreover, maintaining suspension homogeneity over an extended printing process, which involves challenges like agglomeration, void formation, etc., demands optimization of filler/resin interfaces. In a systematic study, the surfaces of SiO_2_, montmorillonite, and attapulgite (as 0–2 nano dimension fillers) were studied to discover the effect of filler geometry on processing and properties of the (meth)acrylate-based resins for SL.^139^ The results demonstrated that attapulgite nanorods and exfoliated organic montmorillonite inhibit the polymerization progress and delay the gel point through strong absorption and scattering of the incident light. For nanosphere silica, however, the reinforcing effect is predominant, gelation remains unaltered, and a higher elastic modulus was achieved. The distribution of fillers in the final photopolymers may also be affected by the kinetics of network formation and the possibility of phase separation. For example, phase separation was observed during the photopolymerization of trimethylolpropane triacrylate in the presence of suspended nano-SiO_2_, resulting in silicon nanoparticle-rich and -poor phases.^241^ Similar morphologies were also observed for different nonreactive thermoplastic fillers in methacrylate-based photosensitive formulations.^107^

It was shown that these filler-distribution effects govern the ultimate material characteristics of the resulting photopolymers.^107^ Thus, the extent to which fillers can improve the mechanical performance of photopolymers highly depends on their proper dispersion and arrangement within the matrix. Even highly potent metal fillers may worsen the overall mechanical properties if the filler–matrix bonding is weak.^242^ Similar to conventional composite systems, filler functionalization is intended to strengthen photopolymers by increasing the affinity of fillers to theresin compared to themselves, e.g., via silanized thiourethanes on the surface of inorganic fillers (T1),^243^ via the use of reversible covalent bond exchange reactions at the silica–thiol-ene resin interface to relax applied stress and increase toughness (T3),^244^ or via the functionalization of fillers with reactive groups to covalently embed them in the polymer matrix.^224^ Overall, better strength and modulus can be obtained from photopolymers enriched with compatible fillers. The addition of fillers may also result in improved fracture toughness of photopolymers if implemented correctly.^232,245−247^

#### 6.1.2 Inkjet Printing

In material jetting, the printer’s nozzle(s) release the formula at specific points onto the substrate to solidify upon exposure to light.^248^ As mentioned previously, the number of materials within the printing part can be increased by utilizing more inks and nozzles. This gives rise to exciting opportunities for tailoring processing–properties relationships and particularly biostructures’ architectures.^249^ One example includes printing interpenetrating phase composites (IPCs), including glassy and rubbery photopolymers, using multimaterial inkjet technology. Strategic manipulation of volume fractions of hard and soft phases, as well as the consideration of printing orientations of lattice symmetries, has suggested a new avenue for tailoring fracture toughness.^250^ Another example includes using multimaterial inkjet printing to fabricate the resemblances of the soft and hard phases of common tough biostructures.^251^ Examples of heterogeneous biostructures mimicked by polyjet printing include periodic brick and mortar (nacre), concentric hexagon (bone osteon, annual plane rings), cross and branch-lamellar (conch shell), and rotating plywood (stomatopod dactyl club) structures.^252,253^ The materials exhibited continuous functional gradients rather than stepwise property changes. These bioinspired photopolymers were manufactured using a voxel-based multimaterial inkjet printing technique.^193,251^

However, there are limitations associated with multimaterial inkjet printing. The rheological preconditions for inks and the physical characteristics of inks limit their extensive use.^254,255^ Related to this issue, the risk of clogging during the printing process also limits the incorporation of fillers within the inks to specific nanofillers.^248,254^ As a result, the contrast between the mechanical properties of printed parts cannot quite achieve the contrast in biostructures to date.

**Figure 23:**
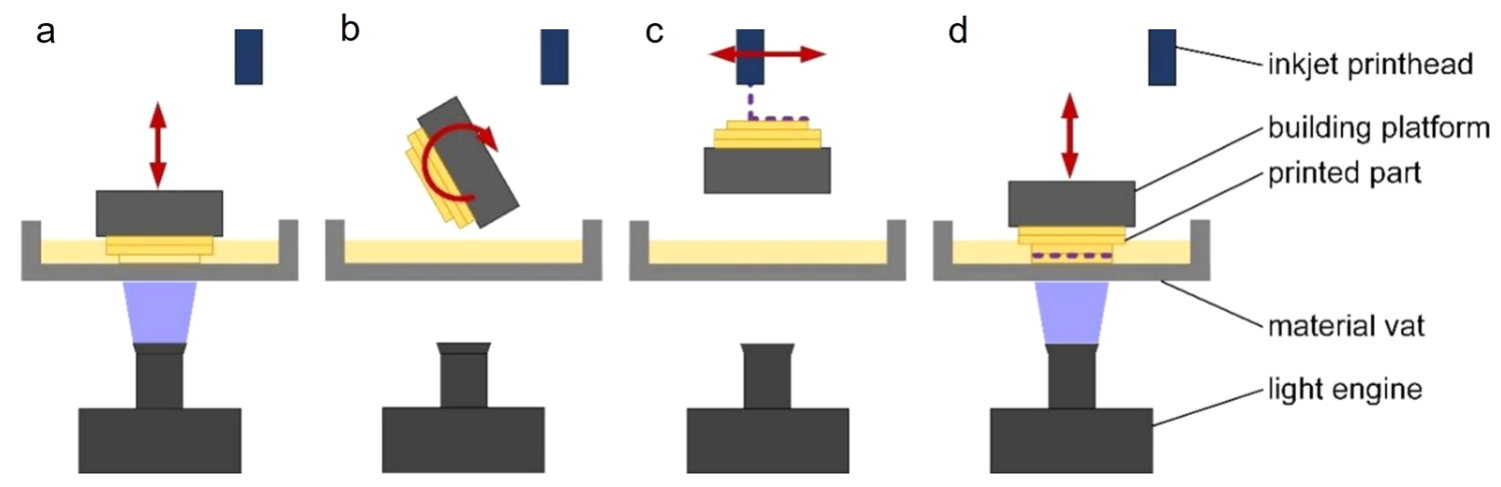
Workflow of the hybrid printing system consisting of vat photopolymerization and inkjet printing. (a) The resin layer is printed, (b) the building platform rotates upward, (c) the ink layer is jetted, and (d) the building platform rotates downward for the next layer to be printed. Adapted from ref (27).

Hybrid vat photopolymerization-ink-based technologies are promising avenues to alleviate these limitations in the future. In this approach, the depositing substrates for inkjet are the printed layers by vat-based techniques. For example, the integration of DLP with inkjet printing, as shown in Figure 23, was utilized to incorporate very thin layers of soft inks within a hard (meth)acrylate-based matrix and mimic the tough lamellar structure of spicules.^27^ Similarly, the use of DLP with direct ink writing (DIW) in a hybrid form to manufacture functional composites was reported,^256^ and an inkjet hybrid system has been claimed, where a soft polysiloxane-based monomer is jetted into a hard matrix to increase the overall toughness of the system.^257^

Notably, to achieve high resolution of heterogeneous structures for all of the approaches described above, challenges related to the physical properties of inks^258^ and the inhibition effects of oxygen need to be addressed going forward. When the droplet size is smaller, more surface area is exposed to stray oxygen, leading to less control over the integrity of the deposited droplet and its optimum mechanical properties.

### 6.2 Chemical Heterogenization

The foundations of chemical heterogenization of photopolymers are well-represented in high-performance thermoplastic polymers: Semicrystallinity explains the excellent mechanical performance of industrial polymers such as polyethylene or isotactic polypropylene.^259^ Poly(styrene-*b*-butadiene-*b*-styrene) (SBS) is the most prominent example of block copolymers that shows high impact resistance and in some cases high transparency.^260,261^ Interpenetrating networks (IPNs) could be seen as the photopolymer pendant of thermoplastic polymer blends. Thermoplastic polyurethanes are prime examples for describing microphase separation and its impact on thermomechanical performance.^262^ In the following, the concepts related to each of these chemical heterogenization approaches will be introduced and their presence in photopolymers and vat photopolymerization will be explored.^263^

#### 6.2.1 Semicrystalline Polymers

The long aliphatic chains of polymers make it difficult to achieve ideal crystallinity. Thus, engineering polymers are typically semicrystalline, i.e., crystalline domains form in an otherwise soft amorphous matrix.^264^ This is beneficial to the mechanical performance because material stretching can occur to a certain extent within the soft phase, where chains slide past each other until the maximal elongation of an amorphous chain between two crystalline domains has been achieved. Lessons learned from traditional engineering polymers to obtain excellent thermomechanical performance are clear: The most important material parameters influencing crystallization behavior are the chemical composition of the main chain and the polymer architecture.^265^ From an enthalpic point of view, the implementation of heteroatoms introduces intermolecular interactions, which aid crystallization. Main chain flexibility increases the entropic contribution. Steric hindrance reduces a polymer’s crystallization ability drastically. Furthermore, material processing and treatment are crucial for obtaining and preserving the semicrystalline phases within the material. Translating these lessons to photopolymers, however, has only begun gradually. Radical polymerization does not readily offer main chain functionality. Their side chains additionally introduce steric hindrance. Of course, this tendency of steric hindrance increases further if networks are formed during photopolymerization. Thus, the implementation of semicrystallinity into photopolymer networks is extremely challenging.

**Figure 24:**
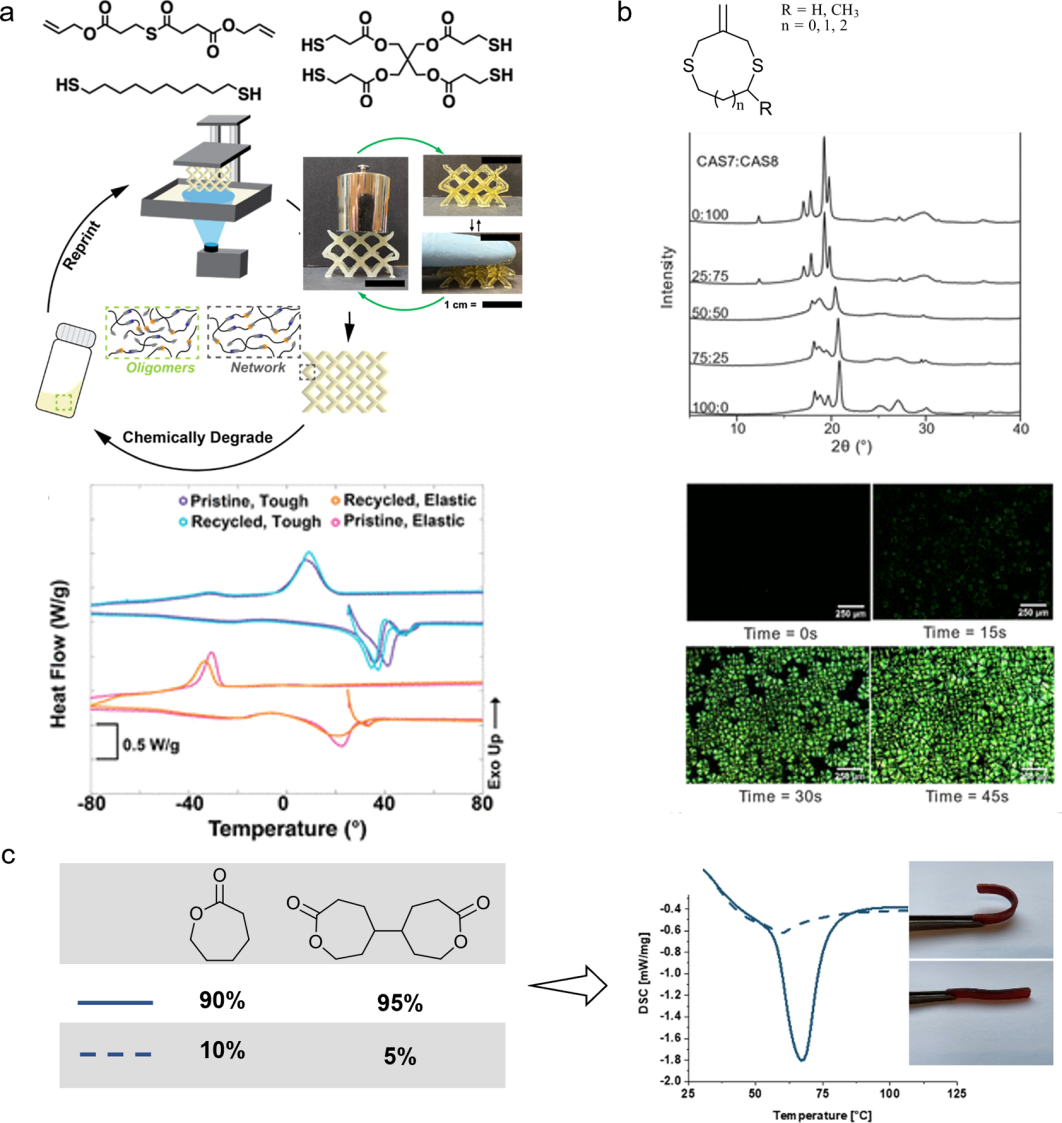
(a) Semicrystalline thiol-ene networks are printed and recycled. Their semicrystallinity was evident in DSC experiments. Reproduced with permission from ref (83). Copyright 2023 American Chemical Society. (b) Semicrystalline radical ring-opened photopolymer networks characterized by SAXS. Reprinted (adapted) with permission from ref (267). Copyright 2023 American Chemical Society. (c) Semicrystalline cationically ring-opened photopolymers with varying cross-linker content for shape-memory and recycling applications. Crystallinity was determined by DSC. Reprinted with permission from ref (29). Copyright 2022 Wiley.

Thiol-ene concepts have become an important example of semicrystalline photopolymers, which have also been printed in a stereolithographic process (Figure 24a).^82,83^ Very recently a thiol-ene monomer system has been claimed to form a linear semicrystalline thermoplastic material with outstanding mechanical properties.^266^ Furthermore, radical ring-opening of cyclic allyl sulfides photopolymerization to obtain semicrystalline photopolymers has been successfully used in SL (Figure 24b).^267^ These efforts have recently been translated to cationic ring-opening photopolymerization at elevated temperatures by our group, which exhibited shape-memory behavior (Figure 24c).^29^ Furthermore, the self-assembly of block copolymers in a photopolymer matrix was also utilized to generate crystallinity in supramolecular networks with tunable deformation capacity.^268^ Based on these chemistries, high-quality recyclable thermoplastic parts were printed via SL.^82,83,268^

#### 6.2.2 Block Copolymer Structures

**Figure 25:**
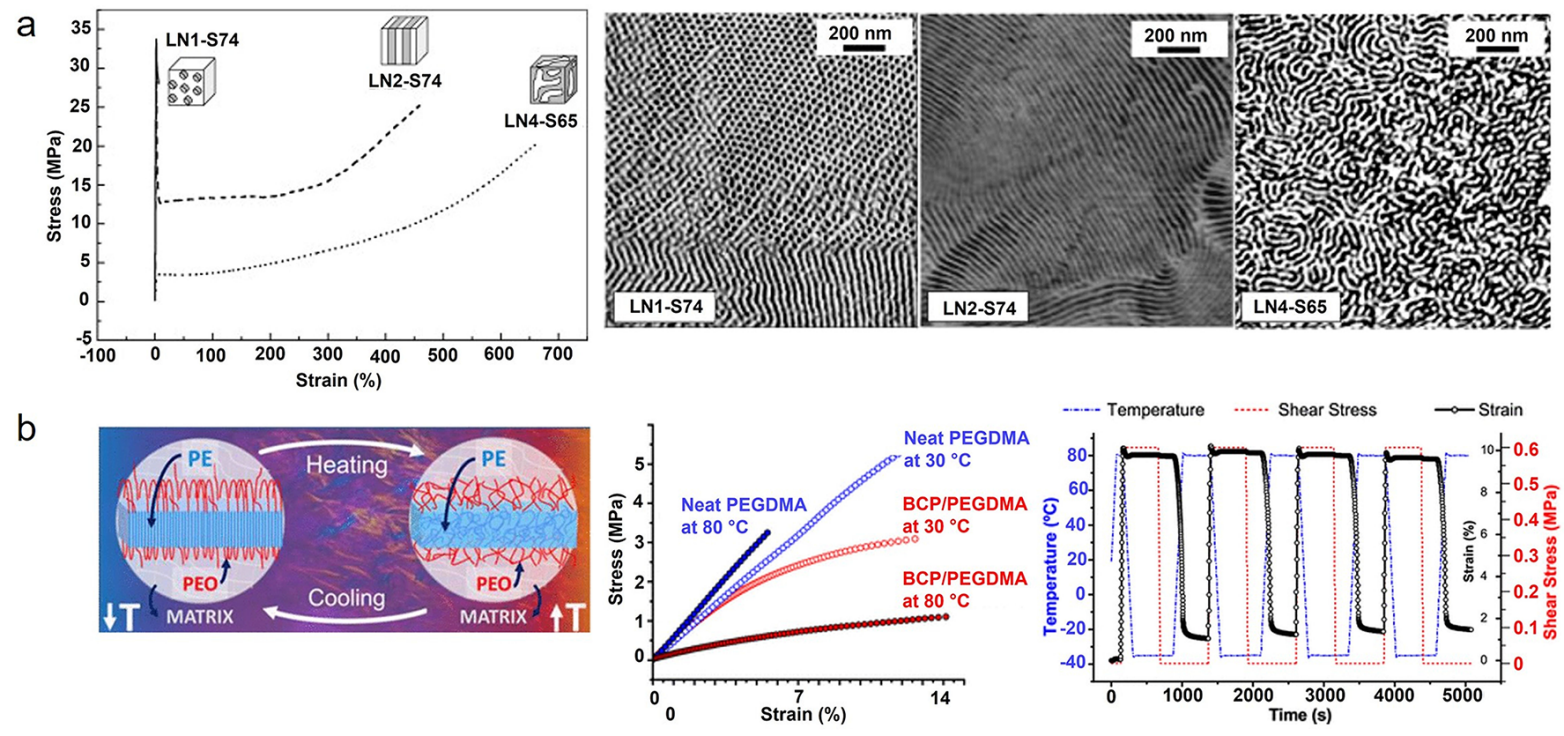
(a) Mechanical behavior of PS-*b*-PB block copolymers including symmetric polystyrene end blocks and sharp block transitions (LN1-S74), asymmetric polystyrene outer blocks with tapered interface with polybutadiene (LN2-S74), and random styrene/butadiene copolymer (LN4-S65), and their respective AFM phase images. Reproduced from ref (274). Copyright 2003 with permission from Elsevier. (b) investigation of block copolymer (BCP) assembly in a cross-linked PEGDMA matrix and its effect on mechanical properties at varying temperatures as well as cyclability of strain. Reproduced with permission from ref (268). Copyright 2023 American Chemical Society.

Block copolymers are covalently linked polymer chains which consist of homopolymers made from different monomers. They exhibit a wide range of mechanical properties directly depending on their compositions and nanostructures (Figure 25a). While the modular structure of this polymer class already suggests that many diverse types exist, we will introduce the concept through one of their most famous representatives, poly(styrene-*b*-butadiene-*b*-styrene) (SBS). In general, the mechanical behavior of SBS triblock copolymers under tensile stress can be classified into three groups. (i) *Rubber elastic behavior*: 20–40% polystyrene (PS) volume fraction gives a dispersed phase and homogeneous, rubber-like deformation under tension.^269,270^ (ii) *Ductile behavior*: With increasing symmetry in volume fractions of hard and soft phase, the system forms alternating layers (lamellas) of PS and polybutadiene (PB); macroscopic neck formation and drawing predominates during tensile deformation.^271,272^ (iii) *Brittle behavior*: 70–80% PS volume fraction (continuous phase); “transparent ductile thermoplastics” with inversed morphology.^22^ The yield stress increases while the elongation at break strongly decreases. The block copolymer breaks in a brittle manner (crazing).

Due to the widely separated glass transition temperatures (*T*_g_) of the constituents, a broad range of service temperatures is accessible. The ordered microphase-separated structures provide excellent mechanical properties (strength, stiffness, toughness, etc.) and sometimes optical properties (transparency).^270,273^ At ambient temperature, these materials behave like cross-linked rubbers since the flexible PB blocks (*T*_g_ ≈ −100 °C) are enshrined on either side by the PS blocks (*T*_g_ ≈ 100 °C), and thermoplastic processing is possible at higher temperatures.^270^ The lattice sizes of the microphase-separated block copolymer morphologies are usually within a range of 10–100 nm (Figure 25a).

The microphase-separated morphology of SBS triblock copolymers has already attracted the attention of the photopolymer community. For example, photocuring of the double bonds within the polybutadiene phase led to improved solvent and temperature resistance and improved adhesive strength of the material.^275−278^

Furthermore, photo-cross-linking experiments, using SBS triblock copolymers with varying vinyl contents in the polybutadiene phase and an acylphosphine oxide photoinitiator (Lucirin TPO), have been reported.^277^ It was shown that complete insolubilization requires the reaction of 17 double bonds per polymer chain, while the increase of the vinyl content from 8% to 59% in the PB phase did not show a significant influence on the cross-linking process, since it mainly enhances intramolecular reactions. Furthermore, both the reaction rate and the final degree of conversion could be increased by the addition of a bisphenol A diacrylate oligomer. In a subsequent publication, high-speed photo-cross-linking of SBS triblock copolymers in the presence of a trifunctional thiol cross-linker was described.^275,276^ Both the vinyl and the butene double bonds of the polybutadiene phase were found to react during the polymerization, as also confirmed in other studies.^279^ Another noteworthy contribution was the successful grafting reaction of a photoinitiator onto the SBS backbone with subsequent UV-cross-linking experiments.^280^ The gel fraction of the cured polymer could be adjusted by the grafting ratio and UV exposure time. The resulting materials were suggested for biomedical applications such as medical pressure-sensitive adhesives. These findings suggest that there is largely unexplored potential in the transfer of block copolymer heterogenization to the realm of photopolymers. Specifically, these results could already be the basis of photopolymerizable formulations in AMTs such as hot lithography, where the tolerance for resin viscosity is much improved. Processing such materials with AMTs would certainly enhance their attractiveness for biomedical applications. A first example in this regard has been published more recently, where butadiene rubber was added to a photopolymerizable maleimide/styrene formulation to mimic traditional ABS materials, which can be printed.^154^ In this case, the rubbery phase was linked with the photopolymer network covalently via its main chain double bonds. Another type of incorporation could be block copolymers, which are only incorporated into the network via their chain ends, which would facilitate chain slipping and reversible physical interactions before fracture (T1, T3). Furthermore, telechelic block copolymers with end functional reactive moieties that are able to undergo Diels–Alder based cross-linking reactions have been claimed.^281^ In another patent block copolymers have been claimed in combination with ring opening monomers, leading again to significantly tougher materials.^282^ A similar concept was realized with the previously introduced photoiniferters for radical/cationic hybrid systems, which give block copolymers with improved mechanical properties.^97^

Most recently, photopolymer networks have been investigated where the block copolymer polyethylene-*block*-poly(ethylene oxide) was incorporated into a methacrylate matrix without covalent attachment to the network to investigate the material’s crystallization behavior and effects on the mechanical response (Figure 25b).^268^ The matrix did not disturb the establishment of nanoribbons with a semicrystalline polyethylene core during heating–cooling cycles but confined the size of crystalline domains. Mechanical characterization revealed, however, that the glass transition became broader, and the material softened upon block copolymer incorporation. This is in line with the expectations based on our proposed strengthening mechanisms T1–T3. They are not applicable because there is no significant physical/chemical bonding interaction between the matrix and the block copolymer.

#### 6.2.3 Interpenetrating Networks

An interpenetrating network (IPN) is created by the simultaneous or sequential formation of at least two polymer networks with orthogonal chemistries.^283−285^ Since IPNs are a highly common approach to structuring photopolymers, an extensive review of all types of IPNs is beyond this Review. We refer interested readers to excellent and timely reviews.^113,286,287^ Herein, we will restrict the discussion to systematical differences regarding curing strategies and highlight particularly successful examples.

**Figure 26:**
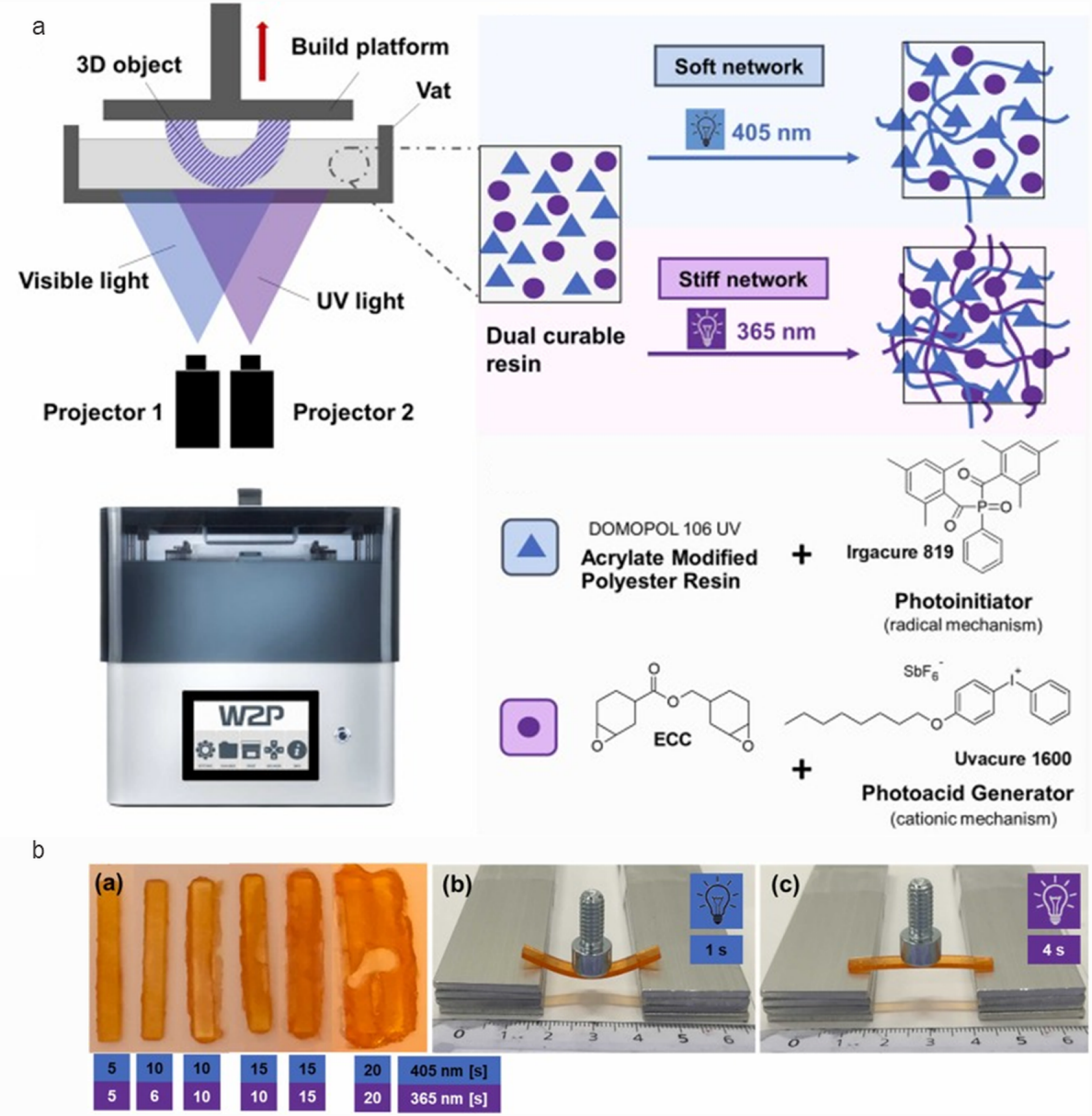
(a) Dual-color curing to manufacture IPNs via AM, utilizing a radical and a cationic monomer system for the soft and hard systems, respectively. (b) Demonstration of 3D-printing and the actuator behavior according to the irradiation conditions applied. Adapted with permission from ref (288). Copyright 2022 Elsevier.

The final IPN contains two polymer networks, which are completely separated from each other. Since the synthesis of such materials requires one-pot curing of two separate networks, the utilized active species for photopolymerization of each network must not react with each other. For example, this is the case when radical photopolymerization with radical initiators is combined with ionic photopolymerization with photoacid/base generators (Figure 26).^288^ Depending on the initiators’ reactivity upon light exposure, the networks may be cured simultaneously with a single light source, activating both initiators at once, or sequentially, requiring separate light sources to activate the initiators separately.^55^ The criterion for sequential curing, known as orthogonal curing, can be either sequence-dependent when one initiator reacts to light only in the UV range, while the other one reacts in the UV and visible light (vis) range, or sequence-independent, when the vis-active initiator does not react to UV-irradiation and vice versa.

In a way, an IPN architecture could be described as the analogous architecture for polymer networks, which we term polymer blends for thermoplastic (co)polymers. IPNs show superior mechanical properties such as creep and flow resistance, which found their use in coating, 3D-printing, and biomedical applications.^284^

The high level of physical entanglements between the networks results in IPNs exhibiting the sum of most of the characteristics of involved networks based on dual/multiple underlaying diverse chemistries for network formation. Reconciling contradicting mechanical properties of photopolymers within one material in this way has drawn much interest in increasing the toughness of photopolymers. Most prominently, the structural diversity of methacrylate monomers leading to hard but brittle networks and the established methods to lower the brittleness of epoxies via chain transfer reactions, which, however, are then softer, have been merged in developing high-strength IPNs.^44^ Critically, the incompatibility of the individual networks leads to the formation of small phase-separated domains starting at the segmental level, preventing the fulfillment of the crucial criterion that the networks are intertwined on the molecular level.

The concept of radical/cationic IPNs has further been enhanced by using a multifunctional alcohol as CTA. An ABS-like material with a tensile strength within the range of 30–65 MPa, a tensile elongation at break within the range from 2 to 110%, a notched Izod impact strength up to 6.4 J cm^–1^ and an HDT at 0.46 MPa within the range from 68 to 140 °C could be achieved.^289,290^

However, the photocuring of epoxies and acrylates in AM is generally associated with unfavorable side effects. Low curing rates have been reported since IPN curing is limited by the slower (usually nonradical) photopolymerization process, which limits printing speed. This has been counteracted with sequential curing procedures to exploit the best of both worlds, heterogenization via IPN formation and fast curing for AM.^291−293^ In the immediate presence of intact epoxy monomers, acrylates undergo cross-linking to rapidly form the green body of the desired specimen. Subsequent thermal curing of the epoxy then mitigates the interlayer defects and the aforementioned inefficiencies of pure (meth)acrylate networks. In this approach, green body stability as well as the interpenetration quality of the part are the most significant challenges.

#### 6.2.4 Photopolymerization Induced Phase Separation

Photopolymerization induced phase separation (PhIPS) is the most recent and arguably most important type of heterogenization strategy for photopolymers. This phenomenon delivers high rigidity contrast in the microstructure of photopolymers, allowing the domains in which the soft monomers are dominant to enlarge the plastic zones within the hard matrix and reduce the fracture-prone pathways.^44^

Phase separation is a thermodynamic phenomenon, though its establishment during synthesis is highly dependent on kinetics. For a thorough understanding of this phenomenon, the separation of an oil/water mixture can be utilized as a vivid example from daily life. Two insoluble components with different refractive indexes give rise to two distinguishable phases. The phases partly mix upon stirring but quickly separate when mixing ceases because the thermodynamic instability due to polarity differences is unfavorable and triggers reversion of mixing, for which there are no mobility restrictions to prevent the system from releasing its free energy.

Most common thermoplastic blends are also immiscible and phase-separable considering that the pure combination of transparent homopolymers usually results in hazy blends, although upon mixing at elevated temperatures, a lengthy solidification from the flowing state to the vitrified state gives phases considerable time to diffuse and integrate before the structure is completely stabilized. The development of phase separation in photocurable thermosets is intricate. While the diversity of formulation chemistries, as well as the dissimilar polymerization rates, may provide the prerequisites for PhIPS in initially miscible monomers/oligomers, the underlying cross-linking reactions followed by gelation tend to stabilize the structure before the diffusion reactions are accomplished and the system minimizes its free energy. Such trapped energies magnify shrinkage stresses and increase the tendency of the material to fail mostly by fracture. Early gelation may also severely slow down the diffusion rates, which can lead to phase stabilization at the nanoscale, thus preventing visible light scattering. Here it is essential to optimize the polymerization thermodynamics and kinetics to ensure that PhIPS effectively facilitates the desired heterogenized level, thereby enhancing the system’s overall performance.

From the thermodynamic point of view, if diffusion is possible, the positive values of free energy (Δ*G*_*mix*_) promote phase separation as the enthalpy of the reaction, i.e., the heat of mixing (Δ*H*_*mix*_), dominates the entropy (*T*Δ*S*_*mix*_) contribution:
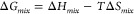
7

**Figure 27:**
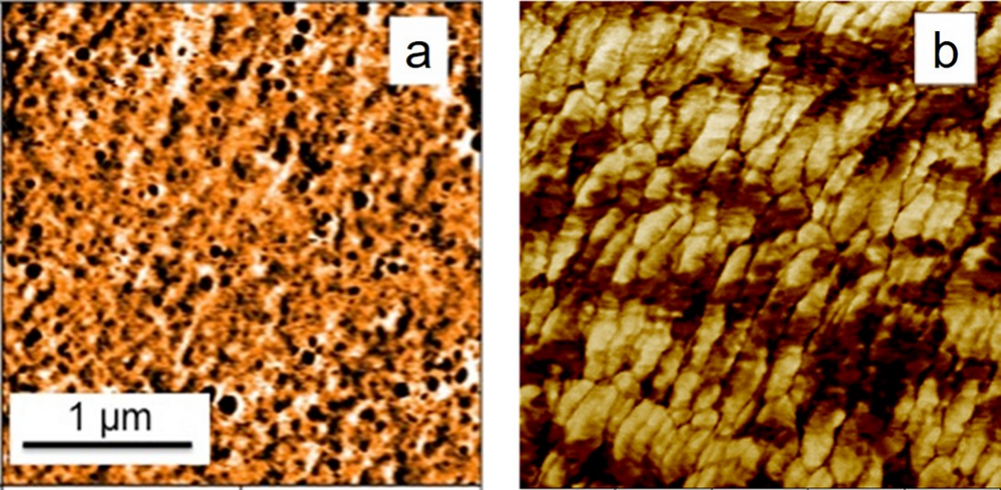
AFM examples of (a) droplet-like morphology resulting from the nucleation and growth mechanism of phase separation in photopolymers. Adapted with permission from ref (106). Copyright 2015 Elsevier. (b) Interconnected, stretched features resulting from spinodal decomposition mechanism of phase separation in a photopolymer (the dimension of the micrograph is 2 × 2 μm).

Phase separation may appear by one of two general mechanisms: (i) nucleation and growth (N&G), which typically occurs in a metastable state where the system requires energy input for separation and results in droplet-like morphologies and (ii) the spinodal decomposition (SD) mechanism, which occurs in highly unstable systems and results in composition fluctuations which lead to the formation of distinct phases. The latter is the most reported phase-separation pattern in radical and radical/cationic photopolymer systems.^106,126,145,294^ The transition of SD-dominated morphologies into N&G-dominated morphologies is generally possible by changing the processing parameters.^112,295^Figure 27 shows the morphologies achievable by both mechanisms.

Rocco et al. used the solubility parameter (δ_t_) to predict the phase separation and morphology in methacrylate-epoxide networks. This parameter is determined by considering the dispersion (d), polarity (p), and hydrogen bonding (h) contributions of the involved monomers:
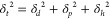
8

Phase separation is more likely when there is a greater difference between *δ_t_*s of involved monomers. The change of this parameter by varying the methacrylate/epoxy ratio was investigated as a tool to control the phase-separated morphologies in IPNs, and the findings were investigated using RT-FTIR, DMA, and AFM.^151^ PhIPS can also be evaluated by photorheometry as the gel point, determined as the crossover of G′/G″, significantly affects its occurrence.^143^

Besides the occurance of PhIPS in IPNs,^111,145^ PhIPS has been observed in semi-IPNs,^225^ photopolymers including thermoplastic prepolymers,^241,296^ inorganic materials,^241,296^ and polymerizable salts.^114^ Processing parameters, notably irradiation intensity, have a significant influence on the phase-separation mechanism and morphology.^106,126,241,295^ As an example, within a hybrid radical/cationic system, the morphology changes from a kinetically controlled continuous to a cocontinuous phase.^111,151^

**Figure 28:**
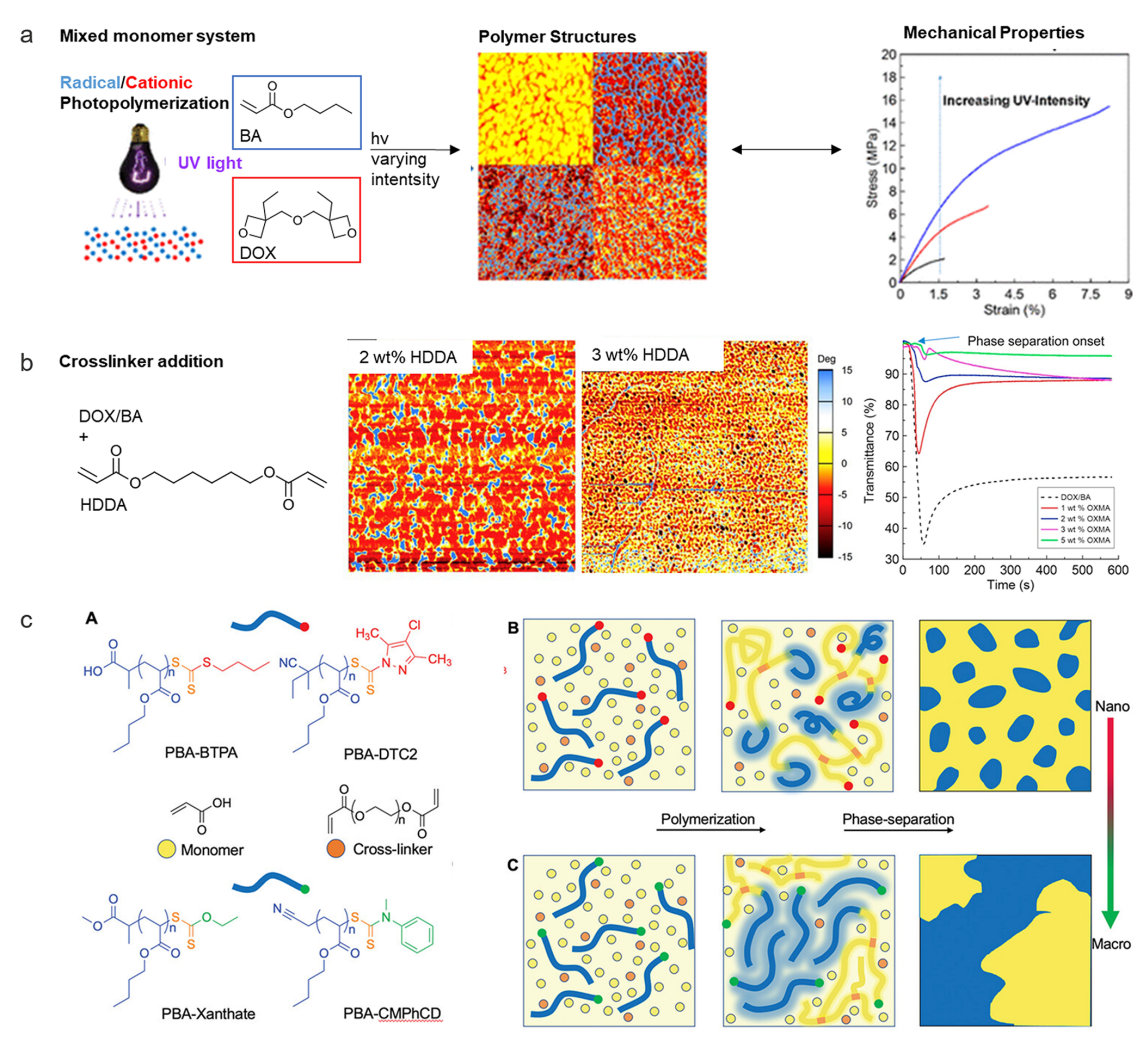
(a) Transition of an interpenetrating network of BA and DOX into PhIPS structures with varying mechanical properties depending on irradiation intensity. Reproduced with permission from ref (111). Copyright 2019 American Chemical Society. (b) PhIPS of the same system with varying amounts of HDDA cross-linker, which significantly influenced domain sizes as tracked via transmission measurement and AFM phase imaging. Reprinted with permission from ref (108). Copyright 2020 Elsevier. (c) Chemical structures of utilized monomers and chain transfer agents (A), which undergo phase separation by relying on the incompatibility of macromolecular chain transfer agents and multifunctional monomers (B, C).^95^

As mentioned, an IPN’s morphology can turn into phase-separated morphologies with completely different behavior. For example, cocontinuous hard/soft morphologies of two incompatible networks have been found most useful if the soft domains fall into the 50–500 nm range.^297^ Such morphologies were observed via AFM imaging in a series of studies on radical/cationic semi-IPNs including soft butyl acrylate (BA) and rigid difunctional epoxy (DOX, Figure 28a).^111^ The intensity of the irradiation was also shown to be highly effective for tailoring the morphology and qualities of interpenetration with 1/1 and 7/3 comonomer ratios (DOX/BA), but not of a 9/1 comonomer ratio, where phase separation could not be observed anymore. In phase-separable compositions, low intensities delayed the gel point to almost 115 s, which largely facilitated component diffusion and the formation of continuous soft phases with droplet-like hard phases included. Compared to homogeneous morphology, the resulting morphology showed lower strength and toughness. Cocontinuous hard/soft phases were obtained by overcoming this with higher irradiation intensities, which resulted in a 5-fold increase in elongation at break and a 40-fold increase in toughness.

Moreover, the presence and amount of cross-linkers influence the formation of PhIPS in IPNs at the nanoscale (Figure 28b).^108^ In a consecutive study, the morphology of 7/3 DOX/BA, as the optimized separable formulation of the original study, was manipulated by keeping the irradiation intensity constant and adding up to 5% 1,6-hexanediol diacrylate (HDDA) and 3-(ethyloxetane-3-yl)methyl acrylate (OXMA) as compatible cross-linkers of BA and DOX/BA systems, respectively. The findings showed that despite the significant suppression of photopolymerization induced phase separation by network gelation, the high tendency for separation still results in the development of continuous domains at the subnanometer scale. Furthermore, for 2–3 wt % HDDA and OXMA cross-linkers, considerable improvements in toughness and impact strength were achieved.

The impact of composition ratio and irradiation intensity have generally drawn the most interest in experimental studies.^292^ For example, more recently, modification of the component ratio in hybrid cationic/free radical IPNs was considered in controlling the kinetics of phase separation and its resulting morphologies.^145^ 3D-printable IPNs were developed from different ratios of the cationic comonomers, the monooxetane, 3-ethyl-3-[(2-ethylhexyloxy)methyl]oxetane (EHOX) and the diepoxide 3,4-epoxycyclohexylmethyl 3,4-epoxy-cyclohexanecarboxylate (EEC), photopolymerized along with the constant ratio of free radical (methacrylate) comonomers bisphenol A ethoxylated dimethacrylate (BisEMA) and hydroxyethyl methacrylate (HEMA). As the EEC/EHOX ratio increased, the polymerization rate of the cationic system approached that of the free radical system. This resulted in a shift in the IPNs’ morphologies from distinctly phase-separated to more interphase-containing structures. Importantly, the addition of EEC led to an increase in toughness.

The phase separations achieved in these early PhIPS investigations remained in the sub-micrometer range due to two effects: First, the low molecular weight of utilized monomers allows for comparably high miscibility of the components until a very late stage of the polymerization process. Second, the rapid polymerization kinetics of free radical photopolymerization cause early vitrification of the network and hence low diffusivity of the components, hindering phase separation. Decreasing irradiation intensity increased domain sizes toward the micrometer regime.^106,166^ However, the variability of this parameter is limited by the constraints for effective photoinitiation intensities.

Tuning PhIPS further, it has been shown that the addition of (unreactive) thermoplastic PMMA in photopolymerizable TEGDMA leads to phase separation, even at a low loading of 5%, while low molecular weight PMMA needs significantly higher loadings.^106^ Similar studies have been carried out using poly(butyl methacrylate) as a thermoplastic polymer.^109,112^ This underscores the importance of the formulation compounds’ molecular weights on PhIPS. In addition to monomer composition and irradiation intensity, other kinetics-related parameters including the type and content of photoinitiator^298^ and the sequence of cross-linking (for orthogonal thermal/photocuring systems)^299^ govern the PhIPS mechanism and morphology. For further insights on process–microstructure correlations, there are several reviews available, which specifically focus on this aspect.^300,301^

The use of macro-chain transfer agents (macroCTAs) leverages both high molecular weight formulation compounds and polymerization kinetics with delayed gelation and has thereby elevated the level of control on phase-separation behavior considerably. Such macroCTAs are initially soluble in acrylic resins but become insoluble as RAFT photopolymerization progresses.^301^ The oligomeric nature of the CTA stimulates phase separation at an early stage of the polymerization process. Furthermore, efficient RAFT kinetics delayed the gel point effectively, allowing the components to diffuse until later stages of the polymerization process and hence enhancing phase separation. Thus, by simply tuning the ratio of macroCTA to monomer mix, various microstructures were achieved (Figure 28c).^95^ Furthermore, tuning the chain transfer kinetics via the Z-group of the macroCTA influenced PhIPS behavior. With longer, more efficient macroCTAs, the gel point was shifted to later stages, increasing the domain sizes. Star-shaped macroCTAs further enlarged accessible microstructures and enabled precision control of nanoscale phase-separation architecture.^123^ This PhIPS approach via macroCTAs has also been demonstrated to be feasible for additive manufacturing.^302,303^

**Figure 29:**
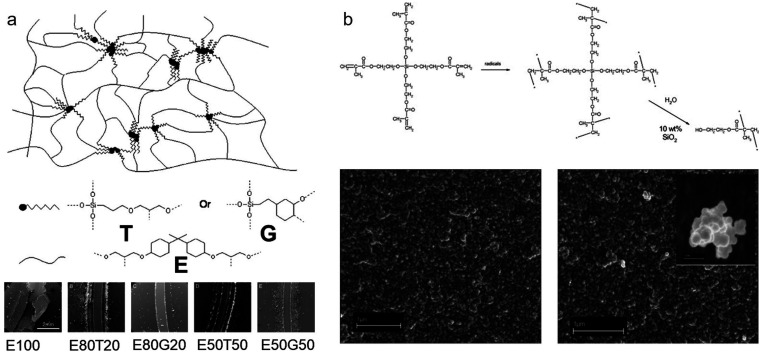
(a) Scratch tests for PhIPS of epoxide-based photopolymer coatings with varying siloxane content and epoxide matrix upon single-step curing via cationic and sol–gel reaction. Reproduced with permission from ref (304). Copyright 2010 Wiley. (b) Sol–gel process of tetrafunctional silane monomer tetrakis[(methacryloyloxy)ethoxy]silane in a methacrylate/epoxy IPN. SEM images before (left) and after (right) the second step of two-step curing via radical photopolymerization. The release of monomers for the sol–gel process via photoacid generator resulted in photopolymerization-induced phase separation (PhIPS) through SiO_2_ formation from a siloxane-containing acrylate network. Adapted with permission from ref (306). Copyright 2012 Elsevier.

While the previously introduced PhIPS examples utilize the curing of two incompatible networks from the monomers in a formulation, there is also the possibility to cure a single network first and then release the monomers for the second, incompatible network from the cured network. For example, photopolymerizable block copolymers with telechelic end groups and extremely low vapor pressure have been claimed to undergo PhIPS.^281^ Further work has been done in the field of organic–inorganic hybrid networks based on alkoxy-silane-containing acrylic resins (Figure 29a).^304^ Furthermore, a mixed system of acrylates and epoxides was polymerized via radical and cationic curing, containing alkoxysilanes, which were activated with the cationic photoinitiator simultaneously.^305^ In another instance, a polymerizable tetra-acrylate silicate monomer was added to a methacrylate/epoxy IPN with subsequent water-vapor-aided SiO_2_ release from the silicate-containing monomer (Figure 29b).^306^ A significant reinforcing effect has been found by nanosilica particles, which were formed in situ.

Achieved phase separations may effectively enhance mechanical properties such as yield strength, toughness, and creep. From the fracture mechanical point of view, PhIPS can deliver a broad range of rigidities solely dependent on the microstructure, allowing the domains, in which the soft monomers are dominant, to serve as plastic zones within the hard matrix and reduce fracture-prone sites.^44^ PhIPS can also dissipate shrinkage stress due to the formation of plastic zones.^107,237,238^ Up to 80% shrinkage stress reduction has been reported for this approach.^307^ For many materials, however, the size of the plastic zone after PhIPS remains insufficient to improve toughness effectively. This is mainly due to the suppressive effect of early gelation on PhIPS, despite the high thermodynamic tendency of systems to undergo phase separation.^50^ Decreasing the polymerization rate to postpone the gel point through manipulating the processing parameters (e.g., reducing light intensity) and formulation (e.g., lowering photoinitiator content, replacing the cross-linking monomers with reactive diluents), although to some extent advantageous, is associated with many technical and performance-related limitations.^107^

## 7 Applications

The application of heterogenization in the polymer industry is virtually limitless. Here, we will only emphasize prominent examples without claiming completeness. Apart from the significant effect of improved toughening on usability in different industries (aerospace, automotive, medicine, etc.), heterogenization enables tailoring specific photopolymer characteristics, including optical^250^ and electrical properties,^308^ data storage,^309^ electromagnetic absorption,^800^ and shape memory.^114,310^ This further emphasizes the importance of heterogenization in jewelry and decoration, dentistry, electronics, and robotics applications.^5,218,311^

**Figure 30:**
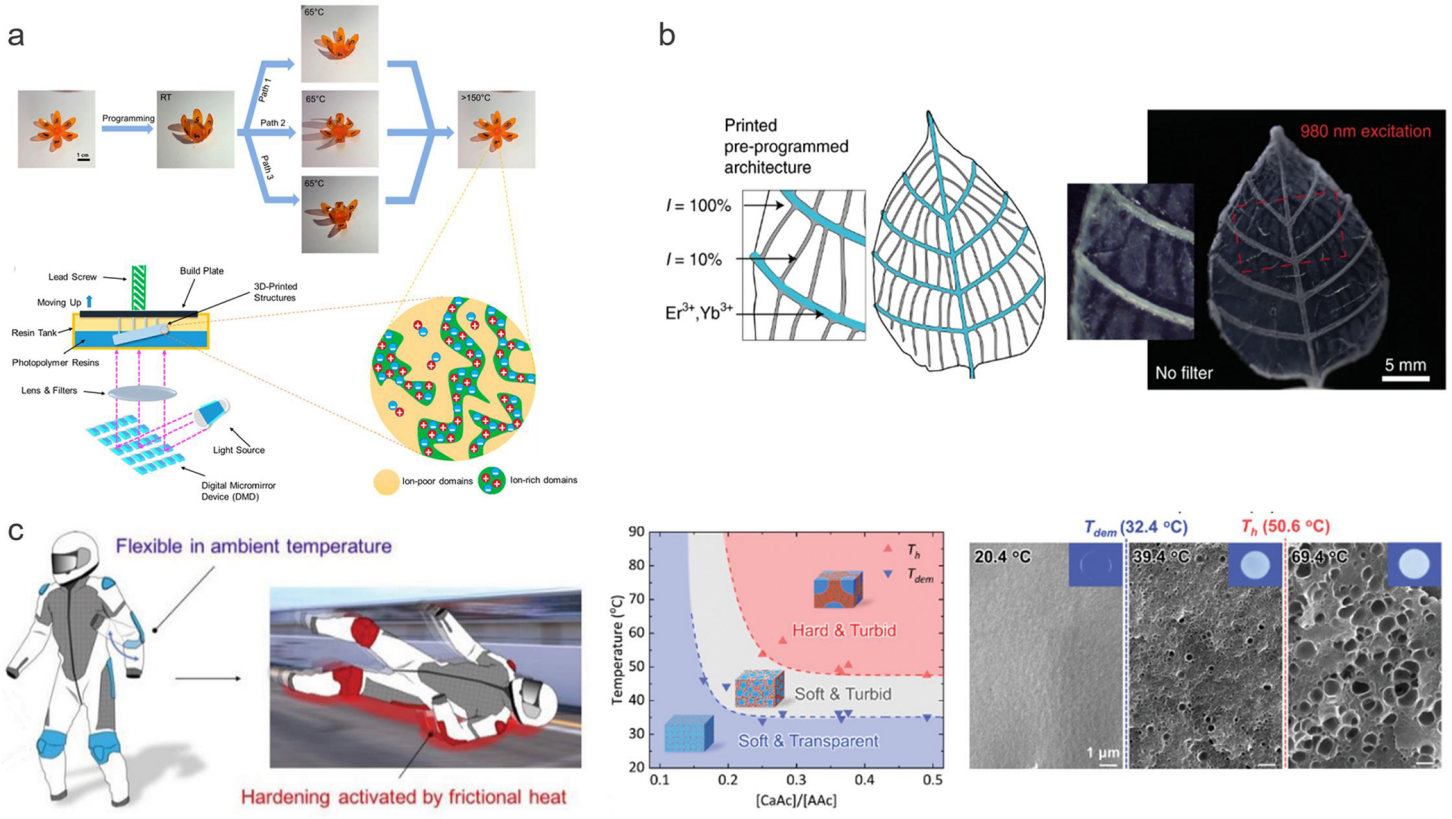
(a) Triple shape memory phase-separated DLP printed photopolymers. Adapted with permission from ref (114). Copyright 2019 American Chemical Society. (b) Different levels of transparency in primary and secondary leaf veins composed of phase-separable resins by adjusting the irradiation intensity of 3D-printing. Reprinted with permission from ref (296). Copyright 2011 John Wiley and Sons. (c) Body protection activated by friction heat as a potential application of phase separation, phase diagram of reversible thermal hardening in poly(acrylic acid)–calcium acetate at elevated temperatures, and SEM images of the phase-separated microstructures quenched at 20.4, 39.4 and 69.4 °C. Reprinted with permission from ref (314). Copyright 2020 John Wiley and Sons.

Stimuli-responsive polymers are a prominent example of advanced materials that can change their properties or behavior in response to specific external conditions or triggers.^312^ These polymers have vastly been investigated for 4D printing in recent years.^313^ For example, PhIPS has shown the potential to deliver the temporary and permanent network architectures required for such structures.^311^ Using a conventional DLP printer, an acrylic resin formulation containing isobornyl acrylate (IBOA)/2-ethylhexyl acrylate (EHA)/poly(ethylene glycol) dimethacrylate (PEGDMA) and vinylbenzyltrioctylphosphonium 4-styrenesulfonate (VBTOB-SS) as ion pair comonomers was printed.^114^ The resulting shape memory structures delivered phase-separated morphologies including ion-rich and ion-poor domains, which kept high strength over a wide temperature range (Figure 30a). Another example is AM of complex and high-resolution glass parts. A resin was separated into different phases to deliver different levels of transparency (Figure 30b).^296^ In one case, the belief that heating always softens polymers was challenged by the development of phase-separable rubbery polymers. These polymers stay soft at moderately elevated temperatures due to small hard domains. However, phase-separation tendencies increase upon further heating, causing the hard domains to grow and become the dominant factor for the material’s behavior (Figure 30c). This was once again inspired by biological organisms that, when exposed to heat, exhibit glassy behavior.^314^

As photopolymer heterogenization progresses, we may expect more diverse and novel applications. From personalized medical implants to smart materials and rapid 3D-printing, photopolymers can potentially revolutionize industries. Additionally, with a growing focus on sustainability, photopolymers may contribute to developing eco-friendly products and processes, pushing the boundaries of technology and design.^315,316^

## 8 Putting Reviewed Heterogenization Approaches in Perspective

On a case-by-case basis, the introduced heterogenized materials reviewed herein in general and those related to photopolymers specifically have been proven to exhibit superior thermomechanical performance compared to their traditional amorphous reference photopolymers. Thus, the concepts are highly suitable for developing next-generation photopolymers in applications of three-dimensional parts, which have been unlocked with the rise of light-based AM. However, analyzing the degree of improvement by photopolymer heterogenization proves to be highly complex due to two main reasons.

**Figure 31:**
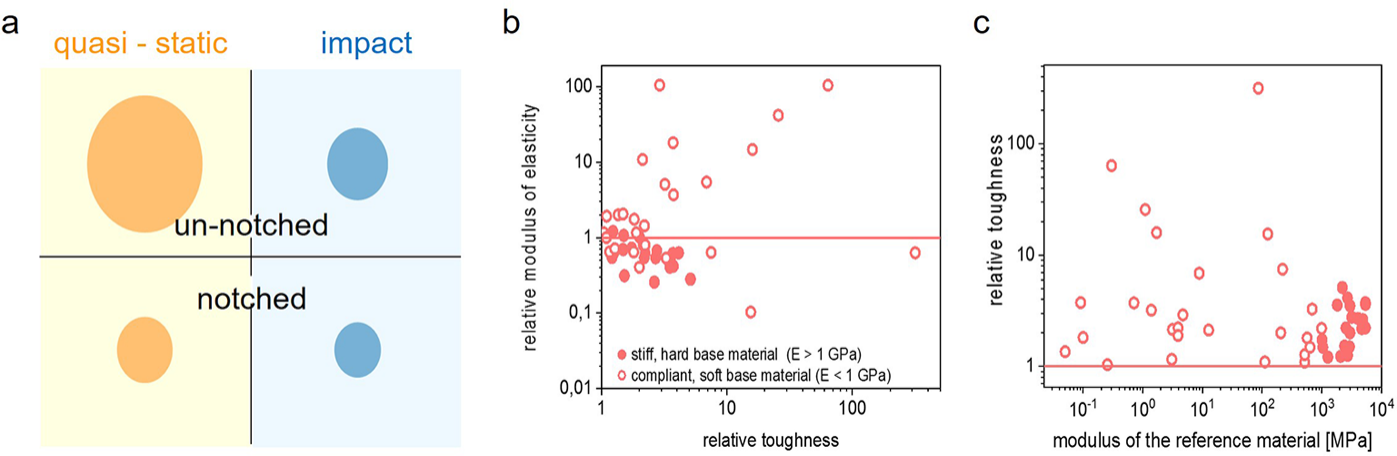
Results of exploring the trade-off of toughness and the modulus of elasticity in more than 70 related studies: (a) applied test conditions to evaluate toughness in photopolymers, (b) accompanying change of modulus with toughness enhancement, and (c) toughness improvement versus modulus of elasticity of the base material. Further details are provided in Figure S3.

First, the reported data for thermomechanical properties of such materials do not adhere to a single standard. This tendency is particularly pronounced in the area of photopolymerization heterogenization because the varying types of heterogenization strategies are situated in different research communities with very different focuses regarding outcomes (e.g., interest in polymerization mechanisms vs interest in applications). Related to this, reporting of obtained thermomechanical improvements relative to a reference material is not standardized either. A prime example is the comparison of impact testing or fracture mechanics values, the gold standards to measure a material’s toughness. Only a relatively small number of contributions apply sharp notches to their testing samples and assess their toughness by fracture mechanics testing methods.^124,317−323^ Furthermore, an overwhelming majority of tests are done at room temperature, where a swift decline of the modulus due to a broad glass transition temperature range starting just above room temperature is not accounted for. A short overview of the applied testing conditions is given in Figure 31a, indicating that in the field of photopolymer development, tensile testing of un-notched specimens at low speeds is favored for toughness quantification. On the one hand, this is a consequence of the high number of works dealing with soft and very flexible photopolymers, but on the other hand, this preference is also found in the field of stiff photopolymers. In principle, the issue may be simplified by arguing that surface and bulk imperfections (e.g., bubbles, shrinking cracks, surface defects, etc.) serve as inherent notches within the specimens. However, the collective results indicate that apart from the material’s intrinsic characteristics that determine toughness, the quality of sample preparation is highly decisive. Herein, inserting a defined notch would lead to a clear shift in the contribution of material properties to thermomechanical behavior because the inserted notch will “overrule” the imperfections. Approaching the stadium of commercialization, serial production, or quality control of stiff photopolymers, the amount of impact testing of notched and un-notched specimens or even semifinished products increases. The same is observed for elastomer-like photopolymers with increasing amounts of tear tests.

Second, the amount of data available in some heterogenization strategies (e.g., composites) is incomprehensibly high, while data for other heterogenization strategies (e.g., block copolymers) is highly underrepresented. However, in the following, we attempt to harmonize examples of available data in the best possible way to paint a more complete picture of the impact of photopolymer heterogenization on thermomechanical performance, particularly toughness.

To do so, we will first have to settle for a suitable definition of toughness enhancement. An important attribute of toughness enhancement, when a second material is added to a matrix material (also termed base material, a term which can also be used more broadly for a material mixture without phase separation) for toughening, is related to its trade-off with stiffness. Toughening of a brittle matrix material through the addition of a softer material is frequently accompanied by a clear reduction in stiffness and strength. However, in the ideal scenario of improving toughness, stiffness (and strength) remains on a comparable level before and after the inclusion of the soft material.

Therefore, in Figure 31b and c we show the changes of modulus of elasticity and toughness for materials analyzed in this Review, which consist of either a hard or soft matrix doped with either a soft or hard material, respectively.At this point it is also important to note that the previously mentioned softening effect during toughening is less important for soft elastomer-like photopolymers as emphasis often lies on the adjustment of stiffness while maintaining high toughness. Starting with a very soft base material, toughness enhancement by tailoring network structure or inserting hard phases will unsurprisingly increase the stiffness. To make this correlation visible for the dataset analysed herein, the relative toughness has been explored as a function of the absolute modulus of elasticity of the corresponding unmodified matrix-only referenced photopolymers. Figure 31c represents the statistical findings, which cover both quasi-static and impact tests. If the material was mixed from two components over the full composition range from 0 to 100%, the modulus of the stiffer component was chosen as the reference. In some cases, the storage modulus value (E′) is utilized. If shear modulus values were stated in the respective reference, these were multiplied by a factor of 3 to obtain an approximation of E′. Extensive results as well as the corresponding references for each data point can be found in the Supporting Information.

Unsurprisingly, this analysis confirms that several attempts to toughen materials also lead to significant softening. However, an impressive amount of work has led to significant toughening up to a factor of 2 and, in exceptional cases, up to >10^124^ and even 64^324^ without a corresponding decrease in modulus. While such extreme examples are still rare and typically found for soft photopolymers, our analysis confirms that the reviewed concepts for photopolymer heterogenization have already demonstrated significant progress with respect to the thermomechanical improvement of photopolymers for 3D parts. Heterogenization seems to have the potential to aim for even better materials than those of the current state of the art.

**Figure 32:**
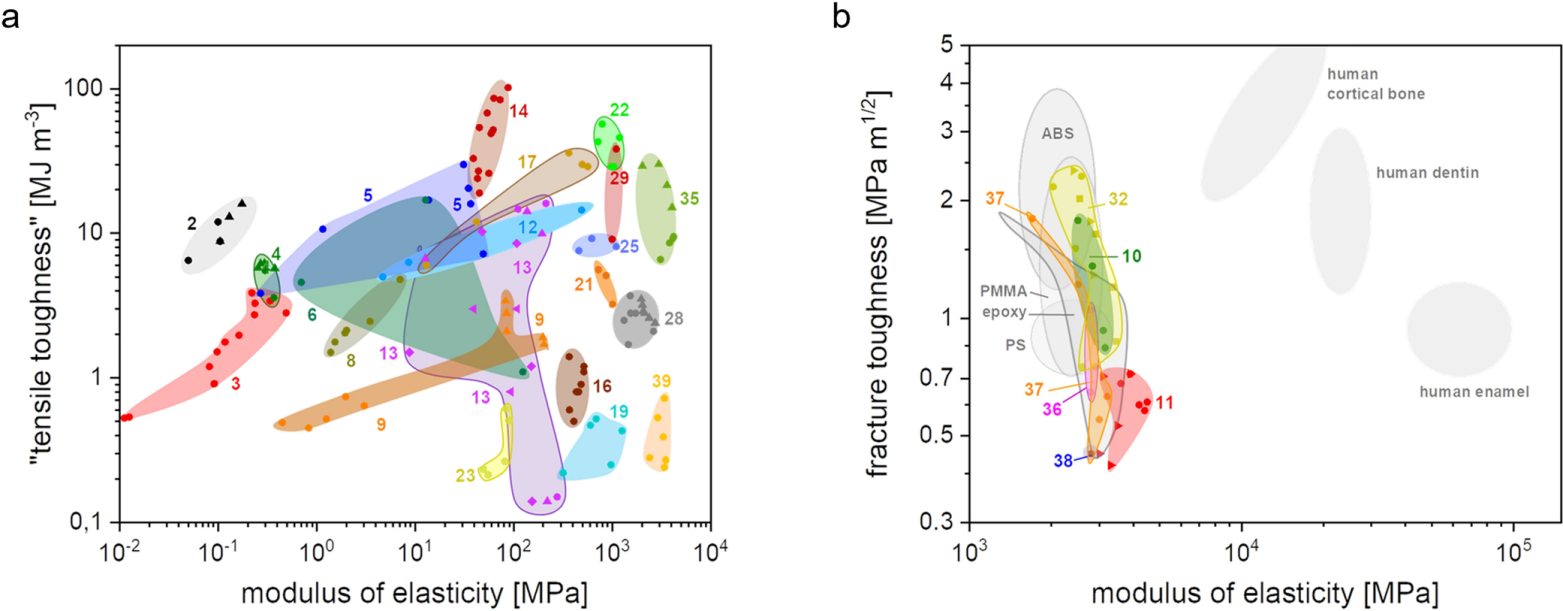
Toughness vs modulus of elasticity for reviewed heterogeneous photopolymers at room temperature: (a) so-called tensile toughness and b) fracture toughness at quasi-static loading, sourced from refs S2−S39 in the Supporting Information. In (b) the property ranges of some thermoplastics, thermally cured epoxy, and biomaterials are plotted in gray color for comparison. See more details in the Supporting Information. Remarks: (I) The applied strain rates during testing vary between the cited works, so some shifting of the results would occur if every material were measured under the same testing conditions. (II) Whereas the areas of the photopolymers and the epoxies in (b) directly follow the results from single values given in the literature, the symmetric areas of the reference materials in grey represent simplified ranges for toughness and stiffness vertically and horizontally as it is typically done in Ashby plots (see Figure 17).

An even more detailed analysis via Ashby plots, where the heterogenization approaches of each analyzed reference are depicted separately, gives even more insight into their current potency and future potential (Figure 32).

Due to the previously mentioned differences in reporting, most data are reported in an Ashby plot relating tensile toughness to elasticity (Figure 32a), while data for which fracture toughness via notched specimens has been determined are included in a separate Ashby plot (Figure 32b). It can be seen that the stiffness and toughness values span over several orders of magnitude, which offers the possibility to select 3D-printable photopolymer systems with appropriate base mechanical properties for demanding applications. Finally, fracture toughness values of typical reference materials discussed in section 5.4 have been added as gray areas to demonstrate the success of heterogenization. While heterogeneous photopolymers cannot yet reach the performance of biomaterials, which exhibit highly precise hierarchical structuring across several length scales, they have outperformed typical methacrylic polymers and epoxies. Most impressively, fracture toughness values competitive with traditional heterogeneous polymers such as ABS could already be reached.

## 9 Outlook

The comprehensive body of work reviewed herein, and in particular our culmination of this work in the previous section, impressively demonstrate the power of heterogenization strategies to evolve photopolymers from brittle coatings to robust 3D soft matter parts with versatile functionalities. While the physical heterogenization of photopolymers (fillers, inkjet printing) has been explored excessively, much less literature is available on the chemical heterogenization of photopolymers (semicrystallinity, block copolymers, IPNs, PhIPS), a more subtle yet promising and recently flourishing strategy for heterogenization.

Although adding fillers to photopolymer matrixes is one of the most classical approaches to modifying their properties, this research area remains highly relevant. However, their processability (viscosity, scattering, absorption, resolution) remains a big challenge. Due to (optimized) optical properties and printing resolution, nanofillers are often favored for additive manufacturing. However, toughening with such systems gave the most impressive improvements for reinforcing soft, flexible materials. In the case of hard, stiff materials, their influence is often marginal. Nonetheless, the functional properties of the fillers have to be the focus of toughening strategies in both cases. In the future, nonconventional filler properties like color or self-illumination should be used to influence the curing reaction and to influence the mechanical properties. We also anticipate the combination of filler alignment and AM to become a further focus in future research objectives, which can be realized by local spontaneous interactions (self-assembly), printing-induced forces (linear oscillator, journal bearing laminar flow), or external fields (magnetic, electric, acoustic).^325^ Especially favorable alignment of high aspect ratio fillers with respect to loading directions could lead to larger improvements in the toughness of 3D-printed parts. Here, the scale-up regarding dimensions and throughput will be a challenge in particular. Taking sustainability into account, fully degradable or easily reusable fillers should become of interest.

The presented innovative advances in inkjet printing demonstrate its potential to achieve more complex structural photopolymers. The high accuracy of inkjet printing also aids the elucidation of the effect of heterogeneities on the mechanical properties of multicomponent photopolymers and the mimicry of high-strength biomaterials. However, to maximize inkjet printing utility, the range of high-resolution printable inks needs extension. So far, only a few studies focus on the challenges of currently available inks, i.e., rheological limitations, physical characteristics, and oxygen inhibition effects, which significantly affect the use of inkjet printing. Moreover, limited printing scalability implies the importance of using inkjet printing in hybrid printing systems. In such systems, concerns regarding the diffusion of ink into the printed substrate require further research. It could cause deviations from the expected shapes and impact the mechanical properties of the final material.

Microstructure–property relationships in IPNs have been studied extensively for the combination of epoxies with (meth)acrylates. Tailoring the mechanical properties, particularly fracture toughness, was mostly followed by adjusting the composition ratio of the involved monomers. However, the effect of other parameters, such as processing conditions and the sequence of network formation, have been observed to be highly relevant for the final microstructures and have been investigated far less to date. While the IPN networks contribute independently and provide a “blend” character, the direct effect of network interactions, such as hydrogen bonding, and PhIPS on mechanical properties remain only scarcely characterized and require deeper exploration. Additionally, process optimization to achieve the highest printing quality along with the highest monomer conversion within the IPN structure are the key areas for IPN development in AM.

Semicrystalline photopolymers have been particularly successful as thermoplastic and thermoset thiol-ene polymers.^266^ However, the body of work beyond this photopolymer class remains limited to date, particularly for main-chain crystallinity. While ring-opening polymerizations have often also been explored for photoinduced polymerizations, their utilization as bulk materials, particularly for 3D-printing, remained elusive until very recently. Our group has demonstrated a range of cationic and anionic ring-opening polymerizations for the light-induced 3D-printing of pure polyesters and polycarbonates.^29−1001,326^ Yet, the degree to which crystallinity can be trapped in covalent photopolymer networks remains a challenge for photopolymer chemists.

Block copolymers are currently largely unexplored for their incorporation in photopolymers.^327^ First efforts where block copolymers have been included during PhIPS or as additives,^328^ however, demonstrate their large potential. Only simple changes in the block copolymer already have a massive influence on microphase separation. Therefore, we suggest further translating the tremendous knowledge from classical block copolymer research to obtain superior heterogeneous photopolymers.

Several very recent advances in the realm of photopolymerization induced phase separation, some of which have also been translated to additive manufacturing, have demonstrated how much untapped potential lies within this curing strategy. We expect the body of PhIPS-related work to grow rapidly with a strong focus on ever-more sophisticated nano- and microstructures and suggestions for applications thereof. For example, the interconnectedness of 3D-printed PhIPS structures has been utilized to print load-bearing ion-conductive parts.^302,303^ While the creative application of these new nano- and microstructures will be highly interesting for the materials community, we would like to emphasize the importance of parallel conceptual development of PhIPS since such studies will broaden the PhIPS-accessible materials range beyond the state-of-the-art. For example, the combination of PhIPS enhancement via macromolecular formulation components and kinetics through the use of macroCTAs for RAFT photopolymerization has increased the control over microstructures significantly. Only the realization of the underlying fundamental effects causing PhIPS has allowed the development of this concept.^301^

Finally, we suggest utilizing the tremendous number of findings available for heterogeneous thermoplastic polymers in general and translating successful strategies to the realm of photopolymers. Successful translation, however, is only possible if we approach this task interdisciplinarily with chemists supplying new monomers and polymerization strategies to enlarge the range of accessible microstructures, material scientists and physicists analyzing the impact of various microstructures on material behavior, and engineers supplying new AMTs. Tightly knit collaborations between all disciplines as well as trans-disciplinary interests of researchers will lead to synergies and cross-fertilization between these three key research areas and thus accelerated progress in the field of heterogeneous photopolymers.

## Supporting Information Available

Schematic sketches of typical toughness-enhancing mechanisms in polymers and examples of toughness enhancement related to modulus and its change upon heterogenization in photopolymers reported in the literature. Data compilation of change in modulus with toughness enhancement as reported in literature (relative and absolute), including references from which the data has been taken (PDF)
List of abbreviations used in this paper (PDF)
