# Supplementary material for: From Unregulated Networks to Designed Microstructures: Introducing Heterogeneity at Different Length Scales in Photopolymers for Additive Manufacturing
Source: Chem Rev. 2024 Mar 28;124(7):3978–4020. doi: 10.1021/acs.chemrev.3c00570 (PMC11009961; doi:10.1021/acs.chemrev.3c00570)
Supplement: Supplementary file 1 — cr3c00570_si_001.pdf [file cr3c00570_si_001.pdf]

Supporting Information

From unregulated networks to designed microstructures: Introducing heterogeneity at different length scales in photopolymers for additive manufacturing

Mojtaba Ahmadi,[1,§] Katharina Ehrmann,[2,§,*] Thomas Koch,[1] Robert Liska,[2] Jürgen Stampfl[1,*]

[1] Institute of Materials Science and Technology, Technische Universität Wien, Gumpendorfer Strasse 7, 1060 Vienna, Austria

[2] Institute for Applied Synthetic Chemistry, Technische Universität Wien, Getreidemarkt 9/163, 1060 Vienna, Austria

[§] These authors contributed equally.

* Corresponding Authors
Email: katharina.ehrmann@tuwien.ac.at, juergen.stampfl@tuwien.ac.at

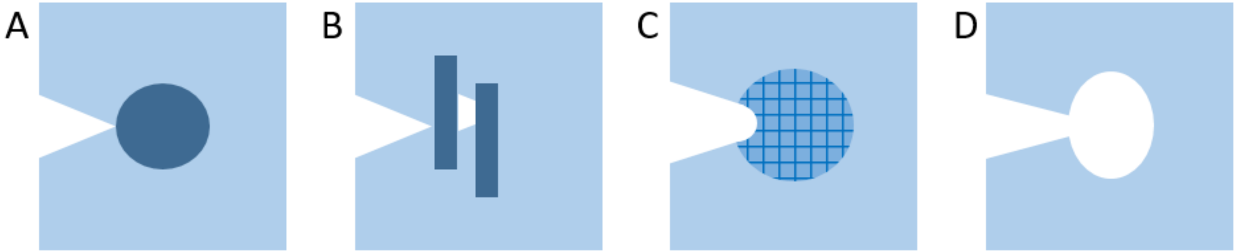

Figure S1: Crack stop at **A** rigid particles, **B** fibers, **C** soft particles and **D** voids. Adapted from [S1]

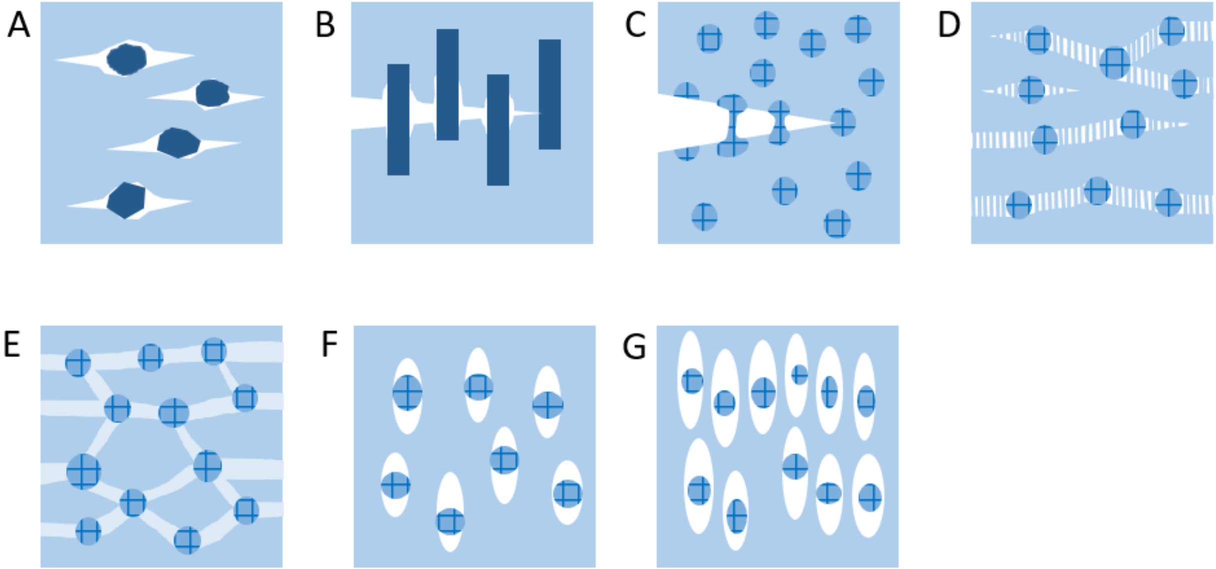

Figure S2: Local micromechanical effects leading to energy dissipation and enhancement of toughness. **A - B** Multiple microcrack and void formation, **C** bridging, **D** multiple craze initiation, **E** multiple shear band initiation, **F** debonding with void generation and **G** yielding of the matrix ligaments. Adapted from [S1].

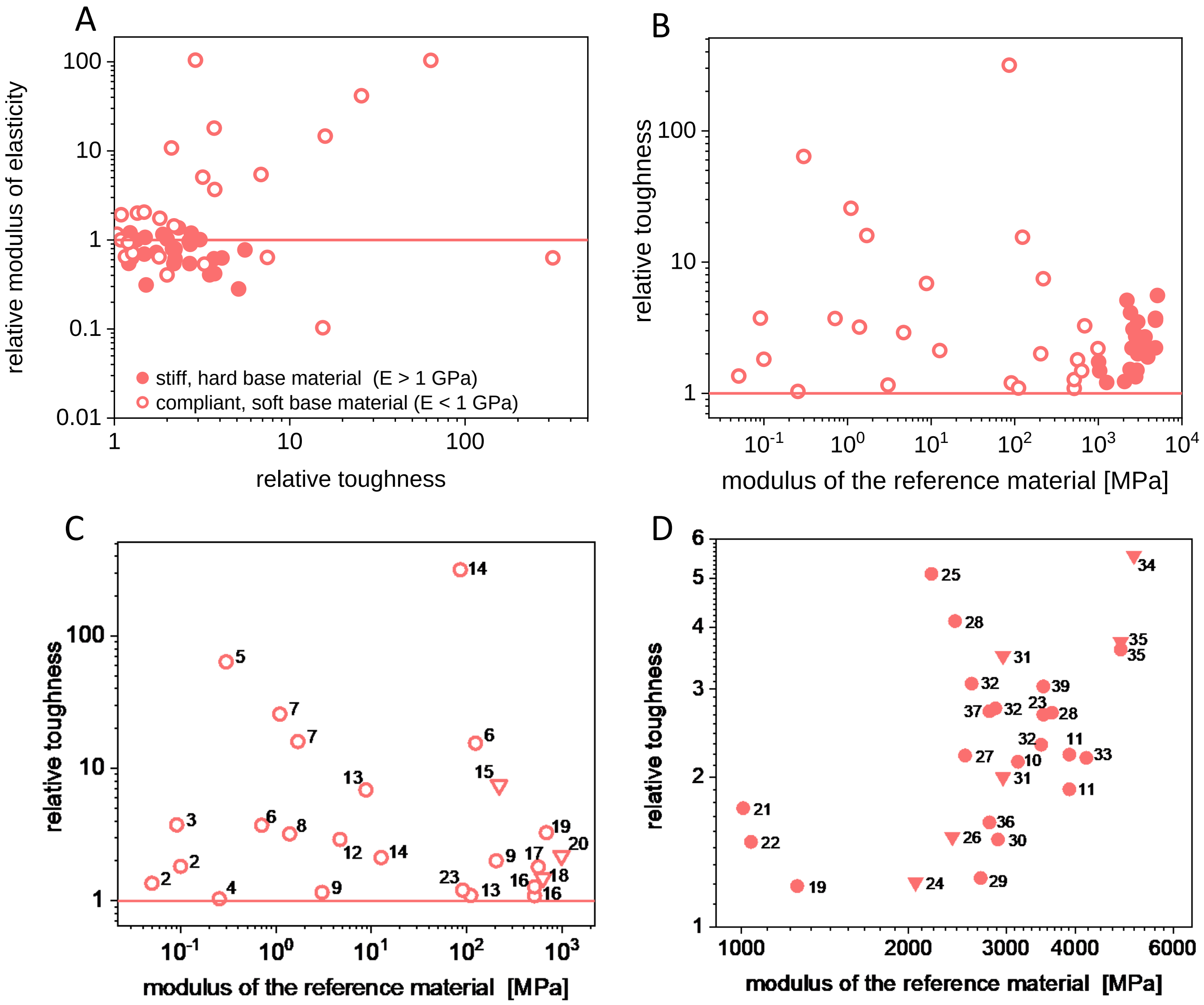

Figure S3: A The accompanying change of modulus with toughness enhancement. Values taken from selected references [S2-S35]. In most cases the stiffer and harder materials become more compliant if their toughness increases. **B** Relative toughness increase after modification vs. the modulus of elasticity of the respective unmodified base or reference material. If a material was mixed from two components over the full concentration range, the modulus of the stiffer material was chosen as reference. **C** Detail of B: lower modulus range. **D** Detail of B: higher modulus range. The numbers indicate the referenced literature [S2-S39], the circles represent quasi-static tests, the triangles represent impact tests.

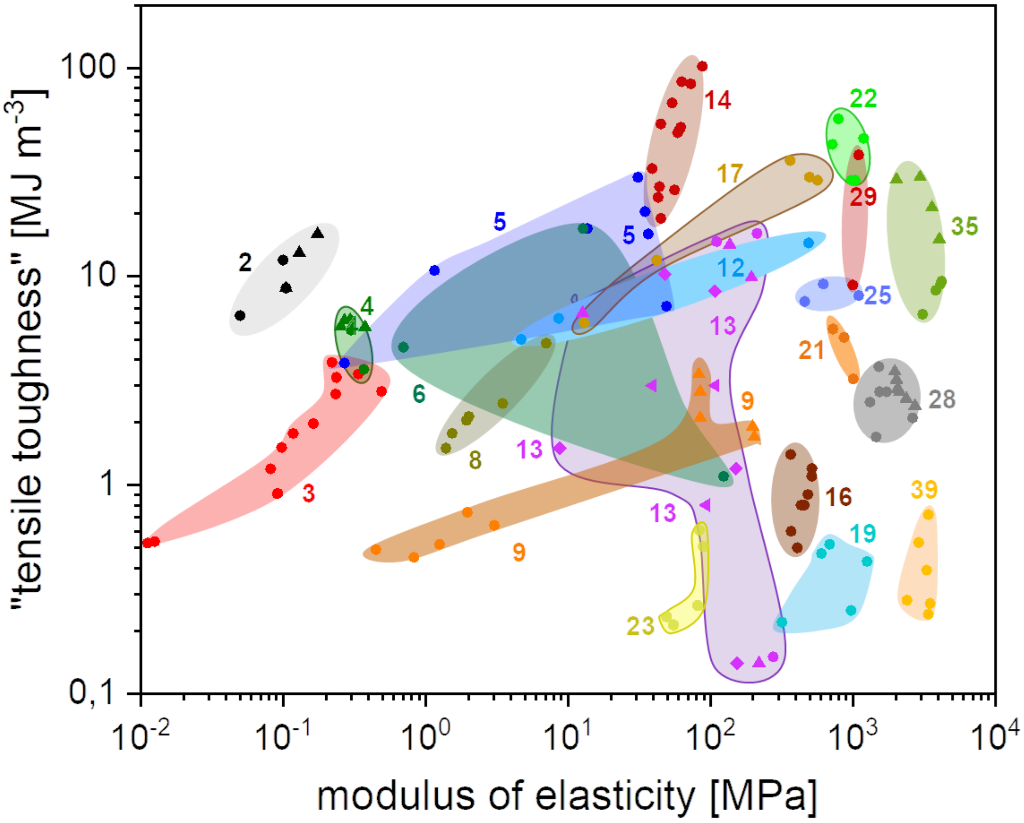

Figure S4: So-called tensile toughness vs. modulus of elasticity of various photopolymer systems. Values taken from selected references [S2-S39].

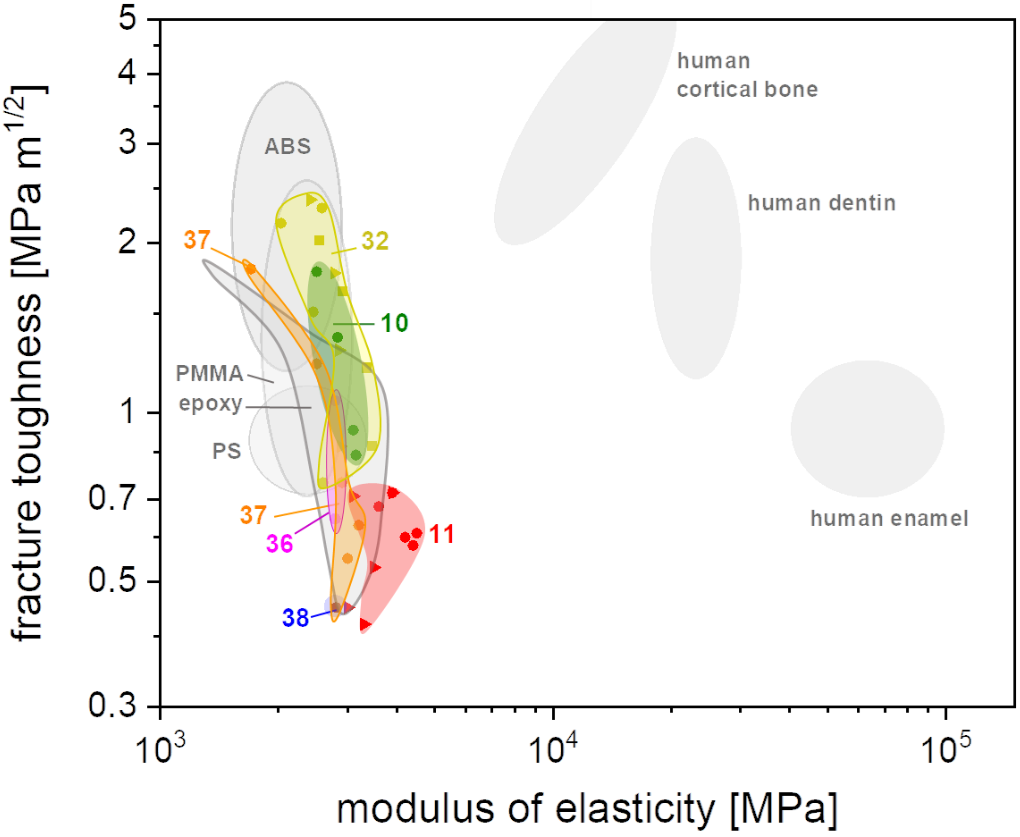

Figure S5: (Quasi-static) fracture toughness vs. modulus of elasticity of various photopolymer systems. Values taken from selected works [S2-S39]. Some thermoplastics, thermally cured epoxies and biomaterials are plotted in grey color for comparison.

References

(S1) Michler, G. H.; Balta-Calleja, F. J. *Nano-and micromechanics of polymers: structure modification and improvement of properties*; Carl Hanser Verlag GmbH Co KG, 2012.

(S2) Lai, P.-C.; Ren, Z.-F.; Yu, S.-S. Thermally Induced Gelation of Cellulose Nanocrystals in Deep Eutectic Solvents for 3D Printable and Self-Healable Ionogels. *ACS Applied Polymer Materials* **2022**, 4 (12), 9221-9230.

(S3) Xiang, Z.; Li, N.; Rong, Y.; Zhu, L.; Huang, X. 3D-printed high-toughness double network hydrogels via digital light processing. *Colloids and Surfaces A: Physicochemical and Engineering Aspects* **2022**, 639, 128329.

(S4) Pal, S.; Su, Y.-Z.; Chen, Y.-W.; Yu, C.-H.; Kung, C.-W.; Yu, S.-S. 3D Printing of Metal–Organic Framework-Based Ionogels: Wearable Sensors with Colorimetric

and Mechanical Responses. *ACS Applied Materials & Interfaces* **2022**, 14 (24), 28247-28257.

(S5) Yoshida, D.; Park, J.; Yamashita, N.; Ikura, R.; Kato, N.; Kamei, M.; Ogura, K.; Igarashi, M.; Nakagawa, H.; Takashima, Y. Preparation of mechanically tough poly(dimethyl siloxane) through the incorporation of acetylated cyclodextrin-based topologically movable cross-links. *Polymer Chemistry* **2023**, 3277-3285.

(S6) Wang, Z.; Heck, M.; Yang, W.; Wilhelm, M.; Levkin, P. A. Tough PEGgels by In Situ Phase Separation for 4D Printing. *Advanced Functional Materials* **2023**, 2300947.

(S7) Silvaroli, A. J.; Heyl, T. R.; Qiang, Z.; Beebe, J. M.; Ahn, D.; Mangold, S.; Shull, K. R.; Wang, M. Tough, Transparent, Photocurable Hybrid Elastomers. *ACS Applied Materials & Interfaces* **2020**, 12 (39), 44125-44136.

(S8) Perera, S. D.; Durand-Silva, A.; Remy, A. K.; Diwakara, S. D.; Smaldone, R. A. 3D Printing of Aramid Nanofiber Composites by Stereolithography. *ACS Applied Nano Materials* **2022**, 5 (10), 13705-13710.

(S9) Fang, H.; Guymon, C. A. Thermo-mechanical properties of urethane acrylate networks modulated by RAFT mediated photopolymerization. *Polymer* **2022**, 256, 125197.

(S10) Beigi, S.; Yeganeh, H.; Atai, M. Evaluation of fracture toughness and mechanical properties of ternary thiol-ene-methacrylate systems as resin matrix for dental restorative composites. *Dental Materials* **2013**, 29 (7), 777-787.

(S11) Chu, X.; Tu, J. W.; Berensmann, H. R.; La Scala, J. J.; Palmese, G. R. High T-g, Bio-Based Isosorbide Methacrylate Resin Systems for Vat Photopolymerization. *Polymers* **2023**, 15 (9).

(S12) Ge, M. Y.; Miao, J. T.; Zhang, K.; Wu, Y. D.; Zheng, L. H.; Wu, L. X. Building biobased, degradable, flexible polymer networks from vanillin via thiol-ene "click" photopolymerization. *Polymer Chemistry* **2021**, 12 (4), 564-571.

(S13) Kuenstler, A. S.; Hernandez, J. J.; Trujillo-Lemon, M.; Osterbaan, A.; Bowman, C. N. Vat Photopolymerization Additive Manufacturing of Tough, Fully Recyclable Thermosets. *ACS Applied Materials & Interfaces* **2023**, 15 (8), 11111-11121.

(S14) Childress, K. K.; Alim, M. D.; Hernandez, J. J.; Stansbury, J. W.; Bowman, C. N. Additive manufacture of lightly crosslinked semicrystalline thiol–enes for enhanced mechanical performance. *Polymer Chemistry* **2020**, 11 (1), 39-46.

(S15) Bhanushali, H.; Mestry, S.; Mhaske, S. T. Castor oil-based UV-curable polyurethane acrylate resins for digital light processing (DLP) 3D printing technology. *Journal of Applied Polymer Science* **2023**, 140 (18), e53817,

(S16) Graf, D.; Qazzazie, A.; Hanemann, T. Investigations on the Processing of Ceramic Filled Inks for 3D InkJet Printing. *Materials*, **2020**; 13, (11), 2587

(S17) Gao, G.; Wang, X.; Chen, M.; Bowman, C. N.; Stansbury, J. W. Functional Nanogels as a Route to Interpenetrating Polymer Networks with Improved Mechanical Properties. *Macromolecules* **2021**, 54 (23), 10657-10666.

(S18) Sangermano, M.; Priola, A.; Malucelli, G.; Bongiovanni, R.; Quaglia, A.; Voit, B.; Ziemer, A. Phenolic Hyperbranched Polymers as Additives in Cationic Photopolymerization of Epoxy Systems. *Macromolecular Materials and Engineering* **2004**, 289 (5), 442-446.

(S19) Commisso, A. J.; Sama, G. R.; Scott, T. F. Radical-Mediated Ring-Opening Photopolymerization for Semicrystalline Thermoplastic Additive Manufacturing. *Chemistry of Materials* **2023**, 35 (10), 3825-3834.

(S20) Huang, W.; Luo, Q.; Zhu, Y.; Liu, X.; Xiang, H. Modified rod-shaped calcium carbonate with thiols improving UV-curing 3D printing resin. *Journal of Applied Polymer Science* **2022**, 139 (47), e53185.

(S21) Guit, J.; Tavares, M. B. L.; Hul, J.; Ye, C.; Loos, K.; Jager, J.; Folkersma, R.; Voet, V. S. D. Photopolymer Resins with Biobased Methacrylates Based on Soybean Oil for Stereolithography. *ACS Applied Polymer Materials* **2020**, 2 (2), 949-957.

(S22) Wang, X.; Hernandez, J. J.; Gao, G.; Stansbury, J. W.; Bowman, C. N. Poly(triazole) Glassy Networks via Thiol-Norbornene Photopolymerization: Structure–Property Relationships and Implementation in 3D Printing. *Macromolecules* **2021**, 54 (9), 4042-4049.

(S23) Lee, K.; Shang, Y.; Bobrin, V. A.; Kuchel, R.; Kundu, D.; Corrigan, N.; Boyer, C. 3D Printing Nanostructured Solid Polymer Electrolytes with High Modulus and Conductivity. *Advanced Materials* **2022** 34 (42), 2204816.

(S24) Bae, S.-U.; Kim, B.-J. Effects of Cellulose Nanocrystal and Inorganic Nanofillers on the Morphological and Mechanical Properties of Digital Light Processing (DLP) 3D-Printed Photopolymer Composites. *Applied Sciences*, **2021**; 11(15), 6835.

(S25) Grover, T. L.; Guymon, C. A. Controlling network morphology in hybrid radical/cationic photopolymerized systems. *Polymer Chemistry* **2023**, 14 (2), 126-136.

(S26) Fang, C.; Li, N.; Liu, Y.; Lu, G. Toughening epoxy acrylate with polyurethane acrylates and hyper-branched polyester in three dimensional printing. *Materials Research Express* **2018**, 5 (5), 055307.

(S27) Yu, B.; He, J. W.; Garoushi, S.; Vallittu, P. K.; Lassila, L. Enhancing Toughness and Reducing Volumetric Shrinkage for Bis-GMA/TEGDMA Resin Systems by Using Hyperbranched Thiol Oligomer HMDI-6SH. *Materials* **2021**, 14(11): 2817.

(S28) Dellago, B.; Altun, A. A.; Liska, R.; Baudis, S. Exploring the limits of toughness enhancers for 3D printed photopolymers as bone replacement materials. *Journal of Polymer Science* **2023**, 61 (2), 143-154.

(S29) Fang, Z.; Lu, R.; Chen, J.; Zhao, Q.; Wu, J. Vat photopolymerization of tough glassy polymers with multiple shape memory performances. *Additive Manufacturing* **2022**, 59, 103171.

(S30) Kopatz, J. W.; Unangst, J.; Cook, A. W.; Appelhans, L. N. Compositional effects on cure kinetics, mechanical properties and printability of dual-cure epoxy/acrylate resins for DIW additive manufacturing. *Additive Manufacturing* **2021**, 46, 102159.

(S31) Gorsche, C.; Seidler, K.; Harikrishna, R.; Kury, M.; Koch, T.; Moszner, N.; Liska, R. Difunctional vinyl sulfonate esters for the fabrication of tough methacrylate-based photopolymer networks. *Polymer* **2018**, 158, 149-157.

(S32) Demleitner, M.; Schönl, F.; Angermann, J.; Fässler, P.; Lamparth, I.; Rist, K.; Schnur, T.; Catel, Y.; Rosenfeldt, S.; Retsch, M.; et al. Influence of Block Copolymer Concentration and Resin Crosslink Density on the Properties of UV-Curable Methacrylate Resin Systems. *Macromolecular Materials and Engineering* **2022**, 307 (10), 2200320.

(S33) Peer, G.; Eibel, A.; Gorsche, C.; Catel, Y.; Gescheidt, G.; Moszner, N.; Liska, R. Ester-Activated Vinyl Ethers as Chain Transfer Agents in Radical Photopolymerization of Methacrylates. *Macromolecules* **2019**, 52 (7), 2691-2700.

(S34) Wang, H.; Huang, Z.; Zhang, Y.; Li, L.; Li, J. Design of enhanced mechanical properties by interpenetrating network of 3D printing dual-curing resins. *Polymer* **2023**, 282, 126153.

(S35) Grunenberg, D.; Ehrmann, K.; Gorsche, C.; Steyrer, B.; Koch, T.; Stampfl, J.; Liska, R. Heterotelechelic poly(propylene oxide) as migration-inhibited toughening agent in hot lithography based additive manufacturing. *Polymer Chemistry* **2021**, 12 (9), 1260-1272.

(S36) Lu, Y.; Han, X. X.; Gleadall, A.; Zhao, L.-G. Fracture Toughness of Three-Dimensional Stereolithography Printed Polymer Reinforced with Continuous Carbon Fibers. *3D Printing and Additive Manufacturing* **2022**, 9 (4), 278 – 287.

(S37) Idrees, M.; Yoon, H.; Palmese, G. R.; Alvarez, N. J. Engineering Toughness in a Brittle Vinyl Ester Resin Using Urethane Acrylate for Additive Manufacturing. *Polymers* **2023**, 15 (17), 3501.

(S38) Tu, J.; Makarian, K.; Alvarez, N. J.; Palmese, G. R. Formulation of a Model Resin System for Benchmarking Processing-Property Relationships in High-Performance Photo 3D Printing Applications. *Materials* **2020**, 13 (18), 4109.

(S39) Chen, R.; Cai, J.; Chin, K. C. H.; Wang, S.; Boydston, A. J.; Thevamaran, R.; Gopalan, P. Block copolymer additives for toughening 3D printable epoxy resin. *Giant* **2024**, 100204.
