# Supplementary material for: From Unregulated Networks to Designed Microstructures: Introducing Heterogeneity at Different Length Scales in Photopolymers for Additive Manufacturing
Source: Chem Rev. 2024 Mar 28;124(7):3978–4020. doi: 10.1021/acs.chemrev.3c00570 (PMC11009961; doi:10.1021/acs.chemrev.3c00570)
Supplement: Supplementary file 2 — cr3c00570_si_002.pdf [file cr3c00570_si_002.pdf]

Supporting Information

From unregulated networks to designed microstructures: Introducing heterogeneity at different length scales in photopolymers for additive manufacturing

Mojtaba Ahmadi,[1,§] Katharina Ehrmann,[2,§,]* Thomas Koch,[1] Robert Liska,[2] Jürgen Stampfl[1,]*

[1] Institute of Materials Science and Technology, Technische Universität Wien, Gumpendorfer Strasse 7, 1060 Vienna, Austria

[2] Institute for Applied Synthetic Chemistry, Technische Universität Wien, Getreidemarkt 9/163, 1060 Vienna, Austria

[§] These authors contributed equally.

* Corresponding Authors
Email: katharina.ehrmann@tuwien.ac.at, juergen.stampfl@tuwien.ac.at

Abbreviation	Full Name
ABS	Acrylonitrile-butadiene-styrene
AFM	Atomic Force Microscopy
AMT	Additive Manufacturing Technology
ASTM	American Society for Testing and Materials
ATB	Amino-terminated polybutadiene
ATBN	Amino-terminated polybutadiene/acrylo-nitrile
BA	Butyl acrylate
BAPO	Bis-acyl phosphine oxide
CAD	Computer-aided design
CDM-DGE	Cyclohexane dimethanol diglycidyl ether
CLSM	Confocal laser scanning microscopy
CPMs	Cyclopolymerizable monomers
CSPs	Core-shell particles
CTA	Chain transfer agents
CTBN	Carboxyl-terminated butadiene-acrylonitrile
CTOD	Crack tip opening displacement
DBC	Double-bond conversion
DBN	1,5-Diazabicyclo [4.3.0] non-5-ene
DBU	1,8-Diazabicyclo [5.4.0] undec-7-ene
DEA	Data envelopment analysis
DGEBA	Diglycidyl ether bisphenol A
DIW	Direct ink writing

[Table is continued on page S2]

[Continuation from page S1]

**Abbreviation**	**Full Name**
DLP	Digital light processing
DLW	Direct laser writing
DMA	Dynamic mechanical analysis
DMTA	Dynamic mechanical thermal analysis
DOD	Drop on demand
DP	Degree of polymerization
DSC	Differential scanning calorimetry
ECC	3,4-Epoxycyclohexylmethyl-3',4'-epoxycyclohexane carboxylate
EHMA	Ethylhexyl methacrylate
EHOX	3-Ethyl-3-[(2-ethylhexyloxy) methyl] oxetane
ETB	Epoxy-terminated butadiene
ETBN	Epoxy-terminated butadiene-nitrile
FFF	Fused filament fabrication
FIB	Focused ion beam
FTIR	Fourier transform infrared spectroscopy
FWHM	Full width at half maximum
GTM	Glycerol trimethacrylate
HDDA	Hexanediol diacrylate
HDT	Heat deflection temperature
HEMA	Hydroxyethyl methacrylate
IBO(M)A	Isobornyl(meth)acrylate
IPC	Ion pair comonomer
IPN	Interpenetrating polymer network
LED	Light emitting diode
MIR	Mid-infrared
N&G	Nucleation and growth
NDS	Network disassembly spectrometry
NMR	Nuclear magnetic resonance
NSE	Neutron spin echo
NSOM	Near-field scanning optical microscopy
Oh	Ohnesorge number
OXMA	(3-ethyloxetane-3-yl) methyl acrylate
PB	Polybutadiene
PDMS	Polydimethylsiloxane
PEGDMA	Poly (ethylene glycol) dimethacrylate
PET(M)A	Pentaerythritol tetra(meth)acrylate
PETA	Pentaerythritol tetraacrylate
P-GE	Phenyl glycidyl ether
PhIPS	Photopolymerization induced phase separation
PiFM	Photo-induced force microscopy
PMMA	Polymethyl methacrylate
PPG	Polypropylene glycol

[Table is continued on page S3]

[Continuation from page S2]

PS	Polystyrene
PTFE	Poly(tetrafluoroethylene)
RAFT	Reversible addition-fragmentation chain transfer
R-DGE	Resorcinol diglycidyl ether
Re	Reynolds number
RT-NIR	Real time near-infrared
SAN	Styrene-acrylonitrile
SANS	Small-angle neutron scattering
SAXS	Small-angle X-ray scattering
SBS	Styrene-butadiene-styrene
SD	Spinodal decomposition
SLA	Stereolithography
SMP	Shape memory polymers
SNOM	Scanning near-field optical microscopy
TBD	1,5,7-Triazabicyclo (4.4.0) dec-5-en
TEGDMA	Triethylene glycol dimethacrylate
TEM	Transmission electron microscopy
TMG	1,1,3,3-Tetramethylguanidin
TMPT(M)A	Trimethylolpropane tri(meth)acrylate
TPO	Triphenyl phosphine oxide
TP-TGE	Trimethylolpropane triglycidyl ether
VBTOB-SS	Vinylbenzyltrioctylphosphonium 4-styrenesulfonate
VTB	Vinyl-terminated butadiene
VTBN	Vinyl-terminated butadiene-nitrile
We	Weber number
xDT-DAT	Thiol-ene - diallyl terephthalate

[End of table]
